# Dolutegravir as First- or Second-line Treatment for Children With HIV: 240-Week Follow-up of the ODYSSEY Randomized Trial

**DOI:** 10.1093/ofid/ofag266

**Published:** 2026-05-05

**Authors:** Hilda Angela Mujuru, Ellen White, Adeodata R Kekitiinwa, Abbas Lugemwa, Cissy Kityo, Avy Violari, Grace Miriam Ahimbisibwe, Ebrahim Variava, Moherndran Archary, Mark Cotton, Pradthana Ounchanum, Thanyawee Puthanakit, Suparat Kanjanavanit, Chaiwat Ngampiyaskul, Robin Kobbe, Srini Bandi, Cristina Epalza, Mutsawashe Bwakura-Dangarembizi, Dickson Bbuye, Mariam Kasozi, Elizabeth Kaudha, Tim R Cressey, Carlo Giaquinto, Lungile Jafta, Alasdair Bamford, Pablo Rojo, Diana M Gibb, Anna Turkova, Deborah Ford

**Affiliations:** University of Zimbabwe Clinical Research Centre, Harare, Zimbabwe; UCL Innovative Clinical Trials Unit (former MRC CTU at UCL), Institute of Clinical Trials and Methodology, University College London, London, UK; Baylor College of Medicine Children's Foundation Uganda, Kampala, Uganda; Joint Clinical Research Centre, Mbarara, Uganda; Joint Clinical Research Centre, Kampala, Uganda; Faculty of Health Sciences, Perinatal HIV Research Unit, University of the Witwatersrand, Johannesburg, South Africa; Makerere University–Johns Hopkins University Research Collaboration, Kampala, Uganda; Faculty of Health Sciences, Perinatal HIV Research Unit, University of the Witwatersrand, Johannesburg, South Africa; Department of Paediatrics, Victoria Mxenge Hospital, Enhancing Care Foundation, Durban, South Africa; Department of Paediatrics and Child Health, Family Center for Research With Ubuntu, Stellenbosch University, Cape Town, South Africa; Chiangrai Prachanukroh Hospital, Chiangrai, Thailand; HIVNAT Thai Red Cross AIDS Research Center and Department of Pediatrics, Chulalongkorn University, Bangkok, Thailand; Nakornping Hospital, Chiang Mai, Thailand; Prapokklao Hospital, Chanthaburi, Thailand; Institute for Infection Research and Vaccine Development, University Medical Centre Hamburg-Eppendorf, Hamburg, Germany; Department of Infectious Disease Epidemiology, Bernhard Nocht Institute for Tropical Medicine, Hamburg, Germany; University Hospitals of Leicester NHS Trust, Leicester, UK; Universidad Complutense, Hospital Universitario 12 de Octubre, Madrid, Spain; University of Zimbabwe Clinical Research Centre, Harare, Zimbabwe; Baylor College of Medicine Children's Foundation Uganda, Kampala, Uganda; Joint Clinical Research Centre, Mbarara, Uganda; Joint Clinical Research Centre, Kampala, Uganda; AMS-PHPT Research Collaboration, Department of Medical Technology, Faculty of Associated Medical Sciences, Chiang Mai University, Chiang Mai, Thailand; Fondazione Penta ETS, Padua, Italy; University of Padua, Padua, Italy; Fondazione Penta ETS, Padua, Italy; UCL Innovative Clinical Trials Unit (former MRC CTU at UCL), Institute of Clinical Trials and Methodology, University College London, London, UK; Department of Paediatric Infectious Diseases, Great Ormond Street Hospital for Children NHS Foundation Trust, London, UK; UCL Great Ormond Street Institute of Child Health, London, UK; Universidad Complutense, Hospital Universitario 12 de Octubre, Madrid, Spain; UCL Innovative Clinical Trials Unit (former MRC CTU at UCL), Institute of Clinical Trials and Methodology, University College London, London, UK; UCL Innovative Clinical Trials Unit (former MRC CTU at UCL), Institute of Clinical Trials and Methodology, University College London, London, UK; UCL Innovative Clinical Trials Unit (former MRC CTU at UCL), Institute of Clinical Trials and Methodology, University College London, London, UK

**Keywords:** children, dolutegravir, ODYSSEY trial, pediatrics, randomized trial

## Abstract

**Background:**

The World Health Organization recommends dolutegravir (DTG) as the anchor antiretroviral therapy (ART) drug for children with HIV.

**Methods:**

The ODYSSEY randomized trial evaluated DTG-based ART vs non-DTG standard of care (SOC) over 96 weeks in children starting first-line ART or switching to second-line. Participant follow-up was extended to 240 weeks; ART in extended follow-up was per national guidelines. Flexible parametric survival models estimated the proportion of participants with treatment failure on randomized allocation, accounting for treatment switches through censoring and inverse probability of censoring weights. Adverse events were analyzed in the intention-to-treat population.

**Results:**

ODYSSEY enrolled 792 participants (392 DTG, 400 SOC): 311 started first-line (SOC, 92% efavirenz) and 396 second-line (SOC, 98% ritonavir-boosted protease inhibitors). The median (range) age at enrollment was 11.4 years (0.1–18.0); weight was 28.7 kg (3.4–85.0); and 89% were from Africa. Median follow-up on randomized allocation was 286 weeks (IQR, 230–310) for DTG and 204 weeks (168–240) for SOC. Among SOC participants, 23% switched to DTG before 192 weeks, 65% before 240 weeks. By 240 weeks, 74 participants on DTG and 131 on SOC had treatment failure (estimated, 19% vs 34%; difference [DTG-SOC], −15%; 95% CI, −21% to −9%; *P* < .001). Treatment effects were similar between first- and second-line (*P* = .10). The proportions of participants with serious adverse events (52 DTG vs 53 SOC, *P* = .92) or grade ≥3 adverse events (98 DTG vs 121 SOC, *P* = .09) were similar by arm over 192 weeks.

**Conclusions:**

DTG showed superior efficacy vs SOC over 240 weeks in children, with no safety concerns.

## Teaser

ODYSSEY extended follow-up confirmed the superiority of dolutegravir-based antiretroviral therapy over standard of care for ∼4.5 years in infants, children, and adolescents, with lower risk of treatment failure. It also reaffirmed safety in the overall population and across first- and second-line populations, supporting World Health Organization recommendations for dolutegravir as preferred pediatric treatment.

Worldwide among children and adolescents aged 0 to 19 years, an estimated 2.4 million have HIV [1]. Treatment outcomes remain worse in children than adults: in 2024, an estimated 86% of children undergoing antiretroviral therapy (ART) globally were virologically suppressed at <1000 copies/mL (c/mL) as compared with ∼95% of adults [1].

In 2021, the ODYSSEY randomized trial reported superior efficacy of dolutegravir (DTG)–based ART as compared with then current non–DTG-based standard of care (SOC) over 96 weeks in children weighing ≥14 kg starting first-line ART (SOC, 92% efavirenz) or second-line ART (SOC, 97% boosted protease inhibitor). Similar results were reported for children weighing <14 kg the following year (SOC, 74% lopinavir/ritonavir) [2, 3]. ODYSSEY data contributed to successful licensing submissions for pediatric DTG formulations and dosing recommendations and strengthened support for the World Health Organization's (WHO's) recommendation for DTG-based ART as preferred treatment for all children with HIV. Since early 2021, DTG for children has rolled out globally, starting in older children, resulting in ∼85% of children prescribed ART receiving DTG-based regimens in 2023 [4].

Programmatic data from low- and middle-income countries demonstrate high virologic suppression (≥95%, <1000 c/mL) at 96 weeks in adults receiving WHO-recommended first-line DTG-based ART regimens, with similar rates of suppression in those starting ART and those transitioning from another regimen [5], but data beyond 2 years are limited. Programmatic data for children who were ART-naive or ART-experienced and starting DTG-based ART were captured by a study conducted in Europe and Thailand. These data revealed virologic suppression rates of 88% to 91% (<200 c/mL) at 48-week time points from 48 to 192 weeks, with a lower risk of virologic failure with DTG than a protease inhibitor–based regimen [6]. Pediatric data from African settings, while encouraging, are predominantly cross-sectional or have limited follow-up (<96 weeks) and are mostly based on adult DTG doses (suitable for children ≥20 kg) [7, 8].

Here we report the efficacy and safety of DTG-based ART vs SOC over 240 weeks in infants, children, and adolescents starting first-line ART or switching to second-line ART in the ODYSSEY trial. We also describe virologic suppression in SOC participants who switched to DTG in extended follow-up following national treatment guidelines. Anthropometric, body fat percentage, and metabolic outcomes up to 240 weeks have been reported [9].

## METHODS

### Study Design and Participants

ODYSSEY (ClinicalTrials.gov NCT02259127) was an open-label, multicenter, randomized clinical trial that compared the efficacy and safety of DTG-based ART (DTG plus 2 nucleoside/nucleotide reverse transcriptase inhibitors [NRTIs]; DTG arm) with then current non–DTG-based SOC (a nonnucleoside reverse transcriptase inhibitor, a boosted protease inhibitor, or a non-DTG integrase strand transfer inhibitor [INSTI] plus 2 NRTIs; SOC arm) in children aged ≥4 weeks and <18 years starting first-line ART (ODYSSEY A) or switching to second-line ART after having treatment failure (ODYSSEY B) [2, 3, 10]. Participants enrolled in ODYSSEY B had an HIV-1 RNA viral load (VL) of at least 500 c/mL within the 4 weeks up to and including screening. Second-line ART included a new anchor drug and at least 1 NRTI presumed to have activity, based on treatment history and resistance testing when available.

In the main trial, 707 children weighing ≥14 kg were recruited between 20 September 2016 and 22 June 2018. After DTG dispersible tablets became available for younger children, an additional 85 children weighing <14 kg were recruited between 5 July 2018 and 26 August 2019. The randomized phase was until the last participant reached week 96 (≥14-kg cohort, 24 April 2020; <14-kg cohort, 28 June 2021). Participants in Africa and Thailand were offered extended follow-up until 1 May 2023, when the last participant in the ≥14-kg cohort reached 240 weeks and the last participant in the <14-kg cohort reached 192 weeks; all participants then transferred to national programs. During extended follow-up, children in the SOC arm transitioned to DTG following changes in national guidelines.

### Procedures

Children were seen at screening, enrollment, weeks 4 and 12, and then every 12 weeks in randomized follow-up and at least every 24 weeks in extended follow-up. At each visit, adverse events and adherence were assessed, and ART was dispensed. In randomized and extended follow-up phases, real-time VL testing was done per local practice (every 24–48 weeks in African countries), with results returned to treating clinicians for clinical management. In the randomized phase, additional retrospective VL testing was done on stored samples for visits where real-time results were not available and used for safety oversight by the Independent Data Monitoring Committee and for trial outcome evaluation. CD4 was measured at baseline, weeks 4 and 24, and then every 24 weeks in the randomized phase and according to local practice in extended follow-up (typically every 48 weeks).

### Outcomes

The ODYSSEY trial's primary outcome was treatment failure by 96 weeks in the intention-to-treat (ITT) population and has been reported [2, 3]. Treatment failure was a composite endpoint including virologic or clinical failure. Virologic failure was defined as either of the following: a decrease of <1 log_10_ in VL at week 24 (or a VL ≥50 c/mL at week 24 if <500 c/mL at baseline), with a switch to second- or third-line ART for treatment failure; 2 consecutive VL results ≥400 c/mL, with the first occurring at or after week 36. Clinical failure was defined as a new or recurrent WHO stage 4 or severe WHO stage 3 event or death from any cause.

Here we report the same efficacy and safety outcomes originally assessed as primary and secondary outcomes at 96 weeks, now extended to 240 weeks encompassing the randomized and extended follow-up phases. Efficacy outcomes include treatment failure by 48, 96, 144, 192, and 240 weeks; cross-sectional VL <50, <400, or <1000 c/mL at the same time points; and change in CD4 count and lymphocyte percentage from baseline to 240 weeks. Safety outcomes include serious adverse events, new clinical and laboratory adverse events of grade ≥3, and adverse events of any grade leading to treatment modification, as well as severe WHO stage 3 event, WHO stage 4 event, or death.

Viral suppression in participants in the SOC arm switching to DTG was defined as a VL <400 c/mL in the most recent sample within 24 weeks preswitch and 48 weeks postswitch.

### Statistical Analysis

Analyses were conducted in the total trial population, including participants enrolled in the ≥14- and <14-kg cohorts and those starting first- and second-line ART. Follow-up was censored on 1 May 2023 or last outcome measurement, whichever occurred earlier.

Efficacy analyses estimate the effects of being prescribed DTG vs alternative non–DTG-based ART (ie, effects assuming that participants remained “on randomized allocation”). Participants and estimates are described as “on DTG” or “on SOC” to align with their prescribed ART, although no adjustments are made for adherence levels. These differ from descriptions by trial arm which are described as “in DTG arm” or “in SOC arm” or “intention-to-treat”. Treatment regimens of interest were defined as DTG-based ART for a participant assigned to the DTG arm and non–DTG-based ART for a participant assigned to the SOC arm. Participants were artificially censored after they switched from their randomized allocation. Inverse probability weights were used to adjust for artificial censoring. Weights were also applied to account for censoring due to death, except for analysis of treatment failure; loss to follow-up; and administrative censoring, due to exit at the end of randomized phase. Weights adjusted for most recent weight, body mass index (BMI) for age, CD4 (CD4% for <14-kg cohort), and log_10_ VL.

Marginal probabilities of treatment failure and absolute differences between DTG and SOC in the trial population were estimated by flexible parametric survival models fitted on the log cumulative hazard scale with restricted cubic splines [11, 12]. We adjusted for baseline covariates including ODYSSEY A (first-line) or B (second-line), country or region month and year of randomization (spline), sex, age, weight, BMI-for-age *Z* score, CD4 count, and log_10_ VL.

Mean changes in CD4/CD4% from baseline and differences between DTG and SOC were estimated with linear mixed models. Models included a random intercept for participant and fixed effects for trial arm and visit week (spline), with interaction terms between trial arm and visit week. Models also adjusted for the outcome measure at baseline and interactions with visit week spline terms [13] and the same baseline covariates used in the treatment failure models. Proportions of participants with VL <50, 400, and 1000 c/mL were estimated similarly by mixed logistic models.

Analyses on randomized allocation are reported to week 240, except where we present results separately for the <14-kg cohort, when we report to week 192. Subgroup differences (ODYSSEY A vs B; ≥14 vs <14 kg) in the effect of ART regimen on the risk of treatment failure were estimated with data to each 48-week time point. For other efficacy outcomes (cross-sectional VL, CD4, CD4%), heterogeneity in treatment effects were estimated from longitudinal mixed models (logistic (VL) and linear (CD4, CD4%)) across all time points.

Cox models were used to compare time to first adverse event between treatment arms in the ITT population up to 192 weeks (censoring before the majority of the SOC arm switched to DTG). Weighted analyses of adverse events were not considered clinically relevant. Models adjusted for ODYSSEY A or B in the total population and ≥14-kg cohort analyses. Full details of statistical methods are available in the Supplementary material.

### Patient Consent Statement

To participate in the ODYSSEY trial, carers or children above the national legal age of consent gave written informed consent. Additional assent was obtained for children below the national legal age of consent, where appropriate. Carers and children reconsented or reassented for extended follow-up. Ethics approvals were obtained as required locally as follows: Uganda, Uganda National Council for Science and Technology (HS1989); Zimbabwe, Joint Research Ethics Committee for the University of Zimbabwe, College of Health Sciences, and Parirenyatwa Group of Hospitals (JREC 202/15); South Africa, the Health Research Ethics Committee, Stellenbosch University (M15/07/022), Human Research Ethics Committee, University of Witwatersrand, Johannesberg (150601B), Pharma-Ethics (160914634); Thailand, the Ethical Review Committee for Research in Human Subjects, the Ministry of Public Health (27/2558), Institutional Review Board of the Faculty of Medicine, Chulalogkorn University, Bangkok (331/58); Germany, Ethics Committee, Goethe University Frankfurt (417/16); United Kingdom, National Research Ethics Service Committee London-Riverside, Health Research Authority (15/LO/1120); Spain, the Research Ethics Committee for Medicines of the Doce de Octubre University Hospital (CEIC, 16/003); Portugal, National Ethics Committee for Clinical Research (CEIC, 20170366).

## RESULTS

A total of 792 children were randomized in ODYSSEY (392 DTG, 400 SOC): 707 in the ≥14-kg cohort and 85 in the <14-kg cohort (Supplementary Figures 1 and 2, Supplementary Table 1). Overall, 383 started first-line ART (ODYSSEY A cohort) and 409 initiated second-line (ODYSSEY B); 389 (49%) were female. Furthermore, 374 (47%) participants were enrolled in Uganda, 168 (21%) in Zimbabwe, 164 (21%) in South Africa, 61 (8%) in Thailand, and 25 (3%) in Europe. At enrollment, the median age was 11.4 years (range, 0.1–18.0), and the median weight was 28.7 kg (range, 3.4–85.0; Table 1). The median CD4 count was 494 cells/mm^3^ (IQR, 248–814), and 34% (268/788) had CD4 <350 cells/mm^3^. The median RNA VL (log_10_) was 4.5 c/mL (IQR, 3.9–5.1).

**Table 1.** Baseline Characteristics in the Total Population

	DTG (n = 392)		SOC (n = 400)		Total (N = 792)	
	No.	%	No.	%	No.	%
ODYSSEY A/B						
A	189	48	194	48	383	48
B	203	52	206	52	409	52
Weight cohort						
≥14 kg	350	89	357	89	707	89
<14 kg	42	11	43	11	85	11
Country/region of residence						
Europe	12	3	13	3	25	3
South Africa	69	18	95	24	164	21
Thailand	28	7	33	8	61	8
Uganda	192	49	182	46	374	47
Zimbabwe	91	23	77	19	168	21
Sex						
Female	200	51	189	47	389	49
Age, y						
Median; IQR	11.2	8.4–14.8	11.5	7.6–14.4	11.4	8.0–14.6
Range		0.3–18.0		0.1–18.0		0.1–18.0
Weight, kg						
Median; IQR	28.2	21.2–41.9	29.1	20.6–41.0	28.7	20.9–41.5
Range		3.8–85.0		3.4–72.7		3.4–85.0
CD4 lymphocyte percentage^a^	391		397		788	
Median; IQR	20	12–29	22	13–31	21	12–30
<15	128	33	119	30	247	31
15–<30	174	45	165	42	339	43
≥30	89	23	113	28	202	26
CD4 lymphocyte count,^a^ cells/mm^3^	391		397		788	
Median; IQR	468	222–812	529	276–818	494	248–814
<50	40	10	38	10	78	10
50–<100	22	6	14	4	36	5
100–<200	29	7	22	6	51	6
200–<350	48	12	55	14	103	13
350–<500	72	18	62	16	134	17
500–<1000	109	28	131	33	240	30
1000–1500	40	10	53	13	93	12
≥1500	31	8	22	6	53	7
Viral load, copies/mL^a^						
<400	5	1	11	3	16	2
400–<1000	13	3	8	2	21	3
1000–<10 000	79	20	111	28	190	24
10 000–<50 000	124	32	115	29	239	30
50 000–<100 000	48	12	48	12	96	12
100 000–<500 000	94	24	76	19	170	22
500 000–<1 000 000	15	4	16	4	31	4
≥1 000 000	14	4	10	3	24	3
Log_10_ viral load, log_10_ copies/mL^a^	392		395		787	
Median; IQR	4.6	4.0–5.2	4.5	3.8–5.0	4.5	3.9–5.1
History of WHO staging^b^						
Stage 1	144	37	162	40	306	39
Stage 2	140	36	128	32	268	34
Stage 3	75	19	68	17	143	18
Stage 4	33	8	42	10	75	9
NRTI backbone at randomization						
ABC 3TC	270	69	268	67	538	68
ABC TDF	1	0	2	0	3	0
TDF/TAF 3TC/FTC^c^	80	20	84	21	164	21
ZDV 3TC	41	10	46	12	87	11
Anchor drug class at randomization						
INSTI	392	100	3	1	395	50
NNRTI	0	0	163	41	163	21
PI	0	0	234	58	234	30

Data are for participants randomized to the stated treatments, given as No. (%) unless noted otherwise. Percentages are for the nonmissing proportion.

Abbreviations: 3TC, lamivudine; ABC, abacavir; DTG, dolutegravir; FTC, emtricitabine; INSTI, integrase inhibitor; NNRTI, nonnucleoside reverse transcriptase inhibitor; NRTI, nucleoside reverse transcriptase inhibitor; PI, protease inhibitor; SOC, standard of care; TAF, tenofovir alafenamide; TDF, tenofovir disoproxil fumarate; WHO, World Health Organization; ZDV, zidovudine.

^a^At a participant level, the mean of the measured values was used if they were available at screening and randomization.

^b^Worst known stage prior to enrollment.

^c^Two ≥14-kg cohort participants in the SOC group initiated tenofovir alafenamide and emtricitabine (1 in ODYSSEY A and 1 in ODYSSEY B).

In ODYSSEY A, 81% (157/194) in the SOC arm initiated first-line ART based on a nonnucleoside reverse transcriptase inhibitor, and in ODYSSEY B, 96% (198/206) initiated an SOC regimen based on a protease inhibitor (Supplementary Table 2). NRTI backbones were balanced across randomized arms. In ODYSSEY A, 83% (319/383) received abacavir and lamivudine; in ODYSSEY B, 54% (219/409) received abacavir and lamivudine, 25% (103) tenofovir disoproxil fumarate and lamivudine or emtricitabine, and 20% (83) zidovudine and lamivudine.

A total of 726 participants were followed up to the end of the randomized phase, including 706 in Africa and Thailand, of whom 683 (97%) consented to extended follow-up. Of participants who entered extended follow-up, 611 (89%) attended a final study visit on or after 1 May 2023. The median total follow-up (randomized phase and extended follow-up) was 286 weeks (IQR, 234–310). Moreover, 95% participants in the DTG arm and 77% in the SOC arm remained on their randomized allocation at 192 weeks, with 94% and 35% at 240 weeks, respectively (Kaplan-Meier estimates; Supplementary Figures 3–5). Median follow-up on randomized allocation was 286 weeks (IQR, 230–310) for DTG and 204 weeks (168–240) for SOC.

### Efficacy

**Figure 1.**
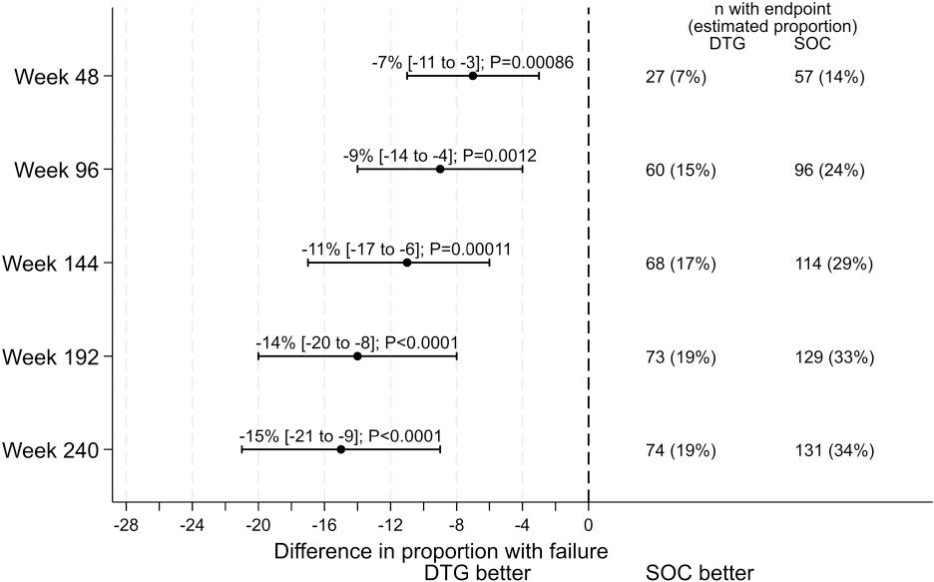
On randomized allocation: estimated difference in proportion of participants with clinical or virological failure by follow-up week. On-randomized allocation effects were estimated from marginal structural models with stabilized inverse probability weights to account for a switch off the randomized treatment regimen and censoring due to death, loss to follow-up, or administrative censoring (Supplementary Appendix, Supplementary Statistical Methods). Marginal probabilities of treatment failure and absolute differences between DTG and SOC in the trial population were estimated via flexible parametric survival models fitted on the log cumulative hazard scale with restricted cubic splines, adjusted for baseline covariates ODYSSEY A or B, country or region, month and year of randomization (spline), sex, age, weight, BMI-for-age *Z* score, CD4 count, and log_10_ viral load. Numbers of participants experiencing virologic failure contributing to the model at each specified time point are reported (ie, numbers remaining on randomized allocation); estimated proportions and treatment differences are estimated by weighting observations to account for participants switching off the randomized allocation and censoring. Data in brackets [ ] represent 95% CIs. Abbreviations: BMI, body mass index; DTG, dolutegravir; SOC, standard of care.

**Figure 2.**
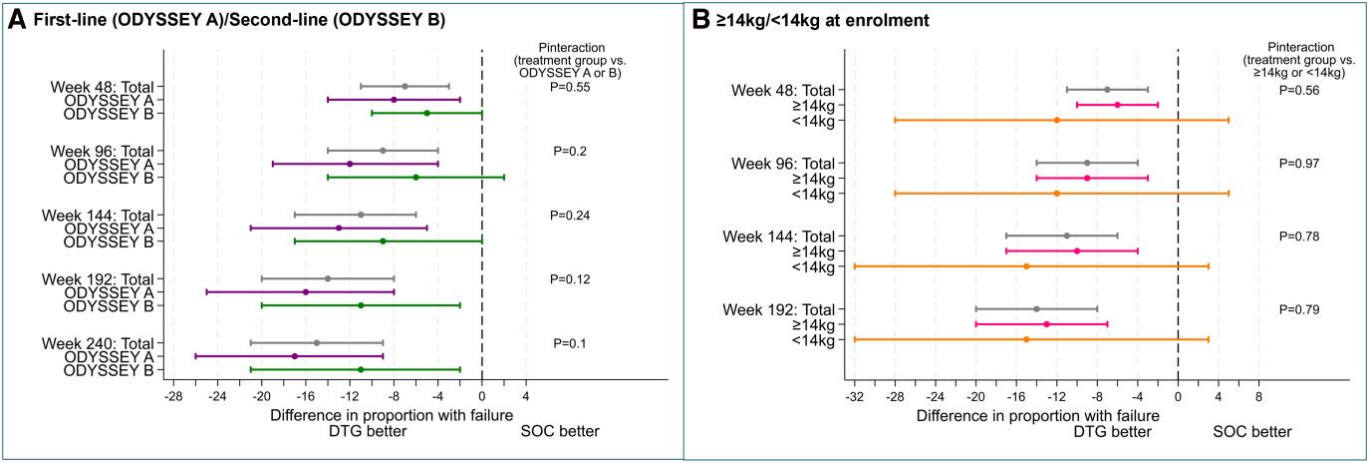
On randomized allocation: estimated difference in proportion of participants with clinical or virological failure by (A) ODYSSEY A/B and (B) weight cohorts (>=14kg/<14kg at enrollment). On-randomized allocation effects are reported to week 240, except where presented separately for the <14-kg cohort when we report to week 192. Subgroup differences (ODYSSEY A vs B; ≥14 vs <14 kg) in the effect of antiretroviral therapy regimen on the risk of treatment failure are estimated with data to each of the 48-week time points. Error bars indicate 95% CIs. DTG, dolutegravir; SOC, standard of care.

Seventy-four participants (estimated probability, 19%) experienced treatment failure on DTG by 240 weeks and 131 (34%) on SOC (treatment difference [95% CI], −15% [−21% to −9%]; *P* < .001; Figure 1, Table 2). Overall, 179 (87%) participants who met the primary endpoint experienced virologic failure (62/74 [84%] DTG, 117/131 [89%] SOC). Absolute treatment differences increased over time to 192 weeks, but there were only 3 failures between 192 and 240 weeks: 1 DTG and 2 SOC among 269 and 172 participants, respectively, who remained in follow-up on randomized allocation beyond 192 weeks and had not previously met the primary endpoint. Treatment effects were similar in the ODYSSEY A and B cohorts by 240 weeks (*P* = .10 for heterogeneity; Figure 2, Table 2, Supplementary Table 3) and similar in ≥14- and <14-kg cohorts by 192 weeks (*P* = .79 for heterogeneity), although probabilities of treatment failure were higher in the <14-kg cohort on DTG and SOC (26% DTG vs 41% SOC by 192 weeks compared with 18% vs 31% in the ≥14-kg cohort). Treatment effects in the ITT population were similar (Supplementary Table 4).

**Table 2.** On Randomized Allocation: Efficacy Endpoints Comparing Dolutegravir-Based ART With Standard of Care to Week 240

	DTG	SOC	Treatment Effect; *P* Value	Interaction *P* Value^a^
No. of participants randomized	392	400	…	…
Treatment failure by 240 wk	74	131		
Insufficient virologic response at 24 wk	0	3		
Confirmed viral load ≥400 c/mL at >36 wk	62	114		
Severe WHO stage 3 event	0	1		
WHO stage 4 event	9	7		
Death	3	6		
Estimated probability of treatment failure, % (95% CI)	19 (16–23)	34 (30–39)	−15% (−21 to −9); <.001	.10
Viral load at 240 wk				
<50 c/mL, No.	211/267	73/101		.23
Estimated proportion, % (95% CI)	78 (74–82)	67 (60–75)	11 (2–19); .018	
<400 c/mL, No.	230/267	84/102		.99
Estimated proportion, % (95% CI)	86 (82–90)	77 (71–84)	8 (1–16); .027	
<1000 c/mL, No.	235/267	86/102		.67
Estimated proportion, % (95% CI)	88 (84–91)	80 (74–86)	8 (1–15); .034	
Change from baseline to 240 wk				
CD4 count, No.	210	87		
Mean change,^b^ cells/mm^3^	164 ± 21	169 ± 29	−5 (−73 to 62); .88	.33
CD4%, No.	210	87		
Mean change,^b^ %	12.4 ± 0.5	11.4 ± 0.8	1.0 (−.9 to 2.9); .30	.19

On-randomized allocation effects were estimated from marginal structural models with stabilized inverse probability weights to account for switch off randomized treatment regimen and censoring due to death, loss to follow-up, or administrative censoring (Supplementary Appendix, Supplementary Statistical Methods). Marginal probabilities of treatment failure and absolute differences between DTG and SOC in the trial population were estimated by flexible parametric survival models fitted on the log cumulative hazard scale with restricted cubic splines, adjusted for baseline covariates including ODYSSEY A or B, country or region, month and year of randomization (spline), sex, age, weight, BMI-for-age *Z* score, CD4 count, and log_10_ viral load. Mean changes in CD4/CD4% from baseline and differences between DTG and SOC were estimated by linear mixed models. Models included a random intercept for participant and fixed effects for treatment regimen and visit week (spline), with interaction terms between treatment regimen and visit week. Models also adjusted for the outcome measure at baseline and interactions with visit week spline terms and the same baseline covariates used in the treatment failure models. Proportions of participants with viral load <50, 400, and 1000 c/mL were estimated similarly by mixed logistic models. Subgroup differences (ODYSSEY A vs B) in the effect of ART regimen on the risk of treatment failure were estimated with data up to 240 weeks. For other efficacy outcomes (cross-sectional viral load, CD4, CD4%), heterogeneity in treatment effects was estimated from longitudinal mixed models (logistic (VL) and linear (CD4, CD4%)) across all time points. Numbers of participants contributing to the model at specified time points are reported (ie, numbers remaining on randomized allocation with an observation available at each specified time point). Note that models include information across time points, using cubic splines for time, and observations are weighted to account for participants switching off randomized allocation and censoring.

Abbreviations: ART, antiretroviral therapy; BMI, body mass index; c/mL, copies/mL; DTG, dolutegravir; SOC, standard of care; WHO, World Health Organization.

^a^ *P* value for interaction: treatment group by first- or second-line therapy.

^b^Mean ± SE within the treatment groups.

At 240 weeks, 86% on DTG vs 77% on SOC had a VL <400 c/mL (*P* = .027), while at cutoffs of 50 and 1000 c/mL, 78% vs 67% (*P* = .018) and 88% vs 80% (*P* = .034) were suppressed, respectively (Table 2). Suppression was similarly higher on DTG at other 48-week time points (Supplementary Tables 5–7). There was no evidence of a difference between DTG and SOC in the increase in CD4 cell count and CD4% from baseline to 240 weeks (Table 2, Supplementary Figures 6 and 7).

Among participants who switched to DTG in the SOC trial arm before the end of extended follow-up, 90% (247/274) were virologically suppressed after the DTG switch (ODYSSEY A, 96% [110/115]; ODYSSEY B, 86% [137/159]), including 93% (178/192) of those with a preswitch VL <400 c/mL (ODYSSEY A, 99% [78/79]; ODYSSEY B, 88% [100/113]) and 83% (29/35) with a preswitch VL ≥400 c/mL (ODYSSEY A, 87% [13/15]; ODYSSEY B, 80% [16/20]; Supplementary Figure 8).

### Safety

By 192 weeks in the ITT population, there had been 13 new or recurrent severe WHO stage 3 or WHO stage 4 events or deaths in 11 participants in the DTG arm and 15 in 15 participants in the SOC arm (Table 3, Supplementary Tables 8 and 9). All events in the SOC arm were prior to the switch to DTG. These included 4 deaths in the DTG arm, from disseminated tuberculosis, traumatic event, chronic renal failure, and kwashiorkor, and 7 deaths in the SOC arm, from gastroenteritis, pneumonia (2 deaths, including 1 with presumed septicemia), pulmonary tuberculosis and severe malnutrition, severe malnutrition, non-Hodgkin lymphoma, and unknown cause (Supplementary Table 10).

**Table 3.** Intention to Treat: Safety Endpoints Comparing Dolutegravir With Standard of Care to 192 Weeks

	No. of Events [No. of Participants]			
	DTG (n = 392)	SOC (n = 400)	Treatment Effect: HR (95% CI); *P* Value	Interaction *P* Value^a^
Severe WHO stage 3, WHO stage 4, or death	13 [11]	15 [15]	0.74 (.34–1.60); .44	.96
Adverse events				
Serious	85 [52]	66 [53]	0.98 (.67–1.44); .92	.78
Grade ≥3	157 [98]	192 [121]	0.79 (.61–1.03); .087	.04
ART modifying	9 [8]	24 [21]	0.37 (.16–.84); .017	.20
Neuropsychiatric	20 [17]	16 [10]	1.69 (.78–3.70); .19	.57
Psychiatric	14 [12]	8 [5]	2.37 (.84–6.74); .1	.18
Neurologic	6 [6]	8 [6]	0.99 (.32–3.06); .98	.51

HRs are shown for time to first event, estimated by the Cox model; comparisons of treatment groups are adjusted for ODYSSEY A/B and are presented for the DTG group as compared with the SOC group.

Abbreviations: ART, antiretroviral therapy; DTG, dolutegravir; HR, hazard ratio; SOC, standard of care; WHO, World Health Organization.

^a^ *P* value for interaction: treatment group by first- or second-line therapy.

Similar proportions of participants in each trial arm had at least 1 serious adverse event up to 192 weeks (52 participants [13%] in the DTG arm and 53 [13%] in the SOC arm; adjusted hazard ratio [aHR], 0.98; 95% CI, .68–1.44; *P* = .92; Table 3, Supplementary Tables 8 and 9). Of the serious adverse events, 82% were considered serious due to hospitalization and 56% were due to infection (Supplementary Table 11). Furthermore, 98 participants (25%) in the DTG arm and 121 (30%) in the SOC arm had ≥1 adverse event of grade ≥3 (aHR, 0.79 [.61–1.03]; *P* = .087); the excess of adverse events in the SOC arm was observed in the ODYSSEY B cohort and was explained by elevated bilirubin levels in participants receiving atazanavir and ritonavir (Table 3, Supplementary Table 12). ART-modifying adverse events were less frequent in the DTG arm than SOC (8 participants [2%] DTG vs 21 [5%] SOC; aHR [95% CI], 0.37 [.16–.84]; *P* = .017; Table 3, Supplementary Table 13).

Overall, 27 participants (17 in the DTG arm and 10 in the SOC arm) had 36 neuropsychiatric adverse events (20 vs 16) by 192 weeks (aHR, 1.69; 95% CI, .78–3.70; *P* = .19; Table 3). Twelve participants (6 in the DTG arm and 6 in the SOC arm) had ≥1 neurologic event (aHR, 0.99; 95% CI, .32–3.06; *P* = .98). Seventeen participants (12 and 5) had ≥1 psychiatric event (aHR, 2.37; 95% CI, .84–6.74; *P* = .10; Table 3, Supplementary Table 14). Eight participants in the DTG arm had suicidal ideation or behavior as compared with 5 in the SOC arm (Supplementary Table 15). Other psychiatric events in the DTG arm included 2 diagnoses of depression (1 in a participant with suicidal behavior), 2 of violent/aggressive behavior, psychosis, and insomnia (2 separate events in same participant), and no additional psychiatric events were reported in the SOC arm.

Beyond 192 weeks, there were 6 psychiatric events in the DTG arm (all on DTG, except 1 suicide where the participant switched from DTG to atazanavir ∼2 years prior) and 3 in the SOC arm (including 1 case of psychosis in a participant switched to DTG ∼2.5 years prior and suicidal behavior in a participant who had switched to DTG ∼15 months prior).

## DISCUSSION

ODYSSEY was a landmark pediatric trial demonstrating superiority of DTG vs then SOC over 96 weeks in infants, children, and adolescents starting first-line ART or switching to second-line ART [2, 3]. Extended follow-up confirmed sustained superior efficacy over ∼4.5 years. Treatment failures were consistently lower on DTG in the overall population, first-line population (vs predominantly efavirenz-based SOC), second-line population vs predominantly boosted protease inhibitor-based SOC, and both weight cohorts (≥14 and <14 kg). Cross-sectional VL data further support less virologic rebound on DTG than on SOC through follow-up.

Although around 1 in 5 children on DTG experienced treatment failure by 240 weeks, our previous work showed that ∼60% of cases that fail virologically on DTG can achieve resuppression without changing ART regimen [14]. However, for the remainder who do not resuppress, it remains essential to consider appropriate treatment options.

Longer-term safety data on DTG were reassuring. Through follow-up, DTG was associated with a similar frequency of adverse events as SOC and fewer treatment changes for adverse events; yet, because the trial was open label, clinicians may have been more reluctant to switch participants off DTG than other ART regimens. Similar to during the randomized phase [15], risks of neuropsychiatric toxicities during extended follow-up were low in both arms, but the numerically higher risk of psychiatric toxicities on DTG continued, indicating that clinicians should remain vigilant to neuropsychiatric manifestations in children and adolescents with HIV. Over this ∼4.5-year period, we also reported better lipid profiles for DTG than SOC and reassuring data showing no differences in BMI-for-age *Z* scores, body fat gain, or central abdominal obesity [9].

Data comparing DTG triple ART against non–INSTI-based 3-drug regimens beyond 96 weeks are lacking in adults. The ADVANCE trial, which evaluated 2 DTG-based 3-drug regimens vs efavirenz-based triple ART in adults in South Africa, showed high virologic suppression <50 c/mL (≥98%) at 192 weeks in all 3 arms in participants who consented to remain in the trial beyond 96 weeks, but substantial numbers of participants discontinued at 96 weeks, with more discontinuing in the efavirenz arm [16].

The European Pregnancy and Paediatric Infections Cohort Collaboration (EPPICC) study reported a significant benefit for DTG vs protease inhibitors over 96 weeks (hazard ratio, 0.24; 95% CI, .16–.40) but no comparative data over the longer term. By 144 weeks, cumulative incidences of virologic failure (confirmed VL ≥400 c/mL) in EPPICC were 16.4% (95% CI, 8.5%–30.1%) among children initiating DTG who were ART naive and 21.1% (95% CI, 13.2%–32.7%) among children switching to DTG who were ART experienced and viremic [6]. These estimates are similar to the estimated risks of treatment failure on DTG in ODYSSEY, where risks were approximately 14% in participants who were ART naive (ODYSSEY A) and 20% in those who were ART experienced (ODYSSEY B). Notably, risks in EPPICC appeared somewhat lower than ODYSSEY at 96 weeks, although differences in study design and populations may explain this. In ODYSSEY, treatment failure was defined as either clinical or virologic, although confirmed VL ≥400 c/mL accounted for most failures on DTG (84%). Most clinical failures on DTG occurred early: 10 of 11 within the first 48 weeks of therapy. The EPPICC cohort included fewer young children among the ART naive participants (<6 years, 6%) as compared with ODYSSEY A (22%), while proportions of young children among participants who were ART experienced were similar (10% in EPPICC vs 7% in ODYSSEY B). In ODYSSEY, we saw a particularly high overall rate of treatment failure in the first 48 weeks in the <14-kg cohort (17% on DTG), who were mostly ODYSSEY A. Furthermore, VL was measured every 12 weeks in ODYSSEY during the randomized phase and likely measured less frequently on average in routine care in EPPICC, so virologic failures may have been detected earlier in ODYSSEY.

During extended follow-up, most children in the ODYSSEY SOC arm switched to DTG as countries transitioned to DTG-based treatment per national treatment guidelines. Similar to other studies [7, 8], we showed high rates of virologic suppression in children postswitch, with the highest rates in those children who were suppressed prior to switch.

Strengths of ODYSSEY include its broad eligibility, including children starting ART, children who were viremic and switched regimens, infants from 4 weeks of age to adolescents <18 years, and enrollment from 3 African countries, where most children with HIV reside, as well as Thailand and Europe. Other strengths include its excellent follow-up and minimal missing data. A limitation is the significant support for trial participants, which may limit generalizability of our findings to routine practice. In addition, the extended follow-up phase was observational in that clinicians were no longer asked to adhere to randomized treatment allocation, and VL monitoring and other laboratory measurements followed SOC practices, including no systematic resistance testing.

Treatment switching, predominantly to DTG in the SOC arm, was adjusted for appropriately, although >75% of SOC participants remained on SOC up to 192 weeks and the risk of failure in both trial arms was low between 192 and 240 weeks. Switching was also predominantly in response to national guidelines, and predictors of switch were not strong predictors of treatment outcome, meaning that maximum weights adjusting for treatment changes and censoring were moderate (all <2).

In conclusion, these data provide strong evidence that DTG-based regimens remain highly efficacious and durable for ∼4.5 years in children across the full pediatric age spectrum from 4 weeks of age and weighing at least 3 kg. These findings strongly support current WHO guidelines, which recommend DTG as the preferred first-line anchor drug and subsequent treatment option for children who have not previously received an INSTI [17].

## Supplementary Material

ofag266_Supplementary_Data

## Supplementary Data

Supplementary materials are available at *Open Forum Infectious Diseases* online. Consisting of data provided by the authors to benefit the reader, the posted materials are not copyedited and are the sole responsibility of the authors, so questions or comments should be addressed to the corresponding author.
