# Supplementary material for: Dolutegravir as First- or Second-line Treatment for Children With HIV: 240-Week Follow-up of the ODYSSEY Randomized Trial
Source: Open Forum Infect Dis. 2026 May 5;13(5):ofag266. doi: 10.1093/ofid/ofag266 (PMC13223059; doi:10.1093/ofid/ofag266)
Supplement: ofag266_Supplementary_Data [file ofag266_supplementary_data.pdf]

Supplementary Appendix

Title: Dolutegravir as first- or second-line treatment for children living with HIV: 240-week follow-up of the ODYSSEY randomised trial

Corresponding author: Deborah Ford; University College London Innovative Clinical Trials Unit (former MRC CTU at UCL), Institute of Clinical Trials and Methodology, London, United Kingdom; 90 High Holborn, London, United Kingdom, WC1V 6LJ; email: deborah.ford@ucl.ac.uk.

Contents page

ODYSSEY Trial Team 3

Supplementary Statistical Methods 6

Figure S1. ODYSSEY trial schema 9

Figure S2. ODYSSEY CONSORT diagram (randomised and extended follow-up) 10

Table S1. Baseline characteristics by weight cohort 12

Table S2. Baseline characteristics by ODYSSEY A/B 15

Figure S3. Time to switch off randomised allocation during randomised follow-up* 18

Figure S4. Time to switch off randomised allocation during trial (randomised* and extended$^{\alpha}$ follow-up) 20

Figure S5. Time to switch off randomised allocation or censoring during trial (randomised* and extended$^{\alpha}$ follow-up) 22

Table S3. On randomised allocation: Comparison of proportion with treatment failure by visit week 24

Table S4. Intention-to-treat: Comparison of proportion with treatment failure by visit week 26

Table S5. On randomised allocation: Comparison of proportion of participants with HIV-1 RNA <400 copies/mL by visit week 28

Table S6. On randomised allocation: Comparison of proportion of participants with HIV-1 RNA <50 copies/ml by visit week 30

Table S7. On randomised allocation: Comparison of proportion of participants with HIV-1 RNA <1000 copies/ml by visit week 32

Figure S6. On randomised allocation: Mean change in CD4 count from baseline 34

Figure S7. On randomised allocation: Mean change in CD4 percentage from baseline 36

Figure S8. Viral load<400c/mL after switching to DTG by pre-switch viral load in the SOC arm 38

Table S8. Intention-to-treat: safety endpoints comparing dolutegravir with standard-of-care to 192 weeks, by ODYSSEY A and B 39

Table S9. Intention-to-treat: safety endpoints comparing dolutegravir with standard-of-care to 144 weeks, by weight cohort 40

Table S10. Intention-to-treat: severe WHO 3 event, WHO 4 event or death events to 192 weeks 41

Table S11. Intention-to-treat: Serious adverse events to 192 weeks 43

Table S12. Intention-to-treat: Grade 3 or above clinical and laboratory adverse events to 192 weeks 48

Table S13. Intention-to-treat: Adverse events leading to ART modification (any grade) to 192 weeks 54

Table S14. Intention-to-treat: Neuropsychiatric adverse events to 192 weeks 56

Table S15. Intention-to-treat: Details neuropsychiatric adverse events to end of trial 57

ODYSSEY Trial Team

Penta Foundation: Davide Bilardi, Carlo Giaquinto, Daniel Gomez-Pena, Tiziana Grossele, Alessandra Nardone, Giulio Vecchia

Clinical Trial Units

UCL Innovative Clinical Trials Unit (former MRC CTU at UCL): Shabinah S. Ali, Abdel Babiker, Shazia Begum, Chiara Borg, Anne-Marie Borges Da Silva, Joanna Calvert, Man Chan, Nimisha Dudakia, Deborah Ford, Joshua Gasa, Diana M. Gibb, Lily Houlden, Khadija Ibrahim, Nasir Jamil, Sarah Lensen, Emma Little, Fatima Mohamed, Samuel Montero, Cecilia L. Moore, Rachel Oguntimehin, Anna Parker, Reena Patel, Tasmin Phillips, Tatiana Sarfati, Karen Scott, Clare Shakeshaft, Moira Spyer, Margaret Thomason, Anna Turkova, Rebecca Turner, Nadine Van Looy, Ellen White, Ian White, Kaja Widuch, Helen Wilkes, James Wyncoll, Ben Wynne

INSERM SC-10-US19—ANRS: Alexandra Compagnucci, Yacine Saidi, Yoann Riault, Alexandra Coelho, Laura Picault, Christelle Kouakam

AMS-PHPT Research Collaboration, Chiang Mai University: Tim R. Cressey, Suwalai Chalermpantmetagul, Dujrudee Chinwong, Gonzague Jourdain, Rukchanok Peongjakta, Praornsuda Sukrakanchana, Wasna Sirirungsi

Trial sites

Joint Clinical Research Centre, Uganda: Cissy M. Kityo, Victor Musiime, Elizabeth Kaudha, Annet Nanduudu, Emmanuel Mujyambere, Paul Ocitti Labeja, Charity Nankunda, Juliet Ategeka, Peter Erim, Collin Makanga, Esther Nambi, Abbas Lugemwa, Lorna Atwine, Edridah Keminyeto, Deogratiuos Tukwasibwe, Shafic Makumbi, Emily Ninsiima, Mercy Tukamushaba, Rogers Ankunda, Ian Natuhurira, Miriam Kasozi, Baker Rubinga, Diana Antonia Rutebarika, Rashidah Nazzinda, Shamim Nakabuye, Julius Tumusiime, Alice Mulindwa, Ritah Mbabazi, Milly Ndigendawani, Edward Bagirigomwa, Eddie Rubanga, David Eram, Maria Nannungi, Chrispus Katemba, Disan Mulima, Josephine Namusanje, Priscilla Kyobutungi, Phyllis Mwesigwa Rubondo, Robinah Kibenge, Claire Nasaazi, Basiimwa Roy Clark, Enock Babu, Alex Musiime, Faith Mbasani, Martin Ojok, Odoch Denis, David Baliruno, Katabalwa Juliet, Benson Ouma, Barbara Ainebyoona, Mariam Naabalamba, Diana A. Rutebarika, Alex V. Musiime, Josephine Kobusungye, Ezra Lutalo, Sharif Musumba

Baylor College of Medicine Children's Foundation, Uganda: Adeodata R. Kekitiinwa, Pauline Amuge, Dickson Bbuye, Justine Nalubwama, Winnie Akobye, Muzamil Nsibuka Kisekka, Anthony Kirabira, Gloria Ninsiima, Sylvia Namanda, Gerald M. Agaba, Immaculate Nagawa, Annet Nalugo, Florence Namuli, Rose J. Kadhuba, Rachael K. Namuddu, Lameck Kiyimba, Angella Baita, Eunice Atim, Olivia Kobusingye, Clementine Namajja, Africanus Byaruhanga, Rogers Besigye, Herbert Murungi, Geoffrey Onen, Lawrence Lekku, Judith Tikabibamu, Henry Balwa

MUJHU Research Collaboration, Uganda: Philippa Musoke, Linda Barlow-Mosha, Grace Ahimbisibwe, Rosemary Namwanje, Hajira Kataike, Mark Ssenyonga, Brenda Kakayi, Rebecca Sakwa, Sarah Nakabuye, Barbara Musoke Nakirya, Gladys Kasangaki, Raymonds Kyambadde, David Balamusani, Winnie Nansamba, Stella Nalusiba, Emmanuel Mayanja, Richard Isabirye, Erinah Kyomukama, Rebecca Wampamba, Mildred Kabasonga, Zaam Zinda Nakawungu, Sarah Babirye, Olivia Kaboggoza, Juliet Nanyonjo, Joanita Nankya Baddokwaya, Alice Elwana, Winfred Kaahwa, Bosco Kafufu, Emmanuel Hakiza, Maria Musisi, Paula Namayanja, Maria Gorreti Nakalema, Robert Serunjogi, Monica Etima, Phionah Kibalama, Joel Maena, Agnes Mary Mugagga, Annet Miwanda, Monica Nolan.

UZCRC, Zimbabwe: James Hakim, Hilda Mujuru, Kusum Nathoo, Mutsa Bwakura-Dangarembizi, Ennie Chidziva, Shepherd Mudzingwa, Themelihle Bafana, Secrecy Gondo, Colin Warambwa, Godfrey Musoro, Vivian Mumbiro, Gloria Tinago, Shirley Mutsai, Joy Chimanzi, Columbus Moyo, Ruth Nhema, Misheck Nkalo Phiri, Stuart Chitongo, Joshua Choga, Joyline Bhiri, Wilber Ishemunyoro, Makhosonke Ndlovu, Moses Chitsamatanga, Pia Ngwaru, Tsitsi Gwenzi, Wendy Mapfumo, Dorothy

Murungu, Trust Mukanganiki, Prosper Dube, Tapiwa Gwaze, Farai Matimba, Tawona Mudzviti, Zivai Mupambireyi, Sibusisiwe Weza, Cleopatra Langa, Sandra Musarurwa, Shamiso Gwande

FAM-CRU, South Africa: Mark F. Cotton, Anita Janse van Rensburg, Marlize Smuts, Catherine Andrea, Sumaya Dadan, Sonja Pieterse, Vinesh Jeaven, Candice Makola, George Fourie, Kurt Smith, Els Dobbels, Peter Zuidewind, Hesti Van Huyssteen, Mornay Isaacs, Georgina Nentsa, Thabisa Ncgaba, Candice MacDonald, Maria Bester, Wilma Orange, Ronelle Arendze, Mark Mulder, Lucille Malgraaf, Ashley Harley, Yasmeen Akhalwaya, Nontuthuzelo Daizana, Andile Cweya

PHRU, South Africa: Avy Violari, Nastassja Ramsagar, Afaaf Liberty, Ruth Mathiba, Mandisa Nyati, Haseena Cassim, Lindiwe Maseko, Nkata Kekane, Busi Khumalo, Mirriam Khunene, Noshalaza Sbisi, Jackie Brown, Tryphina Madonsela, Nokuthula Mbadaliga, Zaakirah Essack, Reshma Lakha, Aasia Vadee, Derusha Frank, Nazim Akoojee, Maletsatsi Monametsi, Gladness Machache, Yolandie Fourie, Anusha Nanan-kanjee, Juan Erasmus, Angelous Mamiane, Tseleng Daniel, Fatima Mayat, Nomfundo Maduna, Patsy Baliram, Sibongile Sithebe, Emily Lebotsa, Siphiwe Mkhize

Klerksdorp Tshepong Hospital Complex, South Africa: Ebrahim Variava, Modiehi Rakgokong, Dihedile Scheppers, Tumelo Moloantoa, Abdul Hamid Kaka, Tshepiso Masienyane, Akshmi Ori, Kgosimang Mmolawa, Pattamukkil Abraham.

Durban International Clinical Research Site, South Africa: Moherndran Archary, Rosie Mngqibisa, Rejoice Mosia, Sajeeda Mawlana, Rashina Nundlal, Elishka Singh, Penelope Madlala, Allemah Naidoo, Sphiwee Cebekhulu, Petronelle Casey, Collin Pillay, Subashinie Sidhoo, Minenhle Chikowore, Lungile Nyantsa, Melisha Nunkoo, Terence Nair, Enbavani Pillay, Sheroma Rajkumar, Sheleika Singh

AHRI, South Africa: Nigel Klein, Osee Behuhuma, Olivier Koole, Kristien Bird, Nomzamo Buthelezi, Mumsy Mthethwa, Gugu Gasa, Siva Danaviah and Theresa Smit

AMS-PHPT CTU, Chiang Mai University, Thailand: Tim R. Cressey, Suwalai Chalermpantmetagul, Gonzague Jourdain, Nicole Ngo Giang Huong, Dujrudee Chinwong, Chalermpong Saenjum, Rukchanok Peongjakta, Pra-ornsuda Sukrakanchana, Woottichai Khamduang, Laddawan Laomanit, Ampika Kaewbundit, Jiraporn Khamkon, Kanchana Than-in-at, Sanuphong Chailert, Worathip Sripaoraya, Nitinart Krueduangkam, Namthip Kruenual, Warunee Khamjakkaew, Soraya Klinprung, **Prapokklao Hospital:** Chaiwat Ngampiyaskul, Pisut Greetanukroh, Praechadaporn Khannak, Pathanee Tearsansern, Wanna Chamjamrat, **Phayao Hospital, Thailand:** Nuttawut Chanto, Thitiwat Thapwai, Khanungnit Thungkham, Patcharee Puangmalai, Chutima Ruklao, **Chiangrai Prachanukroh Hospital, Thailand:** Pradthana Ounchanum, Suwimon Khusuwan, Sukanda Denjanta, Yupawan Thaweesombat, Jutarat Thewsoongnoen, Kanyanee Kaewmamueng, Phakamas Kamboua, Supawadee Pongprapass (Sangjan), Warunee Srisuk, Areerat Kongponoi, Juthamas Limplertjareanwanich, **Nakornping Hospital, Thailand:** Suparat Kanjanavanit, Prattana Leenasirimakul, Chayakorn Saewtrakool, Pacharaporn Yingyong, Duangrat Chutima (Suwan), Raungwit Junkaew, Orapin Khatngam, Thannapat Chankun, **Khon Kaen Hospital, Thailand:** Ussanee Srirompotong, Patamawadee Sudsaard, Sookpanee Wimonklang, Turian Petpranee, **Mahasarakam Hospital, Thailand:** Sathaporn Na-Rajsima, Pattira Runarassamee, Nuananong Kunjaroenrut, Arttasid Udomvised, Tassawan Khayanchoomnoom, Watchara Meethaisong, Ketmookda Trairat
HIVNAT, Thailand: Thanyawee Puthanakit, Suvaporn Anugulruengkitt, Wipaporn Natalie Songtaweesin, Torsak Bunupuradah, Naruporn Kasipong, Sararut Chanthaburanun, Apicha Mahanontharit, Kesdao Nanthapisal, Thidarat Jupimai, Thornthun Noppakaorattanamanee, Chutima Saisaengjan

European Site Investigators: Goethe University Frankfurt, Germany: Stephan Schultze-Strasser, Christoph Königs , **UKE Eppendorf, Germany:** Robin Kobbe, Ulf Schulze-Sturm, Felicia Mantkowski, Cornelius Rau, **Heartlands Hospital, UK:** Steve Welch, Jacqui Daglish, Laura Thrasyvoulou, Kate Gandhi, Yvonne Vaughan-Gordon, **Great Ormand Street Hospital, UK:** Delane Shingadia, Sophie Foxall, Judith Acero, Malgorzata Pasko-Szcech, Jacquie Flynn, **St Mary's Hospital, UK:** Gareth Tudor-Williams, Amina Farhana Mehar, Caroline Foster, Sobia Mustafa, **Leicester Royal Infirmary, UK:** Srini Bandi, Jin Li, Jackie Philps, **Leeds General Infirmary, UK:** Sean O'Riordan, Dominique Barker, Richard Vowden, Maria Dowie **Kings College Hospital, UK:** Colin Ball, Eniola Nsirim, Kathleen McClaughlin, **Hospital 12 de Octubre, Spain:** India Garcia, Pablo Rojo Conejo, Cristina Epalza, Luis Prieto Tato, Maite Fernandez **Hospital La Paz, Spain:** Luis Escosa Garcia, Maria José Mellado Peña, Talia Sainz Costa, **Hospital San Joan de Déu, Spain:** Claudia Fortuny Guasch, Antoni Noguera Julian, Carolina Estepa, Elena Bruno, Patricia Mendez Garcia, Alba Murciano Cabeza, Biobanco Gregorio Maranon, Maria Angeles Muñoz Fernandez, Jose Luis Jimenez, Coral Gomez Rico, **Centro**

Materno-infantile do Norte, Portugal: Laura Marques, Carla Teixeira, Alexandre Fernandes, Rosita Nunes, Helena Nascimento, Andreia Padrao, Joana Tuna, Helena Ramos, Ana Constança Mendes, Helena Pinheiro, Ana Cristina Matos

Local Site Monitors: Flavia Kyomuhendo, Sarah Nakalanzi, Cynthia Mukisa Williams, Leora Sewnarain, Ntombenhle Ngcobo, Deborah Pako, Nompumelelo Yende, Jacky Crisp, Marlize Smuts, Benedictor Dube, Precious Chandiwana, Winnie Gozhora, Thidarat Jumpimai, Petronilla Matibe

Substudies

PK substudies: David Burger, Pauline Bollen, Angela Colbers, Hylke Waalewijn, Tom Jacobs

Virology-immunology substudy: Nigel Klein, Eleni Nastouli, Anita De Rossi, Maria Angeles Munoz Fernandez, Carlota Miranda, Moira Spyer, Kathleen Gartner

Social Science substudy and Youth Trial Board project: Janet Seeley, Sarah Bernays, Stella Namukwaya, Zivai Mupambireyi, Magda Conway, Lungile Jafta, Mercy Shibemba

Trial Committees

Independent Trial Steering Committee Members: Ian Weller, Elaine Abrams, Tsitsi Apollo, Polly Clayden, Valériane Leroy

Independent Data Monitoring Committee Members: Anton Pozniak, Jane Crawley, Rodolphe Thiébaut, Helen McIlleron

Endpoint Review Committee Members: Alasdair Bamford, Hermione Lyall, Andrew Prendergast, Felicity Fitzgerald, Anna Goodman

Supplementary Statistical Methods

Analyses were conducted in the total trial population including participants enrolled in ≥14kg and <14kg cohorts and including those starting first-line and second-line. Follow-up was censored at 01 May 2023, or last outcome measurement, whichever occurred earlier.

For the primary endpoint, the earliest date of clinical or virological failure was used as date of treatment failure. Treatment failure was defined as the first occurrence of any of the following: a decrease of less than 1 log10 in the viral load at week 24 (or a viral load of ≥50 c/mL at week 24 if the viral load had been <500c/mL at baseline) and a switch to second- or third-line ART for treatment failure; virologic failure (defined as two consecutive viral load results of ≥400c/mL, the first occurring at or after week 36); a new or recurrent acquired immunodeficiency syndrome (AIDS)–defining event (WHO stage 4) or severe WHO stage 3 event; or death from any cause. Where two consecutive HIV RNA are ≥400c/ml, the date of the next scheduled visit following the first HIV RNA ≥400 (using schedule from randomisation) was used to calculate the time the first confirmed viral load measurement ≥400c/ml (treatment failure date), rather than the date of the confirmatory measure.

Date of treatment failure was assigned to the visit window, in which it occurred; windows were -42 days to + 41 days around the target visit date, except for week 4 where the window -27 days to + 27 days was used, and week 12 where the window -28 to +41 days was used. Analysis of 48-weekly cross-sectional viral load results and CD4/CD4 were similarly assigned to visit windows, the closest measurement to the target date was used as the outcome measure for the window (using the later measurement if 2 were equidistant from the target date), with outcome set to missing where the measurement was not at a 48-weekly timepoint. Outcomes were not included for scheduled visits once the number of participants on DTG or SOC had dropped <10.

Efficacy analyses estimate the effects of being prescribed DTG vs. alternative non-DTG-based ART, i.e. effects assuming participants remained "on randomised allocation". Treatment regimens of interest were defined as DTG-based ART for a participant assigned to the DTG arm and non-DTG-based ART for a participant assigned to the SOC arm. Participants were artificially censored after they switched from their randomised allocation. Inverse-probability weights were used to adjust for artificial censoring and weights to account for censoring due to death (except for analyses of treatment failure where death is included in the composite endpoint), loss to follow-up, or administrative censoring (due to exit at end of randomised phase) were also applied. No adjustment was made for exit due to end of trial follow-up; differences in length of follow-up were adjusted for directly in the outcome models. Weights adjusted for most recent weight, BMI-for-age, CD4 count (CD4% for <14kg cohort) and $\log_{10}$ viral load. Median imputation was used to account for missing baseline CD4 (4 participants) and viral load (5 participants); imputed values were used for covariate adjustment in all analyses and estimation of censoring weights, except for change from baseline analyses (CD4 and CD4%), where participants with missing baseline data were considered missing.

Marginal probabilities of treatment failure by 240 and absolute differences between DTG and SOC in the trial population were estimated using flexible parametric survival models. Risk of treatment failure by 48, 96, 144, and 192 weeks were estimated using data to each of the 48-weekly timepoint separately. Models were fitted on the log cumulative hazard scale using restricted cubic splines (3 knot cubic spline – knots at 25th, 50th and 75th percentiles of event times).[1, 2] Knots were specified according to cohort (i.e. total population, ODYSSEY A/B, ≥14kg/<14kg cohort) and time-point being analysed, with fewer knots used where duplicate knot positions occurred. We adjusted for baseline covariates including ODYSSEY A/B, country or region (Uganda, South Africa, Zimbabwe, and Thailand or Europe) calendar month of randomisation (3 knot cubic spline - knots at 10th, 50th, 90th

percentiles, specified according to cohort), sex, age, weight, BMI-for-age Z-score, CD4 (CD4% for <14kg cohort), and $\log_{10}$ viral load.

Mean changes in CD4/CD4% from baseline up to 240 weeks, and differences between DTG and SOC, were estimated using linear mixed models. Models included a random intercept for participant and fixed effects for trial arm (equivalent to treatment regimen in analyses on randomised allocation due to censoring at switch) and visit week (3 knot cubic spline - knots at 10th, 50th, 90th percentiles, specified according to cohort), with interaction terms between trial arm and visit week. We adjusted for baseline covariates including ODYSSEY A/B, country or region (Uganda, South Africa, Zimbabwe, Thailand and Europe) calendar month of randomisation (3 knot cubic spline - knots at 10th, 50th, 90th percentiles, specified according to cohort), sex, age, weight, BMI-for-age Z-score, and $\log_{10}$ viral load. Models also included the outcome measure at baseline and interaction terms between the outcome measure at baseline [as categorical, with category intervals defined at 25th, 50th, 75th percentiles] and visit week spline variables, to best model the outcome trajectory over time.[3]

Proportions of participants with viral load <50, <400 and <1000c/mL at week 48, 96, 144, 192 and 240 weeks were estimated from longitudinal logistic mixed models across all timepoints. Models included a random intercept for participants and fixed effects for trial arm and visit week (3 knot cubic spline - knots at 10th, 50th, 90th percentiles, specified according to cohort), including interaction between trial arm and visit week spline variables, and other baseline variables: ODYSSEY A or B, country or region (Uganda, South Africa, Zimbabwe, and Thailand or Europe), calendar month of randomisation (3 knot cubic spline - knots at 10th, 50th, 90th percentiles, specified according to cohort), sex, age, weight, BMI-for-age Z-score, CD4 (CD4% for <14kg cohort), and log10 viral load.

Analyses on randomised allocation are reported to week 240, except where we report results separately for the <14kg cohort when we report to week 192. Sub-group differences (ODYSSEY A vs B; ≥14kg vs <14kg) in the effect of ART-regimen on the risk of treatment failure were estimated using data to each of the 48-weekly timepoints. For other efficacy outcomes (cross-sectional VL, CD4, CD4%) heterogeneity in treatment effects were estimated from longitudinal mixed models (logistic and linear, respectively) incorporating all time-points.

Risk of treatment failure were also estimated in the intention-to-treat (ITT) population to week 192, and up to week 144 where report <14kg cohort separately. No adjustment was made for loss to follow-up or administrative censoring.

Cox models were used to compare time to first adverse event between treatment arms, in the ITT population, up to 192 weeks. Models adjusted for ODYSSEY A/B in the total population and ≥14kg cohort analyses. Events up to 144 weeks were reported where report <14kg cohort separately.

The probability of treatment switch was modelled to obtain IPW (switch model). Similarly, the probability of censoring was modelled to obtain IPCW (censoring model). Then, the association between treatment regime and outcome was estimated in a regression model (weighted by IPW and IPCW), censoring follow-up from the earlier of the interval after treatment switch or the interval in which the participant was censored. If a participant switched treatment regime prior to the outcome CD4/CD4% or cross-sectional viral load in the same interval, the closest measurement taken up to and including date of switch was used for the interval; this scenario was rare as the majority of treatment switches were at a scheduled visit. If censoring was due to loss follow-up or administrative (participant not participating in extended follow-up), participant follow-up was censored in the

interval following the participant's last attended scheduled visit. If censoring was due to death (except for analysis of treatment failure), participant follow-up was censored in the same interval as the death.

The probability of switch from allocated treatment regime, A(k), where A(k)=1 indicates switch before the end of visit window k, was estimated using pooled logistic regression:

$$\text{logit}(P[A(k) = 1 | \bar{A}(k\text{-}1) = 0 , C(k) = 0, V, \bar{L}(k-1)]) = \alpha_0 (w_k) + \alpha_1' V + \alpha_2' \bar{L}(k-1)$$

for k=1, 2, 3 … corresponding to visit weeks w_k = 4, 12, 24, … with visit intervals as defined above. $\alpha_0(w_k)$ is a visit-week specific intercept (modelled by a restricted cubic spline with knots at 10th, 50th, 90th percentiles, specified according to weight cohort). $\bar{A}(k-1)$ = 0 indicates on allocated treatment up to and including the end of visit interval k-1, with A (0) = 1 as all participants were on allocated treatment at baseline. C(k)=0 if an individual remains uncensored to the end of visit window k (as defined above). V includes baseline factors at trial enrolment and $\bar{L}(k-1)$ is history of time-dependent confounders up to the end of interval k-1. Separate models were fitted for each trial arm (DTG and SOC), weight cohort (≥14kg and <14kg cohorts), and ODYSSEY A and B (≥14kg cohort only).

Equivalent models were fitted to estimate the probability of change from allocated treatment regime using baseline covariates only. The stabilised inverse probability weight IPW (t-1) for remaining on the allocated treatment regime to the end of the previous interval was then used to weight the observation in interval, and was estimated as follows:

$$IPW(t-1) = \frac{\prod_{k=1}^{t-1} P[A(k) = 0 | \bar{A}(k\text{-}1) = 0 , C(k) = 0, V]}{\prod_{k=1}^{t-1} P[A(k) = 0 | \bar{A}(k\text{-}1) = 0 , C(k) = 0, V, \bar{L}(k-1)]}$$

Baseline variables (V) included were country or region (Uganda, South Africa, Zimbabwe, Thailand, Europe), calendar month of randomisation (modelled by a restricted cubic spline with knots at 10th, 50th, 90th percentiles, specified according to weight cohort), sex, baseline age, weight (linear), BMI-for-age Z score (linear), CD4 (linear) (CD4% (linear) for <14kg cohort) and $\log_{10}$ viral load (linear). Time-dependent variables (L) included were most recent weight (linear), BMI-for-age Z score (linear), CD4 (linear) (CD4% (linear) for <14kg cohort) and $\log_{10}$ viral load (linear). The last measurement in the visit window was used to predict treatment switch in the next interval, with last observation carried forward, where there was no observation in the visit window.

The probability of censoring due to due to death (except for analysis of treatment failure), loss to follow-up, or administrative censoring where C(k)=1 if an individual is censored in interval k (as defined above), was estimated in for each treatment arm using pooled logistic regression:

$$\text{logit}(P[C(k) = 1 | \bar{A}(k\text{-}1) = 0 , C(k-1) = 0, V, \bar{L}(k-1)]) = \beta_0 (w_k) + \beta_1' V + \beta_2' \bar{L}(k-1)$$

Separate models were fitted for each trial arm (DTG and SOC) and weight cohort (≥14kg and <14kg cohorts). The same baseline (plus adjustment for ODYSSEY A or B, but without country/region) and time-dependent factors were used as in the switch model. Stabilised censoring weights (IPCW, to upweight those who remained in follow-up in the same way as we upweighted those who remained on randomised allocation) were estimated and the product IPW(t-1)*IPCW(t) was used to weight the outcome models.

Figure S1. ODYSSEY trial schema

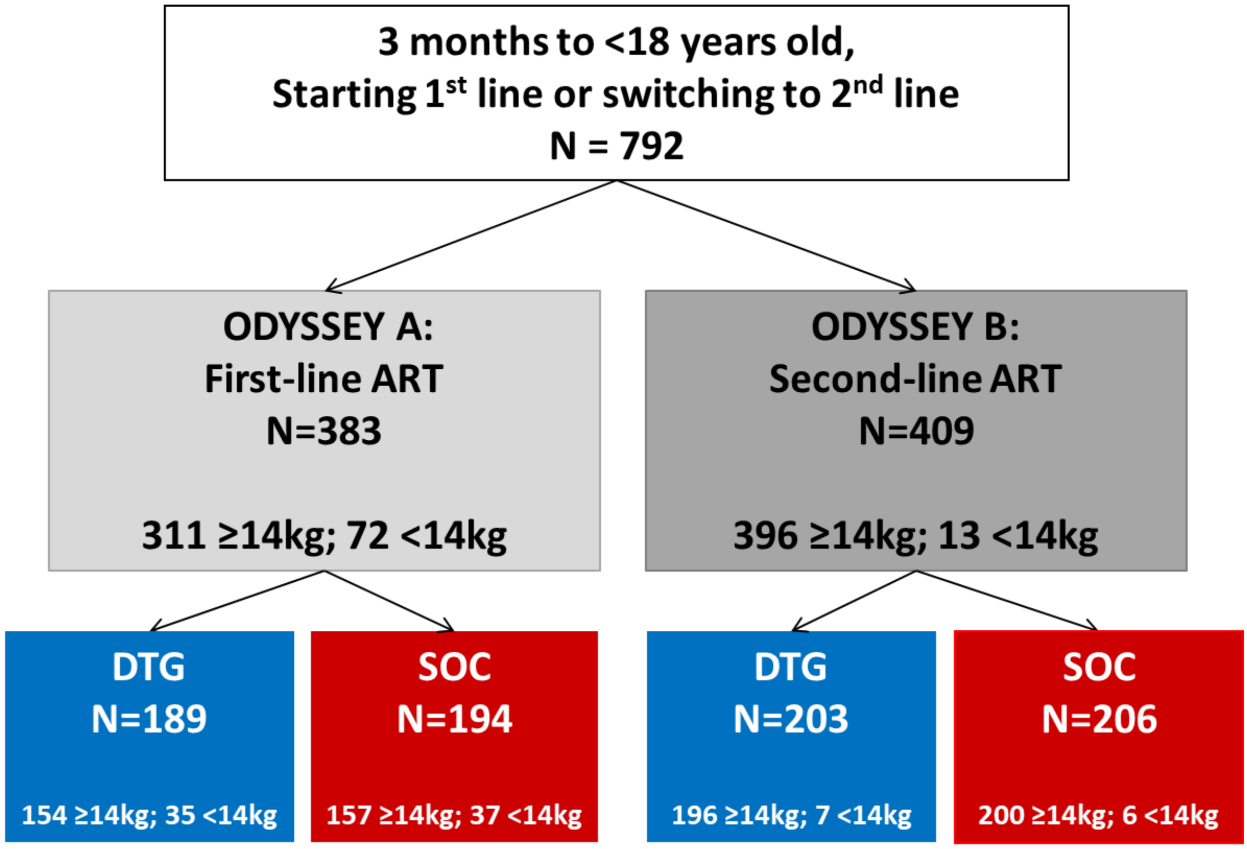

DTG=dolutegravir. SOC=standard of care.

Figure S2. ODYSSEY CONSORT diagram (randomised and extended follow-up)

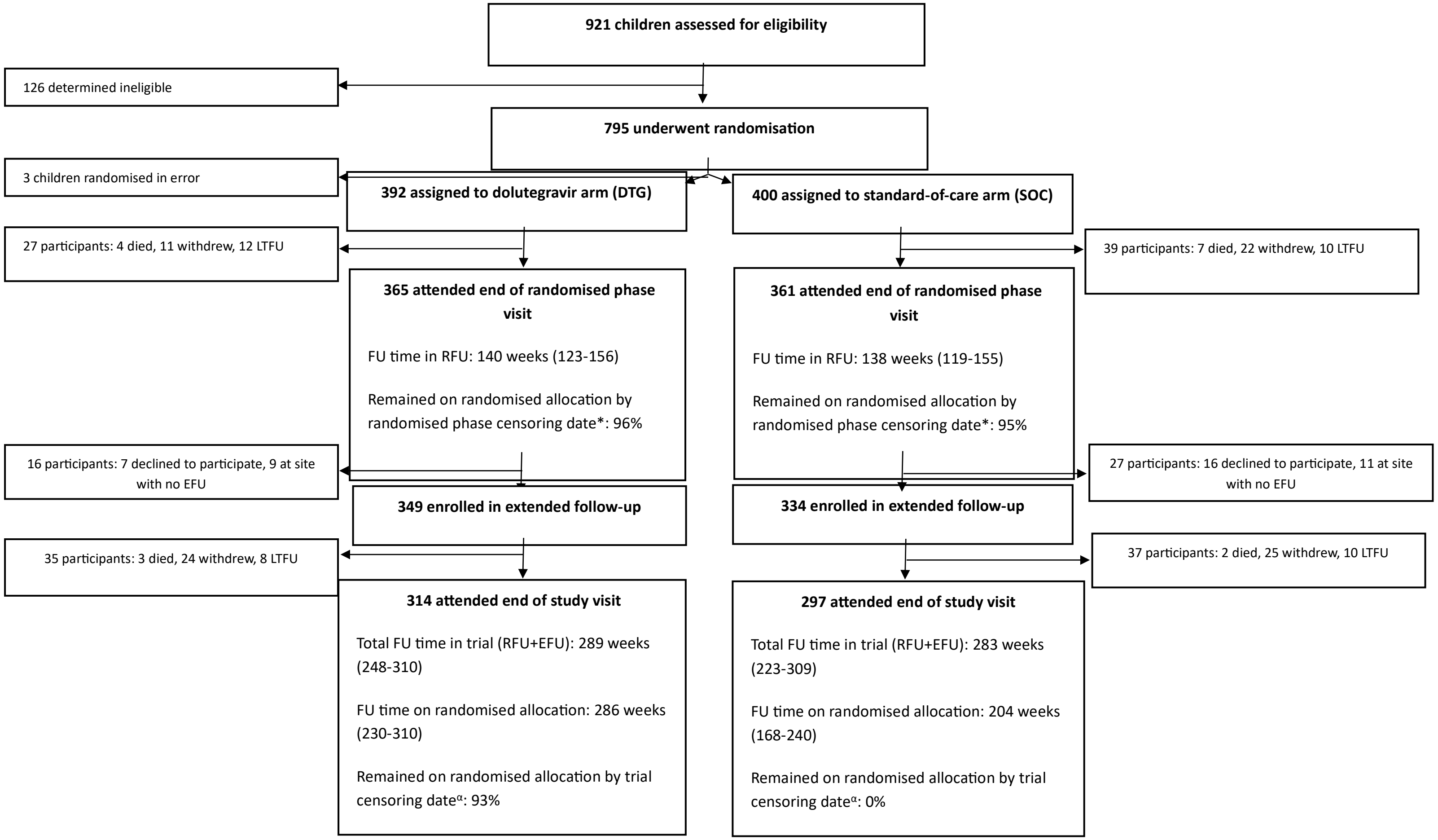

** Randomised follow-up was until the last participant reached week 96 (≥14kg cohort, 24 April 2020; <14kg cohort, 28 June 2021). Proportions remaining on randomised allocation are Kaplan-Meier estimates (Figure S3).*
α Extended follow-up continued until 01 May 2023, when the last participant in the ≥14kg cohort reached 240 weeks and the last participant in the <14kg cohort reached 192 weeks. Proportions remaining on randomised allocation are Kaplan-Meier estimates (Figure S4).

Abbreviations: DTG=dolutegravir. EFU=extended follow-up. FU= follow-up. LTFU=lost-to-follow-up. RFU=randomised follow-up. SOC=standard-of-care.

Table S1. Baseline characteristics by weight cohort

	≥14kg			<14kg		
	**DTG**	**SOC**	**Total**	**DTG**	**SOC**	**Total**
Participants randomised	350	357	707	42	43	85
**ODYSSEY A/B**						
A	154 (44%)	157 (44%)	311 (44%)	35 (83%)	37 (86%)	72 (85%)
B	196 (56%)	200 (56%)	396 (56%)	7 (17%)	6 (14%)	13 (15%)
**Weight cohort**						
>=14kg	350 (100%)	357 (100%)	707 (100%)	0 (0%)	0 (0%)	0 (0%)
<14kg	0 (0%)	0 (0%)	0 (0%)	42 (100%)	43 (100%)	85 (100%)
**Country/region of residence**						
Europe	12 (3%)	13 (4%)	25 (4%)	0 (0%)	0 (0%)	0 (0%)
South Africa	61 (17%)	83 (23%)	144 (20%)	8 (19%)	12 (28%)	20 (24%)
Thailand	28 (8%)	33 (9%)	61 (9%)	0 (0%)	0 (0%)	0 (0%)
Uganda	170 (49%)	161 (45%)	331 (47%)	22 (52%)	21 (49%)	43 (51%)
Zimbabwe	79 (23%)	67 (19%)	146 (21%)	12 (29%)	10 (23%)	22 (26%)
**Sex**						
male	176 (50%)	186 (52%)	362 (51%)	16 (38%)	25 (58%)	41 (48%)
female	174 (50%)	171 (48%)	345 (49%)	26 (62%)	18 (42%)	44 (52%)
**Age (years)**						
median	12.2	12.1	12.2	1.3	1.5	1.4
[IQR]	[9.2, 15.1]	[8.8, 14.7]	[9.1, 14.9]	[0.5, 2.0]	[0.6, 2.1]	[0.6, 2.0]
[range]	[3.4-18.0]	[2.9-18.0]	[2.9-18.0]	[0.3-5.9]	[0.1-4.5]	[0.1-5.9]
**Weight (kg)**						
median	30.4	31.0	30.7	8.1	8.2	8.1
[IQR]	[23.7, 43.7]	[23.3, 42.7]	[23.4, 43.0]	[5.6, 10.0]	[5.2, 10.3]	[5.4, 10.0]
[range]	[14.0-85.0]	[14.2-72.7]	[14.0-85.0]	[3.8-13.0]	[3.4-13.4]	[3.4-13.4]
**CD4 lymphocyte percentage**						
n	350	357	707	41	40	81
median	20	22	21	24	23	23
[IQR]	[10, 29]	[13, 31]	[12, 30]	[17, 34]	[14, 30]	[16, 31]
[range]	[0.0-59.0]	[0.5-69.5]	[0.0-69.5]	[3.0-46.0]	[1.0-52.5]	[1.0-52.5]
<15	121 (35%)	108 (30%)	229 (32%)	7 (17%)	11 (28%)	18 (22%)
15-<30	152 (43%)	147 (41%)	299 (42%)	22 (54%)	18 (45%)	40 (49%)
>=30	77 (22%)	102 (29%)	179 (25%)	12 (29%)	11 (28%)	23 (28%)

missing	0	0	0	1	3	4
**CD4 lymphocyte count (cells/mm3)**						
n	350	357	707	41	40	81
median	444	486	459	1639	1221	1391
[IQR]	[196, 652]	[254, 751]	[228, 707]	[1026, 2327]	[633, 1870]	[863, 2060]
[range]	[3.5-2445.5]	[2.0-2494.0]	[2.0-2494.0]	[67.0-4675.5]	[54.0-5299.5]	[54.0-5299.5]
<50	40 (11%)	38 (11%)	78 (11%)	0 (0%)	0 (0%)	0 (0%)
50-<100	21 (6%)	10 (3%)	31 (4%)	1 (2%)	4 (10%)	5 (6%)
100-<200	27 (8%)	22 (6%)	49 (7%)	2 (5%)	0 (0%)	2 (2%)
200-<350	47 (13%)	55 (15%)	102 (14%)	1 (2%)	0 (0%)	1 (1%)
350-<500	71 (20%)	59 (17%)	130 (18%)	1 (2%)	3 (8%)	4 (5%)
500-<1000	104 (30%)	124 (35%)	228 (32%)	5 (12%)	7 (18%)	12 (15%)
1000-1500	32 (9%)	42 (12%)	74 (10%)	8 (20%)	11 (28%)	19 (23%)
>=1500	8 (2%)	7 (2%)	15 (2%)	23 (56%)	15 (38%)	38 (47%)
missing	0	0	0	1	3	4
**Viral load (copies/mL)**						
<400	5 (1%)	10 (3%)	15 (2%)	0 (0%)	1 (3%)	1 (1%)
400-<1,000	11 (3%)	6 (2%)	17 (2%)	2 (5%)	2 (5%)	4 (5%)
1,000-<10,000	77 (22%)	107 (30%)	184 (26%)	2 (5%)	4 (10%)	6 (7%)
10,000-<50,000	115 (33%)	113 (32%)	228 (32%)	9 (21%)	2 (5%)	11 (14%)
50,000-<100,000	44 (13%)	45 (13%)	89 (13%)	4 (10%)	3 (8%)	7 (9%)
100,000-<500,000	82 (23%)	61 (17%)	143 (20%)	12 (29%)	15 (38%)	27 (33%)
500,000-<1,000,000	10 (3%)	10 (3%)	20 (3%)	5 (12%)	6 (15%)	11 (14%)
>=1,000,000	6 (2%)	4 (1%)	10 (1%)	8 (19%)	6 (15%)	14 (17%)
missing	0	1	1	0	4	4
**Log10 Viral load (log10 copies/mL)**						
n	350	356	706	42	39	81
median	4.5	4.4	4.4	5.2	5.5	5.3
[IQR]	[3.9, 5.1]	[3.7, 4.9]	[3.9, 5.0]	[4.4, 5.8]	[4.8, 5.9]	[4.6, 5.9]
[range]	[1.3-6.6]	[1.3-6.5]	[1.3-6.6]	[2.7-6.6]	[2.1-7.0]	[2.1-7.0]
**History of WHO staging**						
stage1	124 (35%)	146 (41%)	270 (38%)	20 (48%)	16 (37%)	36 (42%)
stage2	129 (37%)	119 (33%)	248 (35%)	11 (26%)	9 (21%)	20 (24%)
stage3	69 (20%)	60 (17%)	129 (18%)	6 (14%)	8 (19%)	14 (16%)
stage4	28 (8%)	32 (9%)	60 (8%)	5 (12%)	10 (23%)	15 (18%)
**NRTI backbone at randomisation**						

ABC 3TC	232	(66%)	231	(65%)	463	(65%)	38	(90%)	37	(86%)	75	(88%)
ABC TDF	1	(0%)	2	(1%)	3	(0%)	0	(0%)	0	(0%)	0	(0%)
TDF/TAF 3TC/FTC	80	(23%)	84	(24%)	164	(23%)	0	(0%)	0	(0%)	0	(0%)
ZDV 3TC	37	(11%)	40	(11%)	77	(11%)	4	(10%)	6	(14%)	10	(12%)
**Anchor drug class at randomisation**												
INSTI	350	(100%)	1	(0%)	351	(50%)	42	(100%)	2	(5%)	44	(52%)
NNRTI	0	(0%)	154	(43%)	154	(22%)	0	(0%)	9	(21%)	9	(11%)
PI	0	(0%)	202	(57%)	202	(29%)	0	(0%)	32	(74%)	32	(38%)
**Anchor drug at randomisation**												
ATV	0	(0%)	49	(14%)	49	(7%)	0	(0%)	0	(0%)	0	(0%)
DRV	0	(0%)	6	(2%)	6	(1%)	0	(0%)	0	(0%)	0	(0%)
DTG	350	(100%)	0	(0%)	350	(50%)	42	(100%)	0	(0%)	42	(49%)
EFV	0	(0%)	150	(42%)	150	(21%)	0	(0%)	4	(9%)	4	(5%)
EVG	0	(0%)	1	(0%)	1	(0%)	0	(0%)	0	(0%)	0	(0%)
LOP	0	(0%)	147	(41%)	147	(21%)	0	(0%)	32	(74%)	32	(38%)
NVP	0	(0%)	2	(1%)	2	(0%)	0	(0%)	5	(12%)	5	(6%)
RAL	0	(0%)	0	(0%)	0	(0%)	0	(0%)	2	(5%)	2	(2%)
RLP	0	(0%)	2	(1%)	2	(0%)	0	(0%)	0	(0%)	0	(0%)

Data are n(%), median [IQR], or median [IQR; range].

§ At a participant level, the mean of the measured values was used if measured values were available at screening and randomisation.

‡ Worst known stage prior to enrolment.

¶ Two ≥14kg cohort participants in the SOC group initiated tenofovir alafenamide and emtricitabine (one in ODYSSEY A and one in ODYSSEY B).

Abbreviations: 3TC=lamivudine. ABC=abacavir. ATV=atazanavir. DRV=darunavir. DTG=dolutegravir. EFV=efavirenz. EVG=elvitegravir. FTC=emtricitabine. INSTI=integrase inhibitor. IQR=interquartile range. LPV=lopinavir. NRTI=nucleoside reverse transcriptase inhibitors. NNRTI=non-nucleoside reverse transcriptase inhibitors. NVP=nevirapine. PI=protease inhibitor. RAL=raltegravir. RLP=rilpivirine. SOC=standard of care. TAF=tenofovir alafenamide. TDF=tenofovir disoproxil fumarate. WHO= World Health Organization. ZDV=zidovudine.

Table S2. Baseline characteristics by ODYSSEY A/B

	ODYSSEY A						ODYSSEY B					
	DTG		SOC		Total		DTG		SOC		Total	
Participants randomised	189		194		383		203		206		409	
**ODYSSEY A/B**												
A	189	(100%)	194	(100%)	383	(100%)	0	(0%)	0	(0%)	0	(0%)
B	0	(0%)	0	(0%)	0	(0%)	203	(100%)	206	(100%)	409	(100%)
**Weight cohort**												
>=14kg	154	(81%)	157	(81%)	311	(81%)	196	(97%)	200	(97%)	396	(97%)
<14kg	35	(19%)	37	(19%)	72	(19%)	7	(3%)	6	(3%)	13	(3%)
**Country/region of residence**												
Europe	9	(5%)	9	(5%)	18	(5%)	3	(1%)	4	(2%)	7	(2%)
South Africa	44	(23%)	53	(27%)	97	(25%)	25	(12%)	42	(20%)	67	(16%)
Thailand	24	(13%)	26	(13%)	50	(13%)	4	(2%)	7	(3%)	11	(3%)
Uganda	63	(33%)	69	(36%)	132	(34%)	129	(64%)	113	(55%)	242	(59%)
Zimbabwe	49	(26%)	37	(19%)	86	(22%)	42	(21%)	40	(19%)	82	(20%)
**Sex**												
male	81	(43%)	101	(52%)	182	(48%)	111	(55%)	110	(53%)	221	(54%)
female	108	(57%)	93	(48%)	201	(52%)	92	(45%)	96	(47%)	188	(46%)
**Age (years)**												
n	189		194		383		203		206		409	
median	10.5		10.4		10.5		12.4		12.4		12.4	
[IQR]	[6.9, 13.6]		[6.3, 14.0]		[6.5, 14.0]		[9.0, 15.2]		[8.8, 14.6]		[9.0, 14.8]	
[range]	[0.3-17.8]		[0.1-18.0]		[0.1-18.0]		[2.0-18.0]		[1.9-18.0]		[1.9-18.0]	
**Weight (kg)**												
n	189		194		383		203		206		409	
median	26.3		26.4		26.4		30.3		31.6		30.7	
[IQR]	[18.4, 41.0]		[18.2, 40.6]		[18.4, 41.0]		[22.9, 42.5]		[23.1, 41.0]		[23.0, 41.6]	
[range]	[3.8-85.0]		[3.4-72.7]		[3.4-85.0]		[8.8-71.5]		[8.0-65.4]		[8.0-71.5]	
**CD4 lymphocyte percentage**												
n	188		191		379		203		206		409	
median	19		20		20		21		24		23	
[IQR]	[13, 28]		[10, 28]		[12, 28]		[11, 32]		[15, 32]		[13, 32]	
[range]	[0.8-50.0]		[0.9-69.5]		[0.8-69.5]		[0.0-59.0]		[0.5-52.5]		[0.0-59.0]	
<15	59	(31%)	66	(35%)	125	(33%)	69	(34%)	53	(26%)	122	(30%)

15-<30	94 (50%)	83 (43%)	177 (47%)	80 (39%)	82 (40%)	162 (40%)
>=30	35 (19%)	42 (22%)	77 (20%)	54 (27%)	71 (34%)	125 (31%)
missing	1	3	4	0	0	0
**CD4 lymphocyte count (cells/mm3)**						
n	188	191	379	203	206	409
median	505	474	496	444	564	492
[IQR]	[238, 829]	[247, 900]	[245, 862]	[196, 753]	[289, 802]	[250, 792]
[range]	[6.5-4675.5]	[7.0-5299.5]	[6.5-5299.5]	[3.5-2672.0]	[2.0-3637.5]	[2.0-3637.5]
<50	20 (11%)	19 (10%)	39 (10%)	20 (10%)	19 (9%)	39 (10%)
50-<100	11 (6%)	10 (5%)	21 (6%)	11 (5%)	4 (2%)	15 (4%)
100-<200	9 (5%)	13 (7%)	22 (6%)	20 (10%)	9 (4%)	29 (7%)
200-<350	22 (12%)	24 (13%)	46 (12%)	26 (13%)	31 (15%)	57 (14%)
350-<500	28 (15%)	34 (18%)	62 (16%)	44 (22%)	28 (14%)	72 (18%)
500-<1000	59 (31%)	48 (25%)	107 (28%)	50 (25%)	83 (40%)	133 (33%)
1000-1500	18 (10%)	28 (15%)	46 (12%)	22 (11%)	25 (12%)	47 (11%)
>=1500	21 (11%)	15 (8%)	36 (9%)	10 (5%)	7 (3%)	17 (4%)
missing	1	3	4	0	0	0
**Viral load (copies/mL)**						
<400	5 (3%)	11 (6%)	16 (4%)	0 (0%)	0 (0%)	0 (0%)
400-<1,000	9 (5%)	5 (3%)	14 (4%)	4 (2%)	3 (1%)	7 (2%)
1,000-<10,000	30 (16%)	36 (19%)	66 (17%)	49 (24%)	75 (36%)	124 (30%)
10,000-<50,000	47 (25%)	38 (20%)	85 (22%)	77 (38%)	77 (37%)	154 (38%)
50,000-<100,000	25 (13%)	22 (12%)	47 (12%)	23 (11%)	26 (13%)	49 (12%)
100,000-<500,000	51 (27%)	56 (30%)	107 (28%)	43 (21%)	20 (10%)	63 (15%)
500,000-<1,000,000	11 (6%)	12 (6%)	23 (6%)	4 (2%)	4 (2%)	8 (2%)
>=1,000,000	11 (6%)	9 (5%)	20 (5%)	3 (1%)	1 (0%)	4 (1%)
missing	0	5	5	0	0	0
**Log10 Viral load (log10 copies/mL)**						
n	189	189	378	203	206	409
median	4.7	4.8	4.7	4.4	4.2	4.3
[IQR]	[4.1, 5.3]	[3.9, 5.3]	[4.0, 5.3]	[3.9, 5.0]	[3.7, 4.7]	[3.8, 4.8]
[range]	[1.3-6.6]	[1.3-7.0]	[1.3-7.0]	[2.7-6.2]	[2.9-6.1]	[2.7-6.2]
**History of WHO staging**						
stage1	78 (41%)	82 (42%)	160 (42%)	66 (33%)	80 (39%)	146 (36%)
stage2	63 (33%)	64 (33%)	127 (33%)	77 (38%)	64 (31%)	141 (34%)
stage3	31 (16%)	30 (15%)	61 (16%)	44 (22%)	38 (18%)	82 (20%)

stage4	17	(9%)	18	(9%)	35	(9%)	16	(8%)	24	(12%)	40	(10%)
**NRTI backbone at randomisation**												
ABC 3TC	161	(85%)	158	(81%)	319	(83%)	109	(54%)	110	(53%)	219	(54%)
ABC TDF	0	(0%)	0	(0%)	0	(0%)	1	(0%)	2	(1%)	3	(1%)
TDF/TAF 3TC/FTC	28	(15%)	32	(16%)	60	(16%)	52	(26%)	52	(25%)	104	(25%)
ZDV 3TC	0	(0%)	4	(2%)	4	(1%)	41	(20%)	42	(20%)	83	(20%)
**Anchor drug class at randomisation**												
INSTI	189	(100%)	1	(1%)	190	(50%)	203	(100%)	2	(1%)	205	(50%)
NNRTI	0	(0%)	157	(81%)	157	(41%)	0	(0%)	6	(3%)	6	(1%)
PI	0	(0%)	36	(19%)	36	(9%)	0	(0%)	198	(96%)	198	(48%)
**Anchor drug at randomisation**												
ATV	0	(0%)	0	(0%)	0	(0%)	0	(0%)	49	(24%)	49	(12%)
DRV	0	(0%)	4	(2%)	4	(1%)	0	(0%)	2	(1%)	2	(0%)
DTG	189	(100%)	0	(0%)	189	(49%)	203	(100%)	0	(0%)	203	(50%)
EFV	0	(0%)	149	(77%)	149	(39%)	0	(0%)	5	(2%)	5	(1%)
EVG	0	(0%)	1	(1%)	1	(0%)	0	(0%)	0	(0%)	0	(0%)
LOP	0	(0%)	32	(16%)	32	(8%)	0	(0%)	147	(71%)	147	(36%)
NVP	0	(0%)	6	(3%)	6	(2%)	0	(0%)	1	(0%)	1	(0%)
RAL	0	(0%)	0	(0%)	0	(0%)	0	(0%)	2	(1%)	2	(0%)
RLP	0	(0%)	2	(1%)	2	(1%)	0	(0%)	0	(0%)	0	(0%)

Data are n(%), median [IQR], or median [IQR; range].

§ At a participant level, the mean of the measured values was used if measured values were available at screening and randomisation.

‡ Worst known stage prior to enrolment.

¶ Two ≥14kg cohort participants in the SOC group initiated tenofovir alafenamide and emtricitabine (one in ODYSSEY A and one in ODYSSEY B).

Abbreviations: 3TC=lamivudine. ABC=abacavir. ATV=atazanavir. DRV=darunavir. DTG=dolutegravir. EFV=efavirenz. EVG=elvitegravir. FTC=emtricitabine. INSTI=integrase inhibitor. IQR=interquartile range. LPV=lopinavir. NRTI=nucleoside reverse transcriptase inhibitors. NNRTI=non-nucleoside reverse transcriptase inhibitors. NVP=nevirapine. PI=protease inhibitor. RAL=raltegravir. RLP=rilpivirine. SOC=standard of care. TAF=tenofovir alafenamide. TDF=tenofovir disoproxil fumarate. WHO= World Health Organization. ZDV=zidovudine.

Figure S3. Time to switch off randomised allocation during randomised follow-up*

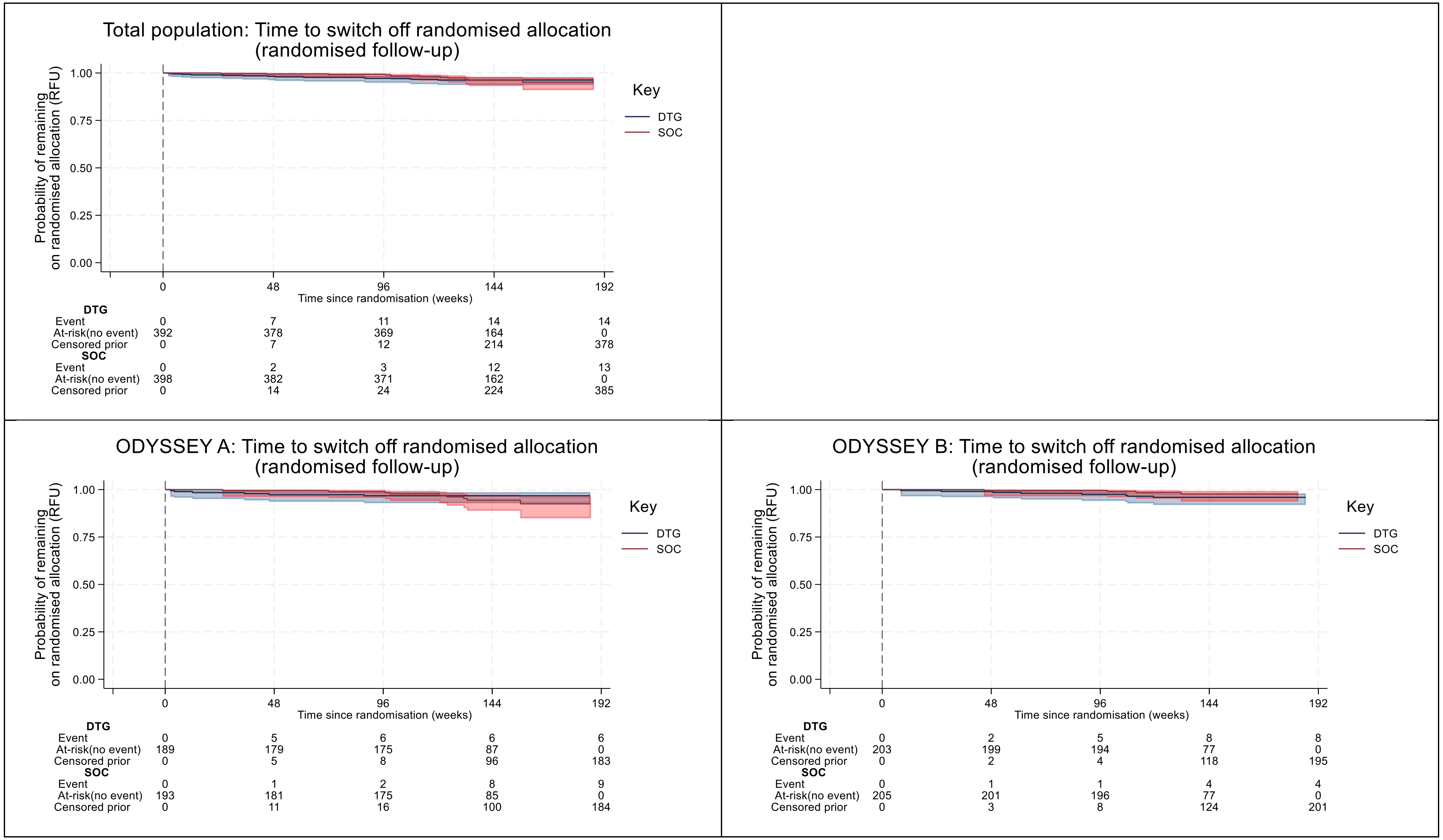

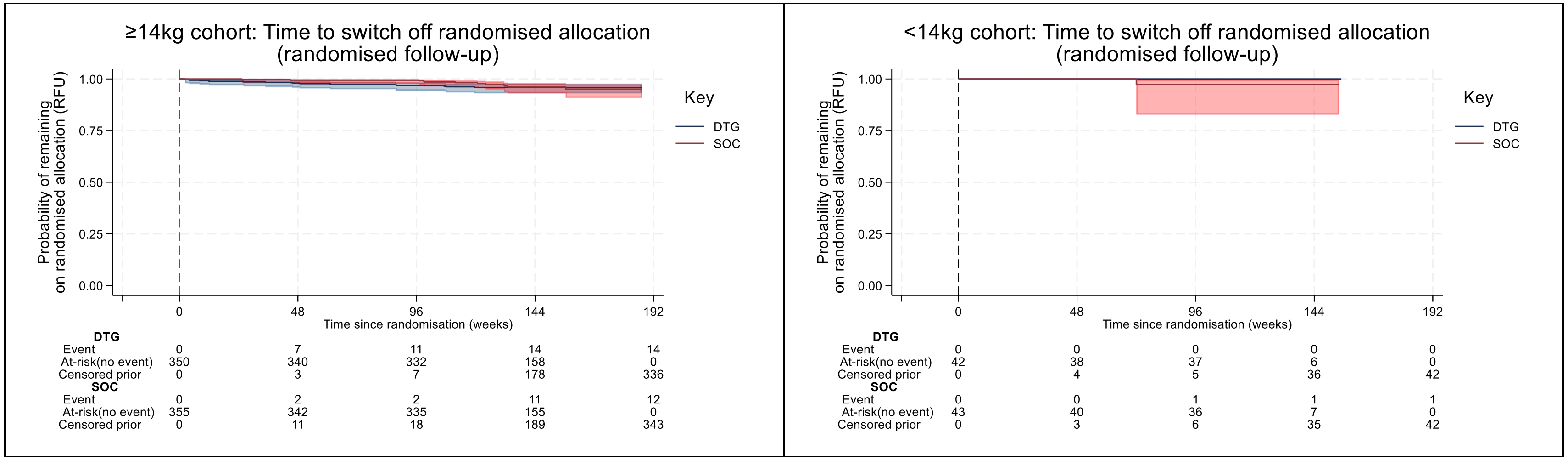

** Randomised follow-up was until the last participant reached week 96 (≥14kg cohort, 24 April 2020; <14kg cohort, 28 June 2021).*

Abbreviations: DTG=dolutegravir. RFU=randomised follow-up. SOC=standard of care.

Figure S4. Time to switch off randomised allocation during trial (randomised* and extended$^{\alpha}$ follow-up)

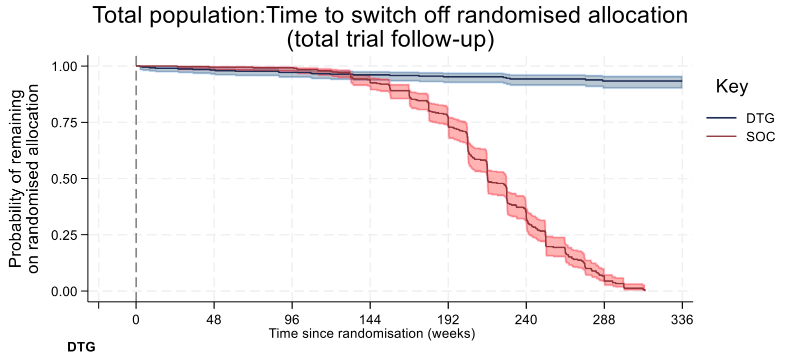

	0	48	96	144	192	240	288	336
**DTG**								
Event	0	7	11	15	18	21	23	23
At-risk(no event)	392	378	369	346	327	284	185	16
Censored prior	0	7	12	31	47	87	184	353
**SOC**								
Event	0	2	3	27	90	220	298	309
At-risk(no event)	398	382	372	325	241	97	13	0
Censored prior	0	14	23	46	67	81	87	89

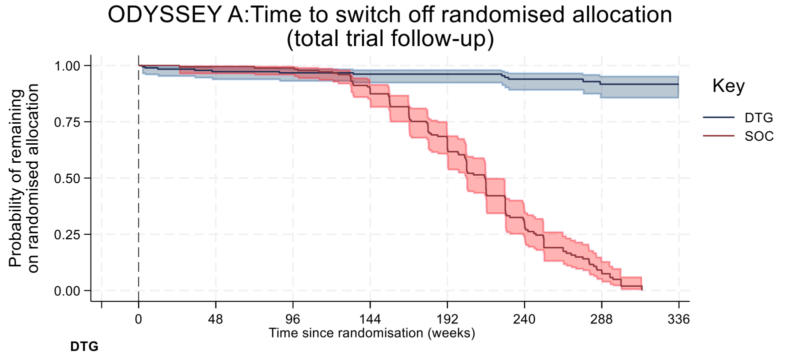

	0	48	96	144	192	240	288	336
**DTG**								
Event	0	5	6	7	7	10	12	12
At-risk(no event)	189	179	175	160	150	117	79	10
Censored prior	0	5	8	22	32	62	98	167
**SOC**								
Event	0	1	2	21	58	105	131	139
At-risk(no event)	193	181	176	141	93	38	10	0
Censored prior	0	11	15	31	42	50	52	54

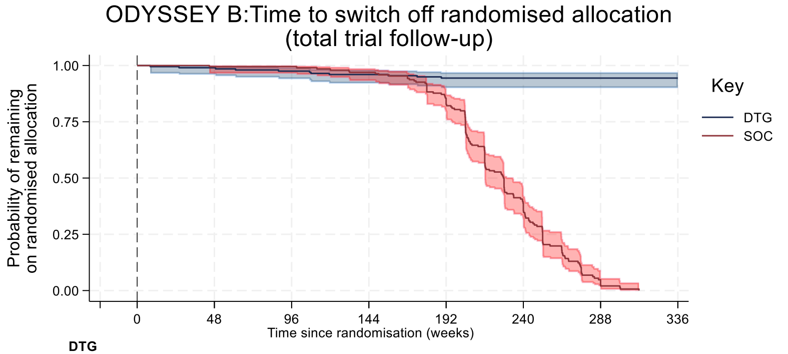

	0	48	96	144	192	240	288	336
**DTG**								
Event	0	2	5	8	11	11	11	11
At-risk(no event)	203	199	194	186	177	167	106	6
Censored prior	0	2	4	9	15	25	86	186
**SOC**								
Event	0	1	1	6	32	115	167	170
At-risk(no event)	205	201	196	184	148	59	3	0
Censored prior	0	3	8	15	25	31	35	35

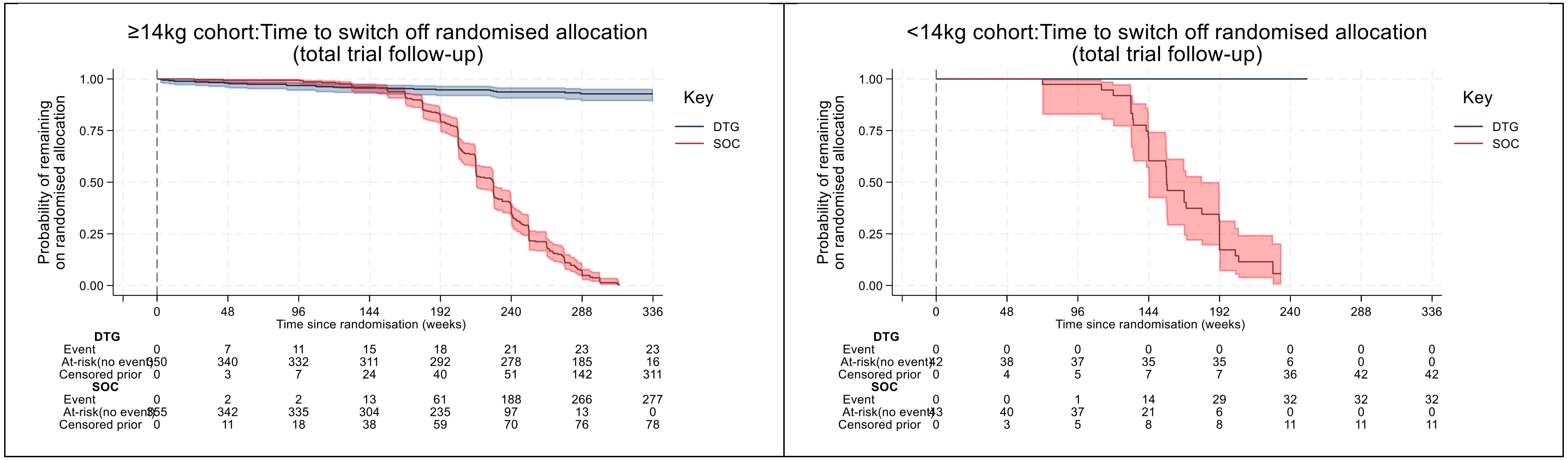

** Randomised follow-up was until the last participant reached week 96 (≥14kg cohort, 24 April 2020; <14kg cohort, 28 June 2021).*
α Extended follow-up continued until 01 May 2023, when the last participant in the ≥14kg cohort reached 240 weeks and the last participant in the <14kg cohort reached 192 weeks.

Abbreviations: DTG=dolutegravir. SOC=standard of care.

Figure S5. Time to switch off randomised allocation or censoring during trial (randomised* and extended$^{\alpha}$ follow-up)

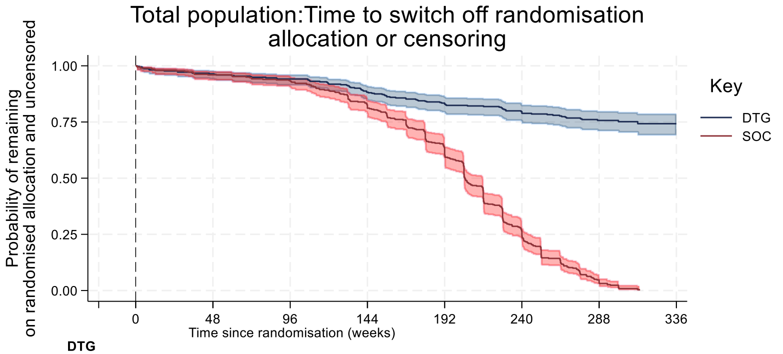

	0	48	96	144	192	240	288	336
**DTG**								
Event	0	14	23	46	67	82	92	94
At-risk(no event)	392	378	369	346	325	281	185	16
Censored prior	0	0	0	0	0	29	115	282
**SOC**								
Event	0	16	27	75	158	301	382	393
At-risk(no event)	398	382	371	323	240	95	13	0
Censored prior	0	0	0	0	0	2	3	5

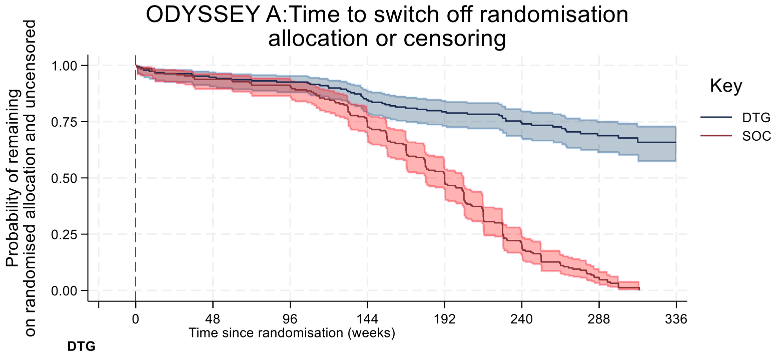

	0	48	96	144	192	240	288	336
**DTG**								
Event	0	10	14	29	39	48	55	57
At-risk(no event)	189	179	175	160	150	116	79	10
Censored prior	0	0	0	0	0	25	55	122
**SOC**								
Event	0	12	18	53	100	155	182	190
At-risk(no event)	193	181	175	140	93	37	10	0
Censored prior	0	0	0	0	0	1	1	3

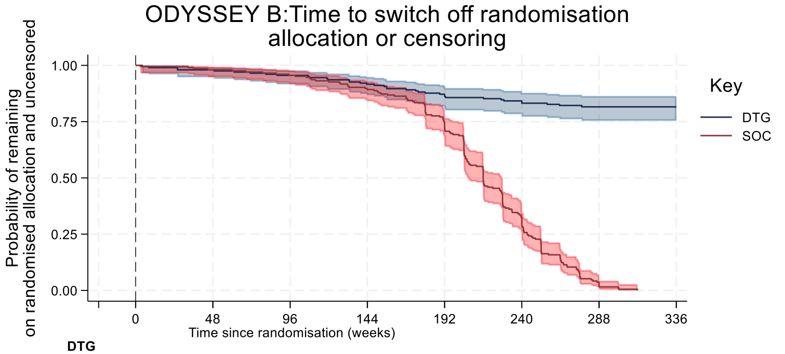

	0	48	96	144	192	240	288	336
**DTG**								
Event	0	4	9	17	28	34	37	37
At-risk(no event)	203	199	194	186	175	165	106	6
Censored prior	0	0	0	0	0	4	60	160
**SOC**								
Event	0	4	9	22	58	146	200	203
At-risk(no event)	205	201	196	183	147	58	3	0
Censored prior	0	0	0	0	0	1	2	2

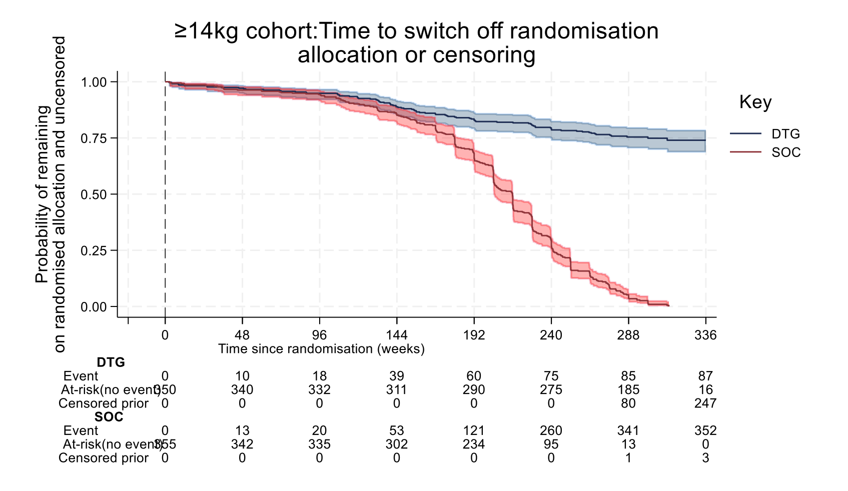

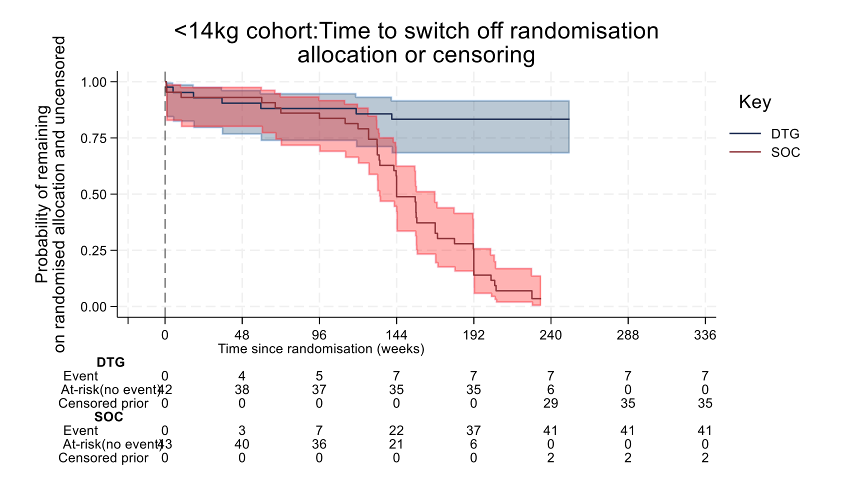

** Randomised follow-up was until the last participant reached week 96 (≥14kg cohort, 24 April 2020; <14kg cohort, 28 June 2021).*
α Extended follow-up continued until 01 May 2023, when the last participant in the ≥14kg cohort reached 240 weeks and the last participant in the <14kg cohort reached 192 week.

Abbreviations: DTG=dolutegravir. SOC=standard of care.

Table S3. On randomised allocation: Comparison of proportion with treatment failure by visit week

		DTG		SOC				
**Visit week**	**Cohort**	**n**	**Estimated percentage (95% CI)**	**n**	**Estimated percentage (95% CI)**	**Risk difference (95% CI)**	**Pinteraction (treatment group vs. ODYSSEY A or B)**	**Pinteraction (treatment group vs. >=14kg or <14kg)**
**Week 48**	Total population	27	7% (5 to 9)	57	14% (11 to 17)	-7% [-11 to -3]; P=0.00086	P=0.55	P=0.56
	ODYSSEY A	13	7% (4 to 11)	33	15% (11 to 20)	-8% [-14 to -2]; P=0.012		
	ODYSSEY B	14	6% (4 to 10)	24	11% (8 to 15)	-5% [-10 to 0]; P=0.05		
	>=14kg cohort	20	5% (3 to 8)	42	12% (9 to 15)	-6% [-10 to -2]; P=0.0016		
	<14kg cohort	7	17% (8 to 28)	15	28% (18 to 40)	-12% [-28 to 5]; P=0.17		
**Week 96**	Total population	60	15% (12 to 19)	96	24% (20 to 28)	-9% [-14 to -4]; P=0.0012	P=0.2	P=0.97
	ODYSSEY A	25	13% (9 to 19)	51	25% (19 to 31)	-12% [-19 to -4]; P=0.0033		
	ODYSSEY B	35	17% (12 to 22)	45	22% (17 to 28)	-6% [-14 to 2]; P=0.16		
	>=14kg cohort	48	14% (10 to 17)	75	22% (18 to 27)	-9% [-14 to -3]; P=0.0035		
	<14kg cohort	12	27% (17 to 39)	21	39% (27 to 50)	-12% [-28 to 5]; P=0.17		
**Week 144**	Total population	68	17% (14 to 21)	114	29% (24 to 33)	-11% [-17 to -6]; P=0.00011	P=0.24	P=0.78
	ODYSSEY A	27	14% (10 to 20)	56	27% (21 to 34)	-13% [-21 to -5]; P=0.0016		
	ODYSSEY B	41	20% (15 to 26)	58	29% (23 to 35)	-9% [-17 to 0]; P=0.047		
	>=14kg cohort	56	16% (12 to 20)	91	27% (22 to 31)	-10% [-17 to -4]; P=0.00079		
	<14kg cohort	12	27% (17 to 39)	23	42% (28 to 55)	-15% [-32 to 3]; P=0.1		
**Week 192**	Total population	73	19% (15 to 23)	129	33% (29 to 38)	-14% [-20 to -8]; P<0.0001	P=0.12	P=0.79
	ODYSSEY A	27	15% (10 to 20)	62	31% (25 to 38)	-16% [-25 to -8]; P=0.00015		
	ODYSSEY B	46	23% (17 to 29)	67	34% (27 to 40)	-11% [-20 to -2]; P=0.018		
	>=14kg cohort	61	18% (14 to 22)	106	31% (26 to 36)	-13% [-20 to -7]; P<0.0001		
	<14kg cohort	12	26% (16 to 38)	23	41% (27 to 55)	-15% [-32 to 3]; P=0.1		
**Week 240**	Total population	74	19% (16 to 23)	131	34% (30 to 39)	-15% [-21 to -9]; P<0.0001	P=0.1	

ODYSSEY A	27	15% (10 to 20)	63	32% (25 to 39)	-17% [-26 to -9]; P=0.0001
ODYSSEY B	47	24% (18 to 30)	68	35% (28 to 42)	-11% [-21 to -2]; P=0.015
>=14kg cohort	62	18% (14 to 23)	108	33% (28 to 38)	-14% [-21 to -8]; P<0.0001

On-randomised allocation effects were estimated from marginal structural models with stabilised inverse probability weights to account for switch off randomised treatment regimen and censoring due to death, loss to follow-up or administrative censoring (appendix, Supplementary statistical methods). Marginal probabilities of treatment failure and absolute differences between DTG and SOC in the trial population estimated using flexible parametric survival models fitted on the log cumulative hazard scale using restricted cubic splines, adjusted for baseline covariates including ODYSSEY A/B, country/region, month and year of randomisation (spline), sex, age, weight, BMI-for-age Z-score, CD4 count (CD4% for <14kg cohort only models), and $\log_{10}$ viral load.

Analyses on randomised allocation are reported to week 240, except where we report results separately for the <14kg cohort when we report to week 192. Sub-group differences (ODYSSEY A vs B; ≥14kg vs <14kg) in the effect of ART-regimen on the risk of treatment failure were estimated using data to each of the 48-weekly timepoints.

Numbers of participants experiencing virological failure contributing to model at each specified time-point are reported, i.e. numbers remaining on randomised allocation by given; estimated proportions and treatment differences are estimated by weighting observations to account for participants switching off randomised allocation and censoring.

Abbreviations: CI=confidence interval. *DTG=dolutegravir. n=number. SOC=standard of care.*

Table S4. Intention-to-treat: Comparison of proportion with treatment failure by visit week

		**DTG**		**SOC**				
**Visit week**	**Cohort**	**n**	**Estimated percentage (95% CI)**	**n**	**Estimated percentage (95% CI)**	**Risk difference (95% CI)**	**Pinteraction (treatment group vs. ODYSSEY A or B)**	**Pinteraction (treatment group vs. >=14kg or <14kg)**
**Week 48**	Total population	27	7% (5 to 10)	57	14% (11 to 17)	-7% [-11 to -3]; P=0.00065	P=0.57	P=0.62
	ODYSSEY A	13	7% (4 to 12)	33	16% (12 to 21)	-9% [-15 to -2]; P=0.0065		
	ODYSSEY B	14	7% (4 to 10)	24	11% (8 to 15)	-5% [-10 to 1]; P=0.079		
	>=14kg cohort	20	5% (4 to 8)	42	12% (9 to 15)	-7% [-11 to -3]; P=0.0014		
	<14kg cohort	7	18% (10 to 29)	15	30% (19 to 41)	-11% [-27 to 4]; P=0.16		
**Week 96**	Total population	59	15% (12 to 19)	96	25% (21 to 29)	-9% [-15 to -4]; P=0.00083	P=0.18	P=0.92
	ODYSSEY A	24	14% (9 to 19)	51	26% (21 to 32)	-13% [-20 to -5]; P=0.0014		
	ODYSSEY B	35	17% (12 to 23)	45	23% (17 to 29)	-5% [-13 to 3]; P=0.2		
	>=14kg cohort	47	13% (10 to 17)	75	22% (18 to 27)	-9% [-14 to -3]; P=0.0028		
	<14kg cohort	12	32% (20 to 44)	21	45% (33 to 56)	-13% [-30 to 3]; P=0.12		
**Week 144**	Total population	70	18% (15 to 22)	114	29% (25 to 34)	-11% [-17 to -5]; P=0.00019	P=0.27	P=0.64
	ODYSSEY A	28	16% (11 to 21)	56	29% (23 to 35)	-13% [-21 to -5]; P=0.0013		
	ODYSSEY B	42	21% (16 to 27)	58	29% (23 to 36)	-8% [-17 to 1]; P=0.069		
	>=14kg cohort	58	17% (13 to 21)	91	27% (22 to 31)	-10% [-16 to -4]; P=0.0013		
	<14kg cohort	12	33% (21 to 46)	23	49% (37 to 61)	-16% [-34 to 1]; P=0.069		
**Week 192**	Total population	76	20% (16 to 24)	129	34% (29 to 38)	-14% [-20 to -7]; P<0.0001	P=0.14	
	ODYSSEY A	28	16% (11 to 22)	62	33% (26 to 39)	-17% [-25 to -8]; P=0.00014		
	ODYSSEY B	48	24% (19 to 31)	67	34% (28 to 41)	-10% [-19 to -1]; P=0.034		
	>=14kg cohort	64	19% (15 to 23)	106	31% (26 to 36)	-13% [-19 to -6]; P=0.00011		

Marginal probabilities of treatment failure and absolute differences between DTG and SOC in the trial population estimated using flexible parametric survival models fitted on the log cumulative hazard scale using restricted cubic splines, adjusted for baseline covariates including ODYSSEY A/B, country/region, month and year of randomisation (spline), sex, age, weight, BMI-for-age Z-score, CD4 count (CD4% for <14kg cohort only models), and $\log_{10}$ viral load.

Analyses in the intention to treat population are reported to week 192, except where we report results separately for the <14kg cohort when we report to week 144. Sub-group differences (ODYSSEY A vs B; ≥14kg vs <14kg) in the effect of ART-regimen on the risk of treatment failure were estimated using data to each of the 48-weekly timepoints.

Abbreviations: CI=confidence interval. *DTG=dolutegravir. n=number. SOC=standard of care.*

Table S5. On randomised allocation: Comparison of proportion of participants with HIV-1 RNA <400 copies/mL by visit week

		DTG		SOC		
**Visit week**	**Cohort**	**n/N**	**Estimated percentage (95% CI)**	**n/N**	**Estimated percentage (95% CI)**	**Risk difference (95% CI)**
**Week 48**	Total population	327/374	87% (84 to 90)	313/378	83% (79 to 87)	4% (-1 to 9); P=0.1
	ODYSSEY A	153/176	86% (81 to 91)	145/179	82% (76 to 87)	4% (-3 to 12); P=0.24
	ODYSSEY B	174/198	88% (84 to 93)	168/199	85% (80 to 90)	4% (-3 to 10); P=0.31
	>=14kg cohort	302/340	88% (85 to 92)	286/339	84% (80 to 88)	4% (-1 to 9); P=0.12
	<14kg cohort	25/34	75% (62 to 88)	27/39	73% (60 to 86)	2% (-17 to 21); P=0.82
**Week 96**	Total population	326/365	89% (87 to 92)	310/367	83% (80 to 86)	6% (2 to 10); P=0.0023
	ODYSSEY A	153/172	90% (86 to 94)	147/170	85% (81 to 89)	5% (-1 to 11); P=0.092
	ODYSSEY B	173/193	89% (85 to 92)	163/197	82% (78 to 86)	7% (1 to 12); P=0.018
	>=14kg cohort	293/329	89% (87 to 92)	284/332	84% (81 to 87)	5% (1 to 9); P=0.0086
	<14kg cohort	33/36	87% (78 to 97)	26/35	77% (65 to 89)	10% (-5 to 25); P=0.19
**Week 144**	Total population	289/326	89% (87 to 92)	255/306	82% (79 to 86)	7% (3 to 12); P=0.001
	ODYSSEY A	135/148	91% (87 to 95)	116/135	85% (80 to 90)	6% (-1 to 12); P=0.075
	ODYSSEY B	154/178	88% (84 to 92)	139/171	80% (75 to 84)	8% (2 to 14); P=0.0074
	>=14kg cohort	258/291	89% (86 to 92)	234/280	83% (79 to 86)	6% (2 to 11); P=0.0069
	<14kg cohort	31/35	91% (85 to 97)	21/26	77% (63 to 90)	14% (0 to 29); P=0.048
**Week 192**	Total population	265/293	88% (85 to 90)	178/221	80% (76 to 84)	8% (3 to 13); P=0.0009
	ODYSSEY A	118/126	90% (87 to 94)	70/82	84% (78 to 90)	7% (0 to 14); P=0.061
	ODYSSEY B	147/167	86% (83 to 90)	108/139	77% (72 to 83)	9% (2 to 15); P=0.007
	>=14kg cohort	234/260	88% (85 to 90)	170/210	81% (77 to 85)	7% (2 to 11); P=0.0058
	<14kg cohort	31/33	92% (83 to 100)	8/11	72% (49 to 96)	19% (-6 to 45); P=0.14
**Week 240**	Total population	230/267	86% (82 to 90)	84/102	77% (71 to 84)	8% (1 to 16); P=0.027
	ODYSSEY A	96/108	89% (84 to 95)	30/37	81% (71 to 91)	8% (-4 to 19); P=0.18
	ODYSSEY B	134/159	84% (79 to 89)	54/65	75% (66 to 83)	9% (-1 to 19); P=0.075
	>=14kg cohort	224/261	86% (82 to 90)	84/102	79% (72 to 85)	7% (0 to 14); P=0.067

On-randomised allocation effects were estimated from marginal structural models with stabilised inverse probability weights to account for switch off randomised treatment regimen and

censoring due to death, loss to follow-up or administrative censoring (appendix, Supplementary statistical methods). Estimated proportions with HIV-1 RNA <400copies/mL (95% CI) up to 240 weeks (up to 192 weeks when estimating for <14kg cohort only) by trial arm and difference between trial arms (SOC as reference) are marginal estimates calculated from logistic mixed models with random intercept for participants and fixed effects for trial arm and visit weeks (3 knot cubic spline), including interaction between trial arm and visit weeks, adjusting for baseline variables: ODYSSEY A/B, country/region, calendar month of randomisation (3 knot cubic spline), sex, age, weight, BMI-for-age Z-score, CD4 (CD4% for <14kg cohort only models), and log10 viral load.

*n/N denotes the number of participants contributing to the numerator and denominator in the model at specified time-point, i.e. numbers remaining on randomised allocation with an observation available within the visit window. Note models are mixed models and include information across time-points (using cubic splines for time) and observations are weighted to account for participants switching off randomised allocation and censoring.

$P_{interaction}$ (treatment vs ODYSSEY A/B) to 240 weeks = 0.99, $P_{interaction}$ (treatment vs ≥14kg/<14kg cohort) to 192 weeks = 0.51

Abbreviations: CI=confidence interval. *DTG=dolutegravir. n=number. SOC=standard of care.*

Table S6. On randomised allocation: Comparison of proportion of participants with HIV-1 RNA <50 copies/ml by visit week

		DTG		SOC		
**Visit week**	**Cohort**	**n/N**	**Estimated percentage (95% CI)**	**n/N**	**Estimated percentage (95% CI)**	**Risk difference (95% CI)**
**Week 48**	Total population	276/371	74% (70 to 79)	267/375	71% (66 to 75)	3% (-3 to 10); P=0.28
	ODYSSEY A	124/174	71% (64 to 77)	131/179	73% (67 to 80)	-3% (-12 to 6); P=0.54
	ODYSSEY B	152/197	78% (72 to 83)	136/196	69% (63 to 76)	9% (0 to 17); P=0.045
	>=14kg cohort	261/337	77% (73 to 82)	248/336	73% (68 to 78)	4% (-2 to 11); P=0.18
	<14kg cohort	15/34	44% (28 to 60)	19/39	53% (38 to 67)	-9% (-31 to 13); P=0.44
**Week 96**	Total population	291/362	79% (76 to 83)	270/366	73% (69 to 77)	6% (1 to 11); P=0.013
	ODYSSEY A	138/171	80% (76 to 85)	131/170	78% (73 to 83)	3% (-4 to 10); P=0.46
	ODYSSEY B	153/191	79% (75 to 83)	139/196	69% (64 to 75)	9% (3 to 16); P=0.0066
	>=14kg cohort	264/327	80% (76 to 83)	251/331	74% (70 to 78)	5% (0 to 10); P=0.041
	<14kg cohort	27/35	72% (59 to 85)	19/35	61% (47 to 75)	11% (-8 to 31); P=0.26
**Week 144**	Total population	260/325	81% (77 to 84)	226/304	73% (69 to 77)	8% (3 to 13); P=0.0024
	ODYSSEY A	123/147	84% (79 to 88)	109/134	79% (74 to 84)	5% (-2 to 12); P=0.19
	ODYSSEY B	137/178	78% (74 to 83)	117/170	68% (62 to 73)	11% (3 to 18); P=0.0042
	>=14kg cohort	232/291	80% (77 to 84)	208/278	74% (70 to 78)	6% (1 to 12); P=0.021
	<14kg cohort	28/34	83% (74 to 93)	18/26	65% (53 to 78)	18% (2 to 34); P=0.031
**Week 192**	Total population	236/293	80% (77 to 83)	154/220	70% (66 to 75)	9% (4 to 15); P=0.001
	ODYSSEY A	108/126	84% (79 to 88)	63/81	78% (72 to 85)	5% (-3 to 13); P=0.19
	ODYSSEY B	128/167	77% (73 to 81)	91/139	64% (58 to 71)	13% (5 to 21); P=0.0014
	>=14kg cohort	206/260	79% (76 to 83)	147/209	72% (67 to 76)	8% (2 to 14); P=0.0067
	<14kg cohort	30/33	87% (77 to 98)	7/11	68% (43 to 92)	20% (-8 to 48); P=0.16
**Week 240**	Total population	211/267	78% (74 to 82)	73/101	67% (60 to 75)	11% (2 to 19); P=0.018
	ODYSSEY A	90/108	82% (76 to 89)	29/37	77% (66 to 88)	5% (-8 to 19); P=0.41
	ODYSSEY B	121/159	75% (69 to 81)	44/64	61% (51 to 70)	15% (3 to 26); P=0.012
	>=14kg cohort	207/261	78% (74 to 83)	73/101	69% (61 to 76)	10% (1 to 18); P=0.034

On-randomised allocation effects were estimated from marginal structural models with stabilised inverse probability weights to account for switch off randomised treatment regimen and

censoring due to death, loss to follow-up or administrative censoring (appendix, Supplementary statistical methods). Estimated proportions with HIV-1 RNA <50copies/mL (95% CI) up to 240 weeks (up to 192 weeks when estimating for <14kg cohort only) by trial arm and difference between trial arms (SOC as reference) are marginal estimates calculated from logistic mixed models with random intercept for participants and fixed effects for trial arm and visit weeks (3 knot cubic spline), including interaction between trial arm and visit weeks, adjusting for baseline variables: ODYSSEY A/B, country/region, calendar month of randomisation (3 knot cubic spline), sex, age, weight, BMI-for-age Z-score, CD4 (CD4% for <14kg cohort only models), and log10 viral load.

*n/N denotes the number of participants contributing to the numerator and denominator in the model at specified time-point, i.e. numbers remaining on randomised allocation with an observation available within the visit window. Note models are mixed models and include information across time-points (using cubic splines for time) and observations are weighted to account for participants switching off randomised allocation and censoring.

$P_{interaction}$ (treatment vs ODYSSEY A/B) to 240 weeks = 0.23, $P_{interaction}$ (treatment vs ≥14kg/<14kg cohort) to 192 weeks = 0.27

Abbreviations: CI=confidence interval. *DTG=dolutegravir. n=number. SOC=standard of care.*

Table S7. On randomised allocation: Comparison of proportion of participants with HIV-1 RNA <1000 copies/ml by visit week

		DTG		SOC		
**Visit week**	**Cohort**	**n/N**	**Estimated percentage (95% CI)**	**n/N**	**Estimated percentage (95% CI)**	**Risk difference (95% CI)**
**Week 48**	Total population	337/374	90% (87 to 93)	324/378	86% (82 to 89)	4% (-1 to 8); P=0.11
	ODYSSEY A	160/176	90% (86 to 94)	149/179	83% (78 to 89)	7% (0 to 13); P=0.056
	ODYSSEY B	177/198	89% (85 to 94)	175/199	89% (84 to 93)	1% (-5 to 7); P=0.81
	>=14kg cohort	309/340	90% (87 to 93)	296/339	87% (84 to 91)	3% (-2 to 8); P=0.21
	<14kg cohort	28/34	83% (72 to 95)	28/39	76% (64 to 88)	8% (-9 to 25); P=0.38
**Week 96**	Total population	333/365	91% (89 to 93)	318/367	86% (83 to 89)	5% (2 to 9); P=0.0054
	ODYSSEY A	157/172	92% (88 to 95)	150/170	87% (83 to 91)	4% (-1 to 10); P=0.11
	ODYSSEY B	176/193	90% (87 to 93)	168/197	85% (81 to 89)	5% (0 to 10); P=0.041
	>=14kg cohort	299/329	91% (88 to 93)	292/332	86% (84 to 89)	4% (1 to 8); P=0.022
	<14kg cohort	34/36	91% (84 to 99)	26/35	78% (66 to 90)	13% (-1 to 27); P=0.068
**Week 144**	Total population	294/326	91% (88 to 93)	262/306	84% (81 to 88)	6% (2 to 10); P=0.0029
	ODYSSEY A	136/148	92% (89 to 96)	120/135	88% (83 to 93)	4% (-2 to 10); P=0.15
	ODYSSEY B	158/178	90% (86 to 93)	142/171	82% (77 to 87)	8% (2 to 13); P=0.0088
	>=14kg cohort	262/291	90% (88 to 93)	240/280	85% (82 to 89)	5% (1 to 10); P=0.014
	<14kg cohort	32/35	93% (88 to 98)	22/26	81% (70 to 93)	12% (-1 to 24); P=0.068
**Week 192**	Total population	270/293	89% (87 to 92)	186/221	82% (79 to 86)	7% (3 to 12); P=0.002
	ODYSSEY A	120/126	92% (89 to 95)	73/82	87% (81 to 92)	5% (-1 to 12); P=0.1
	ODYSSEY B	150/167	87% (84 to 91)	113/139	79% (74 to 85)	8% (2 to 14); P=0.0082
	>=14kg cohort	239/260	89% (87 to 92)	177/210	83% (79 to 87)	6% (2 to 11); P=0.0085
	<14kg cohort	31/33	92% (84 to 100)	9/11	85% (67 to 102)	7% (-12 to 27); P=0.45
**Week 240**	Total population	235/267	88% (84 to 91)	86/102	80% (74 to 86)	8% (1 to 15); P=0.034
	ODYSSEY A	99/108	92% (87 to 97)	31/37	85% (75 to 94)	7% (-4 to 18); P=0.2
	ODYSSEY B	136/159	85% (80 to 90)	55/65	76% (68 to 85)	8% (-1 to 18); P=0.09
	>=14kg cohort	229/261	87% (84 to 91)	86/102	81% (74 to 87)	7% (0 to 14); P=0.065

On-randomised allocation effects were estimated from marginal structural models with stabilised inverse probability weights to account for switch off randomised treatment regimen and

censoring due to death, loss to follow-up or administrative censoring (appendix, Supplementary statistical methods). Estimated proportions with HIV-1 RNA <1000copies/mL (95% CI) up to 240 weeks (up to 192 weeks when estimating for <14kg cohort only) by trial arm and difference between trial arms (SOC as reference) are marginal estimates calculated from logistic mixed models with random intercept for participants and fixed effects for trial arm and visit weeks (3 knot cubic spline), including interaction between trial arm and visit weeks, adjusting for baseline variables: ODYSSEY A/B, country/region, calendar month of randomisation (3 knot cubic spline), sex, age, weight, BMI-for-age Z-score, CD4 (CD4% for <14kg cohort only models), and log10 viral load.

*n/N denotes the number of participants contributing to the numerator and denominator in the model at specified time-point, i.e. numbers remaining on randomised allocation with an observation available within the visit window. Note models are mixed models and include information across time-points (using cubic splines for time) and observations are weighted to account for participants switching off randomised allocation and censoring.

$P_{interaction}$ (treatment vs ODYSSEY A/B) to 240 weeks = 0.67, $P_{interaction}$ (treatment vs ≥14kg/<14kg cohort) to 192 weeks = 0.63

Abbreviations: CI=confidence interval. *DTG=dolutegravir. n=number. SOC=standard of care.*

Figure S6. On randomised allocation: Mean change in CD4 count from baseline

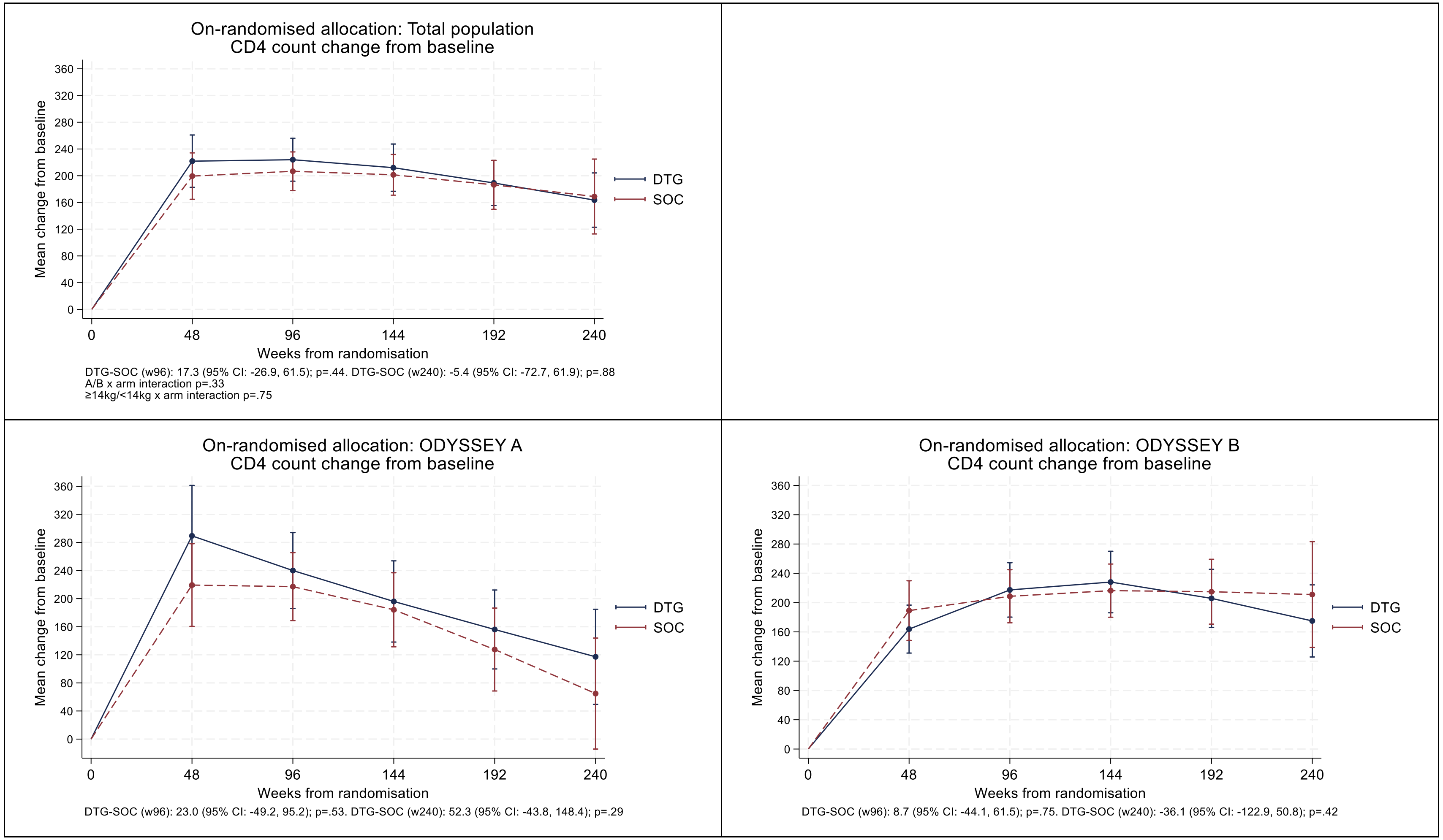

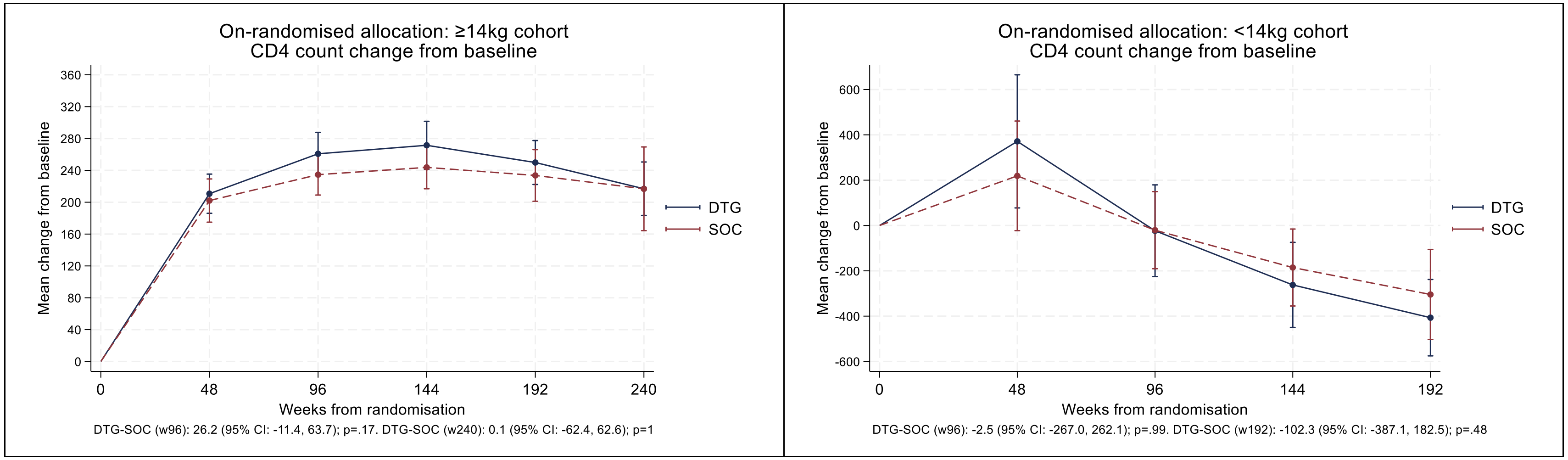

Footnote: Vertical bars represent 95% confidence intervals at each timepoint. Note difference in figure scale for <14kg cohort.

Abbreviations: CI=confidence interval. *DTG=dolutegravir. n=number. SOC=standard of care. W=week.*

Figure S7. On randomised allocation: Mean change in CD4 percentage from baseline

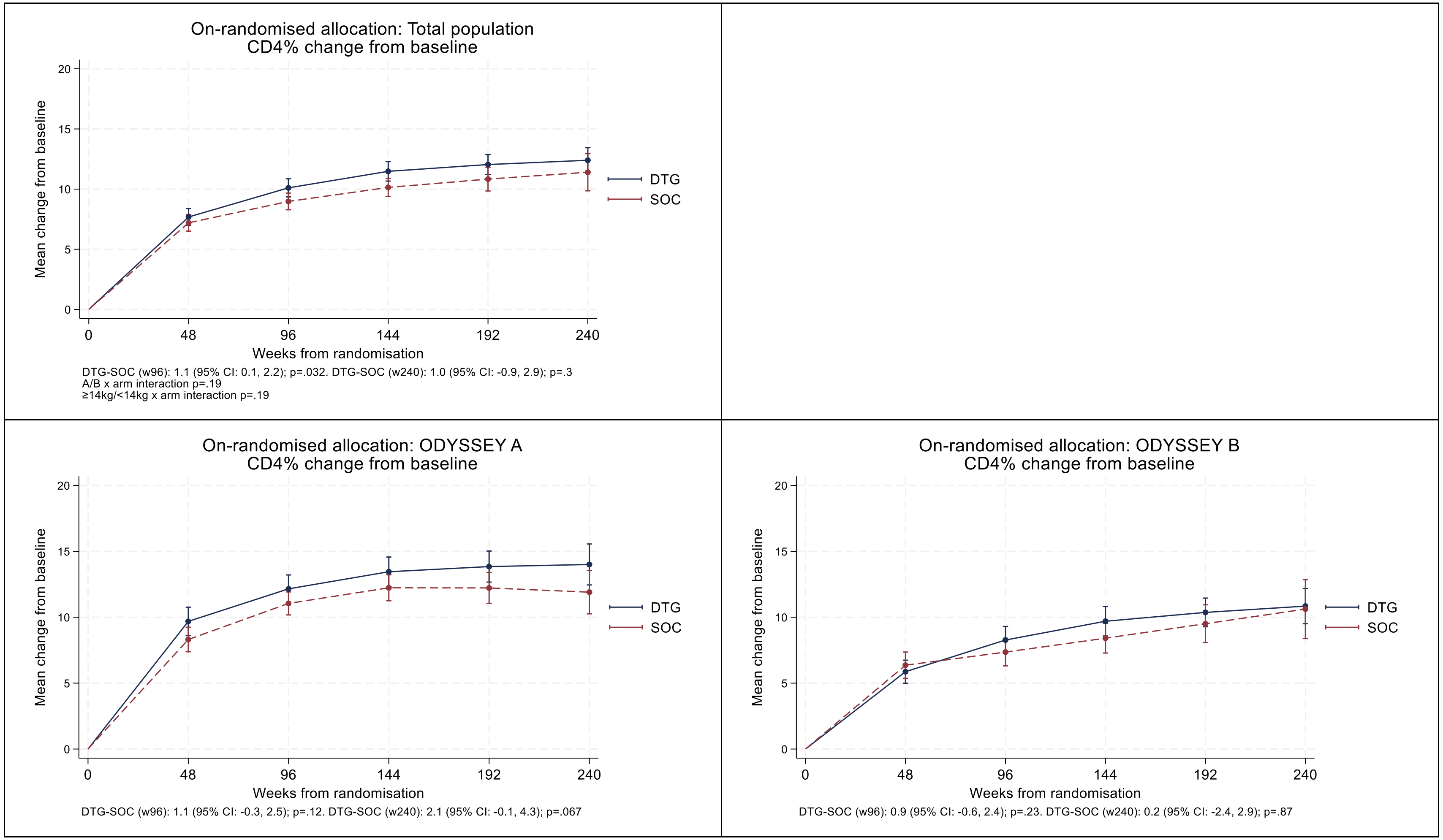

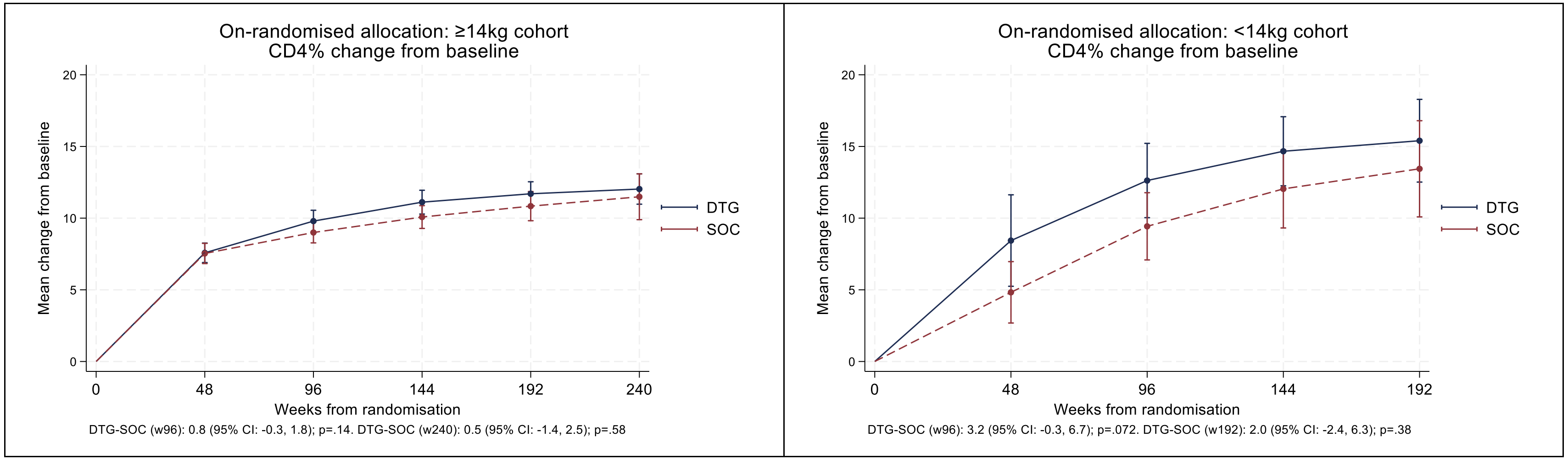

Footnote: Vertical bars represent 95% confidence intervals at each timepoint.

Abbreviations: CI=confidence interval. *DTG=dolutegravir. n=number. SOC=standard of care. W=week.*

Figure S8. Viral load<400c/mL after switching to DTG by pre-switch viral load in the SOC arm

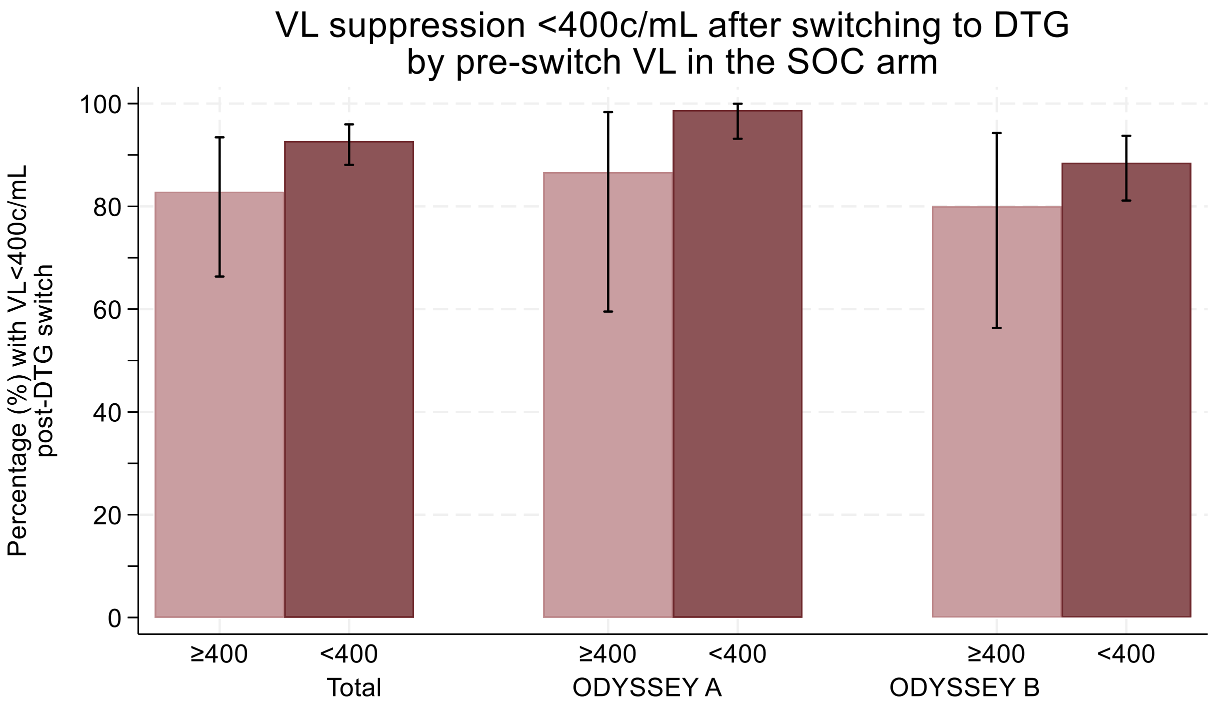

Cohort	Pre-switch viral load	n/N	Percentage with viral load <400c/mL post-DTG switch (95% CI)
**Total population**	≥400c/mL<br><400c/mL	29/35<br>178/192	83% (66-93)<br>93% (88-96)
**ODYSSEY A**	≥400c/mL<br><400c/mL	13/15<br>78/79	87% (60-98)<br>99% (93-100)
**ODYSSEY B**	≥400c/mL<br><400c/mL	16/20<br>100/113	80% (56-94)<br>88% (81-94)

Abbreviations: c/mL=copies per milliliter. CI=confidence interval. *DTG=dolutegravir. SOC=standard of care. VL=viral load.*

Table S8. Intention-to-treat: safety endpoints comparing dolutegravir with standard-of-care to 192 weeks, by ODYSSEY A and B

	**ODYSSEY A**			**ODYSSEY B**			
	**DTG (N=189)**	**SOC (N=194)**	**Treatment effect (95% CI)**	**DTG (N=203)**	**SOC (N=206)**	**Treatment effect (95% CI)**	**Pinteraction (treatment group vs. ODYSSEY A or B)**
**Severe WHO stage 3, WHO stage 4, or death - no. of events [no. of participants]**	10 [8]	11 [11]	HR=0.73 (0.29, 1.81); P=0.5	3 [3]	4 [4]	HR=0.75 (0.17, 3.37); P=0.71	P=0.96
**Serious adverse events - no. of events [no. of participants]**	68 [36]	51 [38]	HR=0.95 (0.60, 1.50); P=0.82	17 [16]	15 [15]	HR=1.06 (0.53, 2.15); P=0.86	P=0.78
**Grade ≥3 adverse events - no. of events [no. of participants]**	113 [67]	104 [68]	HR=0.99 (0.71, 1.39); P=0.95	44 [31]	88 [53]	HR=0.55 (0.35, 0.85); P=0.0075	P=0.04
**ART-modifying adverse events - no. of events [no. of participants]**	7 [6]	11 [10]	HR=0.58 (0.21, 1.60); P=0.29	2 [2]	13 [11]	HR=0.18 (0.04, 0.81); P=0.025	P=0.2
**Neuropsychiatric adverse events - no. of events [no. of participants]**	15 [12]	11 [6]	HR=1.99 (0.75, 5.32); P=0.17	5 [5]	5 [4]	HR=1.24 (0.33, 4.62); P=0.75	P=0.57
**Neurological adverse events - no. of events [no. of participants]**	4 [4]	7 [5]	HR=0.79 (0.21, 2.93); P=0.72	2 [2]	1 [1]	HR=1.99 (0.18, 21.94); P=0.57	P=0.51
**Psychiatric adverse events - no. of events [no. of participants]**	11 [9]	4 [2]	HR=4.45 (0.96, 20.61); P=0.056	3 [3]	4 [3]	HR=0.99 (0.20, 4.90); P=0.99	P=0.18

Hazard ratios are shown for time to first event; comparisons of treatment groups are unadjusted and are presented for the dolutegravir group compared with the standard of care group.

Abbreviations: CI=confidence interval. *DTG=dolutegravir. HR=hazard ratio. SOC=standard of care. VL=viral load. WHO=World Health Organization.*

Table S9. Intention-to-treat: safety endpoints comparing dolutegravir with standard-of-care to 144 weeks, by weight cohort

	>=14kg cohort			<14kg cohort			
	**DTG (N=350)**	**SOC (N=357)**	**Treatment effect (95% CI)**	**DTG (N=42)**	**SOC (N=43)**	**Treatment effect (95% CI)**	**Pinteraction (treatment group vs. >=14kg or <14kg)**
**Severe WHO stage 3, WHO stage 4, or death - no. of events [no. of participants]**	10 [8]	8 [8]	HR=1.00 (0.38, 2.67); P=1.01	3 [3]	6 [6]	HR=0.5 (0.13, 2.02); P=0.33	P=0.43
**Serious adverse events - no. of events [no. of participants]**	62 [35]	43 [39]	HR=0.89 (0.56, 1.40); P=0.6	15 [11]	19 [11]	HR=1.06 (0.46, 2.45); P=0.89	P=0.67
**Grade ≥3 adverse events - no. of events [no. of participants]**	108 [69]	138 [91]	HR=0.74 (0.54, 1.01); P=0.056	36 [19]	34 [21]	HR=0.93 (0.50, 1.73); P=0.82	P=0.49
**ART-modifying adverse events - no. of events [no. of participants]**	7 [6]	20 [19]	HR=0.31 (0.12, 0.78); P=0.012	0 [0]	2 [2]	-	-
**Neuropsychiatric adverse events - no. of events [no. of participants]**	16 [13]	13 [8]	HR=1.61 (0.67, 3.89); P=0.29	-	-	-	-
**Neurological adverse events - no. of events [no. of participants]**	6 [6]	6 [5]	HR=1.17 (0.36, 3.85); P=0.79	-	-	-	-
**Psychiatric adverse events - no. of events [no. of participants]**	10 [8]	7 [4]	HR=1.97 (0.59, 6.54); P=0.27	-	-	-	-

Hazard ratios are shown for time to first event; comparisons of treatment groups are adjusted for ODYSSEY A/B in ≥14kg cohort and unadjusted in <14kg, and are presented for the dolutegravir group compared with the standard of care group.

Abbreviations: CI=confidence interval. *DTG=dolutegravir. HR=hazard ratio. SOC=standard of care. VL=viral load. WHO=World Health Organization.*

Table S10. Intention-to-treat: severe WHO 3 event, WHO 4 event or death events to 192 weeks

	**Total**						**A**						**B**					
	**DTG**		**SOC**		**Total**		**DTG**		**SOC**		**Total**		**DTG**		**SOC**		**Total**	
**Participants randomised and included**	392		400		792		189		194		383		203		206		409	
**Person years**	1371		1349		2720		645		630		1275		726		719		1446	
Total number of events [Number of participants]	13	[11]	15	[15]	28	[26]	10	[8]	11	[11]	21	[19]	3	[3]	4	[4]	7	[7]
Severe WHO 3	0	[0]	1	[1]	1	[1]	0	[0]	0	[0]	0	[0]	0	[0]	1	[1]	1	[1]
WHO 4	11	[9]	8	[8]	19	[17]	8	[6]	7	[7]	15	[13]	3	[3]	1	[1]	4	[4]
Death	4	[4]	7	[7]	11	[11]	4	[4]	5	[5]	9	[9]	0	[0]	2	[2]	2	[2]
**Infectious Disease**	8	[6]	11	[11]	19	[17]	7	[5]	8	[8]	15	[13]	1	[1]	3	[3]	4	[4]
Bronchiectasis	0	[0]	1	[1]	1	[1]	0	[0]	0	[0]	0	[0]	0	[0]	1	[1]	1	[1]
Candidiasis of oesophagus, trachea, bronchi or lungs	0	[0]	1	[1]	1	[1]	0	[0]	0	[0]	0	[0]	0	[0]	1	[1]	1	[1]
Cryptococcal meningitis	2	[1]	0	[0]	2	[1]	2	[1]	0	[0]	2	[1]	0	[0]	0	[0]	0	[0]
Gastroenteritis *	0	[0]	1	[1]	1	[1]	0	[0]	0	[0]	0	[0]	0	[0]	1	[1]	1	[1]
Pneumonia - other bacterial+Presumed septicaemia/bacteremia - not investigated *	0	[0]	1	[1]	1	[1]	0	[0]	1	[1]	1	[1]	0	[0]	0	[0]	0	[0]
Pneumonia no organism identified, aspiration pneumonia *	0	[0]	1	[1]	1	[1]	0	[0]	1	[1]	1	[1]	0	[0]	0	[0]	0	[0]
Pneumonia no organism identified, aspiration pneumonia+Candidiasis of oesophagus, trachea, bronchi or lungs	0	[0]	1	[1]	1	[1]	0	[0]	1	[1]	1	[1]	0	[0]	0	[0]	0	[0]
Presumed septicaemia/bacteremia - no organism+Tuberculosis - disseminated/miliary	0	[0]	1	[1]	1	[1]	0	[0]	1	[1]	1	[1]	0	[0]	0	[0]	0	[0]
Tuberculosis - abdominal	0	[0]	1	[1]	1	[1]	0	[0]	1	[1]	1	[1]	0	[0]	0	[0]	0	[0]

Tuberculosis - disseminated/miliary	5	[4]	2	[2]	7	[6]	4	[3]	2	[2]	6	[5]	1	[1]	0	[0]	1	[1]
Tuberculosis - disseminated/miliary *	1	[1]	0	[0]	1	[1]	1	[1]	0	[0]	1	[1]	0	[0]	0	[0]	0	[0]
Tuberculosis - pulmonary - smear negative or not done+Severe malnutrition *	0	[0]	1	[1]	1	[1]	0	[0]	1	[1]	1	[1]	0	[0]	0	[0]	0	[0]
**Non HIV related deaths**	1	[1]	0	[0]	1	[1]	1	[1]	0	[0]	1	[1]	0	[0]	0	[0]	0	[0]
Traumatic *	1	[1]	0	[0]	1	[1]	1	[1]	0	[0]	1	[1]	0	[0]	0	[0]	0	[0]
**Other**	0	[0]	1	[1]	1	[1]	0	[0]	0	[0]	0	[0]	0	[0]	1	[1]	1	[1]
Death, cause unknown	0	[0]	1	[1]	1	[1]	0	[0]	0	[0]	0	[0]	0	[0]	1	[1]	1	[1]
**Renal**	1	[1]	0	[0]	1	[1]	1	[1]	0	[0]	1	[1]	0	[0]	0	[0]	0	[0]
Renal failure - chronic *	1	[1]	0	[0]	1	[1]	1	[1]	0	[0]	1	[1]	0	[0]	0	[0]	0	[0]
**Systemic**	2	[2]	2	[2]	4	[4]	1	[1]	2	[2]	3	[3]	1	[1]	0	[0]	1	[1]
Chest infection+Severe malnutrition	1	[1]	0	[0]	1	[1]	0	[0]	0	[0]	0	[0]	1	[1]	0	[0]	1	[1]
Kwashiorkor *	1	[1]	0	[0]	1	[1]	1	[1]	0	[0]	1	[1]	0	[0]	0	[0]	0	[0]
Severe malnutrition *	0	[0]	1	[1]	1	[1]	0	[0]	1	[1]	1	[1]	0	[0]	0	[0]	0	[0]
Wasting syndrome uninvestigated+Oral candida+Chronic diarrhoea not investigated	0	[0]	1	[1]	1	[1]	0	[0]	1	[1]	1	[1]	0	[0]	0	[0]	0	[0]
**Tumours**	1	[1]	1	[1]	2	[2]	0	[0]	1	[1]	1	[1]	1	[1]	0	[0]	1	[1]
Kaposi's sarcoma cutaneous	1	[1]	0	[0]	1	[1]	0	[0]	0	[0]	0	[0]	1	[1]	0	[0]	1	[1]
Non Hodgkin lymphoma *	0	[0]	1	[1]	1	[1]	0	[0]	1	[1]	1	[1]	0	[0]	0	[0]	0	[0]

Ω No participants experienced severe WHO 3 event, WHO 4 event or death after switching off randomised allocation.

*Resulted in death

Table S11. Intention-to-treat: Serious adverse events to 192 weeks

	Total						A						B					
	DTG		SOC		Total		DTG		SOC		Total		DTG		SOC		Total	
**Participants randomised and included**	392		400		792		189		194		383		203		206		409	
**Person years**	1371		1349		2720		645		630		1275		726		719		1446	
Total number of events [Number of participants]Ω	85	[52]	66	[53]	151	[105]	68	[36]	51	[38]	119	[74]	17	[16]	15	[15]	32	[31]
Death	4	[4]	7	[7]	11	[11]	4	[4]	5	[5]	9	[9]	0	[0]	2	[2]	2	[2]
Life-threatening	7	[4]	1	[1]	8	[5]	7	[4]	1	[1]	8	[5]	0	[0]	0	[0]	0	[0]
Hospitalisation	68	[44]	56	[44]	124	[88]	52	[29]	43	[31]	95	[60]	16	[15]	13	[13]	29	[28]
Significant disability	2	[2]	0	[0]	2	[2]	2	[2]	0	[0]	2	[2]	0	[0]	0	[0]	0	[0]
Other	4	[4]	2	[2]	6	[6]	3	[3]	2	[2]	5	[5]	1	[1]	0	[0]	1	[1]
**Biochemical**	2	[2]	1	[1]	3	[3]	2	[2]	1	[1]	3	[3]	0	[0]	0	[0]	0	[0]
Hyperkalaemia	1	[1]	0	[0]	1	[1]	1	[1]	0	[0]	1	[1]	0	[0]	0	[0]	0	[0]
Raised creatinine	1	[1]	0	[0]	1	[1]	1	[1]	0	[0]	1	[1]	0	[0]	0	[0]	0	[0]
Raised liver enzymes	0	[0]	1	[1]	1	[1]	0	[0]	1	[1]	1	[1]	0	[0]	0	[0]	0	[0]
**Cardiovascular**	3	[2]	0	[0]	3	[2]	3	[2]	0	[0]	3	[2]	0	[0]	0	[0]	0	[0]
Congestive cardiac failure	2	[1]	0	[0]	2	[1]	2	[1]	0	[0]	2	[1]	0	[0]	0	[0]	0	[0]
Deep vein thrombosis	1	[1]	0	[0]	1	[1]	1	[1]	0	[0]	1	[1]	0	[0]	0	[0]	0	[0]
**Haematological**	13	[4]	5	[4]	18	[8]	11	[2]	3	[2]	14	[4]	2	[2]	2	[2]	4	[4]
Anaemia with clinical symptoms	11	[2]	4	[3]	15	[5]	10	[1]	2	[1]	12	[2]	1	[1]	2	[2]	3	[3]
Thrombocytopenia	0	[0]	1	[1]	1	[1]	0	[0]	1	[1]	1	[1]	0	[0]	0	[0]	0	[0]
Thrombocytopenia+Neutropenia	2	[2]	0	[0]	2	[2]	1	[1]	0	[0]	1	[1]	1	[1]	0	[0]	1	[1]
**Hepatic**	1	[1]	0	[0]	1	[1]	1	[1]	0	[0]	1	[1]	0	[0]	0	[0]	0	[0]
drug induced liver injury	1	[1]	0	[0]	1	[1]	1	[1]	0	[0]	1	[1]	0	[0]	0	[0]	0	[0]
**Immune System Disorder**	1	[1]	0	[0]	1	[1]	1	[1]	0	[0]	1	[1]	0	[0]	0	[0]	0	[0]
Hypersensitivity reaction	1	[1]	0	[0]	1	[1]	1	[1]	0	[0]	1	[1]	0	[0]	0	[0]	0	[0]
**Infectious Disease**	44	[38]	41	[33]	85	[71]	34	[28]	30	[22]	64	[50]	10	[10]	11	[11]	21	[21]
Acute diarrhoea not investigated	1	[1]	1	[1]	2	[2]	1	[1]	1	[1]	2	[2]	0	[0]	0	[0]	0	[0]
Acute diarrhoea not investigated+Dehydration	1	[1]	0	[0]	1	[1]	1	[1]	0	[0]	1	[1]	0	[0]	0	[0]	0	[0]

Acute diarrhoea not investigated+Renal failure - acute+Thrombocytopenia	1	[1]	0	[0]	1	[1]	1	[1]	0	[0]	1	[1]	0	[0]	0	[0]	0	[0]
Acute febrile episode - undiagnosed	3	[3]	1	[1]	4	[4]	3	[3]	1	[1]	4	[4]	0	[0]	0	[0]	0	[0]
Acute hepatitis	0	[0]	1	[1]	1	[1]	0	[0]	0	[0]	0	[0]	0	[0]	1	[1]	1	[1]
Acute parotitis	1	[1]	0	[0]	1	[1]	1	[1]	0	[0]	1	[1]	0	[0]	0	[0]	0	[0]
Acute sinusitis	1	[1]	0	[0]	1	[1]	0	[0]	0	[0]	0	[0]	1	[1]	0	[0]	1	[1]
Appendicitis	1	[1]	2	[1]	3	[2]	1	[1]	2	[1]	3	[2]	0	[0]	0	[0]	0	[0]
Bronchiectasis	0	[0]	1	[1]	1	[1]	0	[0]	0	[0]	0	[0]	0	[0]	1	[1]	1	[1]
Bronchiolitis	0	[0]	1	[1]	1	[1]	0	[0]	1	[1]	1	[1]	0	[0]	0	[0]	0	[0]
Chest infection	2	[2]	2	[2]	4	[4]	1	[1]	1	[1]	2	[2]	1	[1]	1	[1]	2	[2]
Chronic diarrhoea with no pathogen	1	[1]	0	[0]	1	[1]	1	[1]	0	[0]	1	[1]	0	[0]	0	[0]	0	[0]
Cryptococcal meningitis	2	[1]	0	[0]	2	[1]	2	[1]	0	[0]	2	[1]	0	[0]	0	[0]	0	[0]
Cutaneous warts, Human Papillomavirus	0	[0]	1	[1]	1	[1]	0	[0]	1	[1]	1	[1]	0	[0]	0	[0]	0	[0]
Encephalitis - presumed infectious	1	[1]	0	[0]	1	[1]	1	[1]	0	[0]	1	[1]	0	[0]	0	[0]	0	[0]
Gastroenteritis	2	[2]	2	[2]	4	[4]	2	[2]	2	[2]	4	[4]	0	[0]	0	[0]	0	[0]
Gastroenteritis *	0	[0]	1	[1]	1	[1]	0	[0]	0	[0]	0	[0]	0	[0]	1	[1]	1	[1]
Herpes encephalitis	1	[1]	0	[0]	1	[1]	1	[1]	0	[0]	1	[1]	0	[0]	0	[0]	0	[0]
Measles	1	[1]	2	[2]	3	[3]	0	[0]	1	[1]	1	[1]	1	[1]	1	[1]	2	[2]
Measles+Chest infection	1	[1]	0	[0]	1	[1]	1	[1]	0	[0]	1	[1]	0	[0]	0	[0]	0	[0]
Other gram positive sepsis	1	[1]	0	[0]	1	[1]	1	[1]	0	[0]	1	[1]	0	[0]	0	[0]	0	[0]
P falciparum malaria	8	[6]	0	[0]	8	[6]	5	[3]	0	[0]	5	[3]	3	[3]	0	[0]	3	[3]
Pneumonia - other bacterial	2	[2]	5	[5]	7	[7]	2	[2]	2	[2]	4	[4]	0	[0]	3	[3]	3	[3]
Pneumonia - other bacterial+Presumed septicaemia/bacteremia - not investigated *	0	[0]	1	[1]	1	[1]	0	[0]	1	[1]	1	[1]	0	[0]	0	[0]	0	[0]
Pneumonia no organism identified, aspiration pneumonia	2	[2]	5	[5]	7	[7]	1	[1]	3	[3]	4	[4]	1	[1]	2	[2]	3	[3]

Pneumonia no organism identified, aspiration pneumonia *	0	[0]	1	[1]	1	[1]	0	[0]	1	[1]	1	[1]	0	[0]	0	[0]	0	[0]
Pneumonia no organism identified, aspiration pneumonia+Acute otitis media	1	[1]	0	[0]	1	[1]	1	[1]	0	[0]	1	[1]	0	[0]	0	[0]	0	[0]
Pneumonia no organism identified, aspiration pneumonia+Candidiasis of oesophagus, trachea, bronchi or lungs	0	[0]	1	[1]	1	[1]	0	[0]	1	[1]	1	[1]	0	[0]	0	[0]	0	[0]
Pneumonia no organism identified, aspiration pneumonia+Tuberculosis - pulmonary - smear negative or not done	1	[1]	0	[0]	1	[1]	1	[1]	0	[0]	1	[1]	0	[0]	0	[0]	0	[0]
Presumed septicaemia/bacteremia - no organism	0	[0]	2	[2]	2	[2]	0	[0]	1	[1]	1	[1]	0	[0]	1	[1]	1	[1]
Presumed septicaemia/bacteremia - no organism+Tuberculosis - disseminated/miliary	0	[0]	1	[1]	1	[1]	0	[0]	1	[1]	1	[1]	0	[0]	0	[0]	0	[0]
Pyogenic meningitis - organism	0	[0]	1	[1]	1	[1]	0	[0]	1	[1]	1	[1]	0	[0]	0	[0]	0	[0]
Septic abortion	1	[1]	0	[0]	1	[1]	0	[0]	0	[0]	0	[0]	1	[1]	0	[0]	1	[1]
Septic arthritis+Uveitis	0	[0]	1	[1]	1	[1]	0	[0]	1	[1]	1	[1]	0	[0]	0	[0]	0	[0]
Skin abscess	1	[1]	0	[0]	1	[1]	0	[0]	0	[0]	0	[0]	1	[1]	0	[0]	1	[1]
Tuberculosis - abdominal	0	[0]	1	[1]	1	[1]	0	[0]	1	[1]	1	[1]	0	[0]	0	[0]	0	[0]
Tuberculosis - disseminated/miliary	4	[4]	3	[2]	7	[6]	3	[3]	3	[2]	6	[5]	1	[1]	0	[0]	1	[1]
Tuberculosis - disseminated/miliary *	1	[1]	0	[0]	1	[1]	1	[1]	0	[0]	1	[1]	0	[0]	0	[0]	0	[0]
Tuberculosis - pulmonary - smear negative or not done	1	[1]	1	[1]	2	[2]	1	[1]	1	[1]	2	[2]	0	[0]	0	[0]	0	[0]

Tuberculosis - pulmonary - smear negative or not done+Severe malnutrition	0	[0]	2	[1]	2	[1]	0	[0]	2	[1]	2	[1]	0	[0]	0	[0]	0	[0]
Tuberculosis - pulmonary - smear negative or not done+Severe malnutrition *	0	[0]	1	[1]	1	[1]	0	[0]	1	[1]	1	[1]	0	[0]	0	[0]	0	[0]
Tuberculosis - pulmonary - smear positive	1	[1]	0	[0]	1	[1]	1	[1]	0	[0]	1	[1]	0	[0]	0	[0]	0	[0]
**Lower respiratory tract**	0	[0]	1	[1]	1	[1]	0	[0]	1	[1]	1	[1]	0	[0]	0	[0]	0	[0]
Pneumothorax+bronchiolitis obliterans	0	[0]	1	[1]	1	[1]	0	[0]	1	[1]	1	[1]	0	[0]	0	[0]	0	[0]
**Musculoskeletal**	3	[3]	1	[1]	4	[4]	2	[2]	1	[1]	3	[3]	1	[1]	0	[0]	1	[1]
Bone fracture	3	[3]	1	[1]	4	[4]	2	[2]	1	[1]	3	[3]	1	[1]	0	[0]	1	[1]
**Nervous System**	3	[3]	6	[5]	9	[8]	2	[2]	6	[5]	8	[7]	1	[1]	0	[0]	1	[1]
Dizziness	0	[0]	1	[1]	1	[1]	0	[0]	1	[1]	1	[1]	0	[0]	0	[0]	0	[0]
Epilepsy, fits, convulsions	2	[2]	4	[3]	6	[5]	1	[1]	4	[3]	5	[4]	1	[1]	0	[0]	1	[1]
Headache+Hypertension	1	[1]	0	[0]	1	[1]	1	[1]	0	[0]	1	[1]	0	[0]	0	[0]	0	[0]
head injury	0	[0]	1	[1]	1	[1]	0	[0]	1	[1]	1	[1]	0	[0]	0	[0]	0	[0]
**Non HIV related deaths**	1	[1]	1	[1]	2	[2]	1	[1]	1	[1]	2	[2]	0	[0]	0	[0]	0	[0]
Traumatic *	1	[1]	0	[0]	1	[1]	1	[1]	0	[0]	1	[1]	0	[0]	0	[0]	0	[0]
Traumatic+Cutaneous warts, Human Papillomavirus	0	[0]	1	[1]	1	[1]	0	[0]	1	[1]	1	[1]	0	[0]	0	[0]	0	[0]
**Oral**	0	[0]	1	[1]	1	[1]	0	[0]	1	[1]	1	[1]	0	[0]	0	[0]	0	[0]
Mouth ulcers	0	[0]	1	[1]	1	[1]	0	[0]	1	[1]	1	[1]	0	[0]	0	[0]	0	[0]
**Other**	0	[0]	2	[2]	2	[2]	0	[0]	1	[1]	1	[1]	0	[0]	1	[1]	1	[1]
Death, cause unknown	0	[0]	1	[1]	1	[1]	0	[0]	0	[0]	0	[0]	0	[0]	1	[1]	1	[1]
Non-fatal trauma	0	[0]	1	[1]	1	[1]	0	[0]	1	[1]	1	[1]	0	[0]	0	[0]	0	[0]
**Pregnancy associated**	0	[0]	1	[1]	1	[1]	0	[0]	1	[1]	1	[1]	0	[0]	0	[0]	0	[0]
Spontaneous abortion (complete or incomplete)	0	[0]	1	[1]	1	[1]	0	[0]	1	[1]	1	[1]	0	[0]	0	[0]	0	[0]
**Psychiatric**	3	[2]	2	[2]	5	[4]	3	[2]	2	[2]	5	[4]	0	[0]	0	[0]	0	[0]
Depression	1	[1]	0	[0]	1	[1]	1	[1]	0	[0]	1	[1]	0	[0]	0	[0]	0	[0]
Parasuicide (suicide attempt)	1	[1]	2	[2]	3	[3]	1	[1]	2	[2]	3	[3]	0	[0]	0	[0]	0	[0]
Psychosis, mania	1	[1]	0	[0]	1	[1]	1	[1]	0	[0]	1	[1]	0	[0]	0	[0]	0	[0]

**Renal**	4	[3]	0	[0]	4	[3]	4	[3]	0	[0]	4	[3]	0	[0]	0	[0]	0	[0]
Renal failure - acute	2	[2]	0	[0]	2	[2]	2	[2]	0	[0]	2	[2]	0	[0]	0	[0]	0	[0]
Renal failure - chronic	1	[1]	0	[0]	1	[1]	1	[1]	0	[0]	1	[1]	0	[0]	0	[0]	0	[0]
Renal failure - chronic *	1	[1]	0	[0]	1	[1]	1	[1]	0	[0]	1	[1]	0	[0]	0	[0]	0	[0]
**Skin**	2	[2]	1	[1]	3	[3]	1	[1]	1	[1]	2	[2]	1	[1]	0	[0]	1	[1]
Burns	1	[1]	0	[0]	1	[1]	0	[0]	0	[0]	0	[0]	1	[1]	0	[0]	1	[1]
Rash, erythematous	0	[0]	1	[1]	1	[1]	0	[0]	1	[1]	1	[1]	0	[0]	0	[0]	0	[0]
Rash, maculopapular+URTI - not sinusitis or otitis media - acute	1	[1]	0	[0]	1	[1]	1	[1]	0	[0]	1	[1]	0	[0]	0	[0]	0	[0]
**Systemic**	4	[4]	1	[1]	5	[5]	3	[3]	1	[1]	4	[4]	1	[1]	0	[0]	1	[1]
Chest infection+Severe malnutrition	1	[1]	0	[0]	1	[1]	0	[0]	0	[0]	0	[0]	1	[1]	0	[0]	1	[1]
Kwashiorkor	2	[2]	0	[0]	2	[2]	2	[2]	0	[0]	2	[2]	0	[0]	0	[0]	0	[0]
Kwashiorkor *	1	[1]	0	[0]	1	[1]	1	[1]	0	[0]	1	[1]	0	[0]	0	[0]	0	[0]
Severe malnutrition *	0	[0]	1	[1]	1	[1]	0	[0]	1	[1]	1	[1]	0	[0]	0	[0]	0	[0]
**Tumours**	1	[1]	2	[2]	3	[3]	0	[0]	1	[1]	1	[1]	1	[1]	1	[1]	2	[2]
Hodgkin lymphoma	0	[0]	1	[1]	1	[1]	0	[0]	0	[0]	0	[0]	0	[0]	1	[1]	1	[1]
Kaposi's sarcoma cutaneous	1	[1]	0	[0]	1	[1]	0	[0]	0	[0]	0	[0]	1	[1]	0	[0]	1	[1]
Non Hodgkin lymphoma *	0	[0]	1	[1]	1	[1]	0	[0]	1	[1]	1	[1]	0	[0]	0	[0]	0	[0]

Ω 3 participants in the DTG arm had 3 SAEs after switching off randomised allocation: Pneumonia - other bacterial (n=1); Psychosis, mania (n=1); Septic abortion (n=1). 1 participant in the SOC arm had 1 SAE after switching off randomised allocation: Epilepsy, fits, convulsions (n=1).

*Resulted in death

Table S12. Intention-to-treat: Grade 3 or above clinical and laboratory adverse events to 192 weeks

	**Total**						**A**						**B**					
	**DTG**		**SOC**		**Total**		**DTG**		**SOC**		**Total**		**DTG**		**SOC**		**Total**	
**Participants randomised and included**	392		400		792		189		194		383		203		206		409	
**Person years**	1371		1349		2720		645		630		1275		726		719		1446	
**Total number of events [Number of participants] Ω**	157	[98]	192	[121]	349	[219]	113	[67]	104	[68]	217	[135]	44	[31]	88	[53]	132	[84]
**Biochemical**	22	[21]	66	[44]	88	[65]	15	[14]	16	[14]	31	[28]	7	[7]	50	[30]	57	[37]
Hyperkalaemia	1	[1]	0	[0]	1	[1]	1	[1]	0	[0]	1	[1]	0	[0]	0	[0]	0	[0]
Hypocalcaemia - asymptomatic	2	[2]	5	[5]	7	[7]	2	[2]	1	[1]	3	[3]	0	[0]	4	[4]	4	[4]
Hypocalcaemia - clinically symptomatic	1	[1]	0	[0]	1	[1]	1	[1]	0	[0]	1	[1]	0	[0]	0	[0]	0	[0]
Hypoglycaemia	0	[0]	2	[2]	2	[2]	0	[0]	1	[1]	1	[1]	0	[0]	1	[1]	1	[1]
Hypophosphataemia	1	[1]	1	[1]	2	[2]	1	[1]	0	[0]	1	[1]	0	[0]	1	[1]	1	[1]
Proteinuria	2	[2]	0	[0]	2	[2]	0	[0]	0	[0]	0	[0]	2	[2]	0	[0]	2	[2]
Raised ALT	1	[1]	3	[3]	4	[4]	1	[1]	3	[3]	4	[4]	0	[0]	0	[0]	0	[0]
Raised AST	2	[2]	1	[1]	3	[3]	1	[1]	1	[1]	2	[2]	1	[1]	0	[0]	1	[1]
Raised LDL	0	[0]	1	[1]	1	[1]	0	[0]	1	[1]	1	[1]	0	[0]	0	[0]	0	[0]
Raised alkaline phosphatase (ALK)	1	[1]	0	[0]	1	[1]	1	[1]	0	[0]	1	[1]	0	[0]	0	[0]	0	[0]
Raised bilirubin	2	[2]	45	[28]	47	[30]	1	[1]	5	[4]	6	[5]	1	[1]	40	[24]	41	[25]
Raised cholesterol	0	[0]	2	[2]	2	[2]	0	[0]	1	[1]	1	[1]	0	[0]	1	[1]	1	[1]
Raised creatinine	6	[6]	1	[1]	7	[7]	5	[5]	0	[0]	5	[5]	1	[1]	1	[1]	2	[2]
Raised liver enzymes	2	[2]	2	[2]	4	[4]	0	[0]	2	[2]	2	[2]	2	[2]	0	[0]	2	[2]
Raised triglycerides	1	[1]	1	[1]	2	[2]	1	[1]	0	[0]	1	[1]	0	[0]	1	[1]	1	[1]
Raised tryglycerides	0	[0]	1	[1]	1	[1]	0	[0]	0	[0]	0	[0]	0	[0]	1	[1]	1	[1]
raised low density lipoprotein	0	[0]	1	[1]	1	[1]	0	[0]	1	[1]	1	[1]	0	[0]	0	[0]	0	[0]

**Cardiovascular**	3	[3]	0	[0]	3	[3]	3	[3]	0	[0]	3	[3]	0	[0]	0	[0]	0	[0]
Congestive cardiac failure	1	[1]	0	[0]	1	[1]	1	[1]	0	[0]	1	[1]	0	[0]	0	[0]	0	[0]
Deep vein thrombosis	1	[1]	0	[0]	1	[1]	1	[1]	0	[0]	1	[1]	0	[0]	0	[0]	0	[0]
Hypertension	1	[1]	0	[0]	1	[1]	1	[1]	0	[0]	1	[1]	0	[0]	0	[0]	0	[0]
**Eye**	0	[0]	1	[1]	1	[1]	0	[0]	1	[1]	1	[1]	0	[0]	0	[0]	0	[0]
Uveitis	0	[0]	1	[1]	1	[1]	0	[0]	1	[1]	1	[1]	0	[0]	0	[0]	0	[0]
**Haematological**	47	[41]	52	[42]	99	[83]	30	[27]	33	[29]	63	[56]	17	[14]	19	[13]	36	[27]
Anaemia with clinical symptoms	4	[4]	4	[3]	8	[7]	3	[3]	1	[1]	4	[4]	1	[1]	3	[2]	4	[3]
Anaemia with no clinical symptoms	13	[13]	18	[14]	31	[27]	10	[10]	15	[13]	25	[23]	3	[3]	3	[1]	6	[4]
Leucopenia	0	[0]	1	[1]	1	[1]	0	[0]	1	[1]	1	[1]	0	[0]	0	[0]	0	[0]
Low lymphocytes	2	[2]	2	[2]	4	[4]	1	[1]	1	[1]	2	[2]	1	[1]	1	[1]	2	[2]
Neutropenia	16	[14]	13	[12]	29	[26]	11	[10]	7	[7]	18	[17]	5	[4]	6	[5]	11	[9]
Thrombocytopenia	12	[11]	14	[14]	26	[25]	5	[5]	8	[8]	13	[13]	7	[6]	6	[6]	13	[12]
**Hepatic**	1	[1]	1	[1]	2	[2]	1	[1]	1	[1]	2	[2]	0	[0]	0	[0]	0	[0]
Drug Induced Liver Injury	0	[0]	1	[1]	1	[1]	0	[0]	1	[1]	1	[1]	0	[0]	0	[0]	0	[0]
drug induced liver injury	1	[1]	0	[0]	1	[1]	1	[1]	0	[0]	1	[1]	0	[0]	0	[0]	0	[0]
**Immune System Disorder**	1	[1]	0	[0]	1	[1]	1	[1]	0	[0]	1	[1]	0	[0]	0	[0]	0	[0]
Hypersensitivity reaction	1	[1]	0	[0]	1	[1]	1	[1]	0	[0]	1	[1]	0	[0]	0	[0]	0	[0]
**Infectious Disease**	57	[45]	48	[36]	105	[81]	43	[32]	35	[25]	78	[57]	14	[13]	13	[11]	27	[24]
Acute diarrhoea not investigated	4	[4]	1	[1]	5	[5]	4	[4]	1	[1]	5	[5]	0	[0]	0	[0]	0	[0]
Acute febrile episode - undiagnosed	3	[3]	1	[1]	4	[4]	3	[3]	1	[1]	4	[4]	0	[0]	0	[0]	0	[0]
Acute hepatitis	0	[0]	1	[1]	1	[1]	0	[0]	0	[0]	0	[0]	0	[0]	1	[1]	1	[1]
Acute otitis media	1	[1]	0	[0]	1	[1]	1	[1]	0	[0]	1	[1]	0	[0]	0	[0]	0	[0]
Acute sinusitis	1	[1]	0	[0]	1	[1]	0	[0]	0	[0]	0	[0]	1	[1]	0	[0]	1	[1]
Appendicitis	1	[1]	1	[1]	2	[2]	1	[1]	1	[1]	2	[2]	0	[0]	0	[0]	0	[0]
Bronchiectasis	0	[0]	2	[1]	2	[1]	0	[0]	0	[0]	0	[0]	0	[0]	2	[1]	2	[1]

Bronchiolitis	0	[0]	1	[1]	1	[1]	0	[0]	1	[1]	1	[1]	0	[0]	0	[0]	0	[0]
Candidiasis of oesophagus, trachea, bronchi or lungs	0	[0]	1	[1]	1	[1]	0	[0]	1	[1]	1	[1]	0	[0]	0	[0]	0	[0]
Chest infection	4	[3]	2	[2]	6	[5]	2	[2]	1	[1]	3	[3]	2	[1]	1	[1]	3	[2]
Chronic diarrhoea not investigated	0	[0]	2	[2]	2	[2]	0	[0]	2	[2]	2	[2]	0	[0]	0	[0]	0	[0]
Chronic diarrhoea with no pathogen	1	[1]	0	[0]	1	[1]	1	[1]	0	[0]	1	[1]	0	[0]	0	[0]	0	[0]
Cryptococcal meningitis	2	[1]	0	[0]	2	[1]	2	[1]	0	[0]	2	[1]	0	[0]	0	[0]	0	[0]
Encephalitis - presumed infectious	1	[1]	0	[0]	1	[1]	1	[1]	0	[0]	1	[1]	0	[0]	0	[0]	0	[0]
Gastroenteritis	2	[2]	2	[2]	4	[4]	2	[2]	2	[2]	4	[4]	0	[0]	0	[0]	0	[0]
Gastroenteritis *	0	[0]	1	[1]	1	[1]	0	[0]	0	[0]	0	[0]	0	[0]	1	[1]	1	[1]
Gingivitis, bleeding gums, periodontitis, stomatitis - not necrotizing	1	[1]	0	[0]	1	[1]	0	[0]	0	[0]	0	[0]	1	[1]	0	[0]	1	[1]
Hepatitis A	4	[4]	0	[0]	4	[4]	1	[1]	0	[0]	1	[1]	3	[3]	0	[0]	3	[3]
Herpes Zoster (Varicella Zoster) - cutaneous	0	[0]	1	[1]	1	[1]	0	[0]	1	[1]	1	[1]	0	[0]	0	[0]	0	[0]
Herpes encephalitis	1	[1]	0	[0]	1	[1]	1	[1]	0	[0]	1	[1]	0	[0]	0	[0]	0	[0]
Internal abscess	0	[0]	1	[1]	1	[1]	0	[0]	1	[1]	1	[1]	0	[0]	0	[0]	0	[0]
Measles	2	[2]	2	[2]	4	[4]	1	[1]	1	[1]	2	[2]	1	[1]	1	[1]	2	[2]
Oral candida	0	[0]	1	[1]	1	[1]	0	[0]	1	[1]	1	[1]	0	[0]	0	[0]	0	[0]
Other gram positive sepsis	1	[1]	0	[0]	1	[1]	1	[1]	0	[0]	1	[1]	0	[0]	0	[0]	0	[0]
P falciparum malaria	8	[6]	0	[0]	8	[6]	5	[3]	0	[0]	5	[3]	3	[3]	0	[0]	3	[3]
Pneumonia - other bacterial	2	[2]	5	[5]	7	[7]	2	[2]	2	[2]	4	[4]	0	[0]	3	[3]	3	[3]
Pneumonia - other bacterial *	0	[0]	1	[1]	1	[1]	0	[0]	1	[1]	1	[1]	0	[0]	0	[0]	0	[0]

Pneumonia - other organism (not bacterial)	0	[0]	1	[1]	1	[1]	0	[0]	0	[0]	0	[0]	0	[0]	1	[1]	1	[1]
Pneumonia no organism identified, aspiration pneumonia	4	[4]	7	[6]	11	[10]	4	[4]	5	[4]	9	[8]	0	[0]	2	[2]	2	[2]
Pneumonia no organism identified, aspiration pneumonia *	0	[0]	1	[1]	1	[1]	0	[0]	1	[1]	1	[1]	0	[0]	0	[0]	0	[0]
Presumed septicaemia/bacteremia - no organism	0	[0]	3	[3]	3	[3]	0	[0]	2	[2]	2	[2]	0	[0]	1	[1]	1	[1]
Presumed septicaemia/bacteremia - not investigated *	0	[0]	1	[1]	1	[1]	0	[0]	1	[1]	1	[1]	0	[0]	0	[0]	0	[0]
Pyogenic meningitis - organism	0	[0]	1	[1]	1	[1]	0	[0]	1	[1]	1	[1]	0	[0]	0	[0]	0	[0]
Septic abortion	1	[1]	0	[0]	1	[1]	0	[0]	0	[0]	0	[0]	1	[1]	0	[0]	1	[1]
Septic arthritis	0	[0]	1	[1]	1	[1]	0	[0]	1	[1]	1	[1]	0	[0]	0	[0]	0	[0]
Skin abscess	1	[1]	0	[0]	1	[1]	0	[0]	0	[0]	0	[0]	1	[1]	0	[0]	1	[1]
Tuberculosis - abdominal	0	[0]	1	[1]	1	[1]	0	[0]	1	[1]	1	[1]	0	[0]	0	[0]	0	[0]
Tuberculosis - disseminated/miliary	5	[4]	3	[3]	8	[7]	4	[3]	3	[3]	7	[6]	1	[1]	0	[0]	1	[1]
Tuberculosis - disseminated/miliary *	1	[1]	0	[0]	1	[1]	1	[1]	0	[0]	1	[1]	0	[0]	0	[0]	0	[0]
Tuberculosis - pulmonary - smear negative or not done	5	[5]	2	[2]	7	[7]	5	[5]	2	[2]	7	[7]	0	[0]	0	[0]	0	[0]
Tuberculosis - pulmonary - smear negative or not done *	0	[0]	1	[1]	1	[1]	0	[0]	1	[1]	1	[1]	0	[0]	0	[0]	0	[0]
Tuberculosis - pulmonary - smear positive	1	[1]	0	[0]	1	[1]	1	[1]	0	[0]	1	[1]	0	[0]	0	[0]	0	[0]
**Lower respiratory tract**	0	[0]	1	[1]	1	[1]	0	[0]	1	[1]	1	[1]	0	[0]	0	[0]	0	[0]
Pneumothorax	0	[0]	1	[1]	1	[1]	0	[0]	1	[1]	1	[1]	0	[0]	0	[0]	0	[0]

**Musculoskeletal**	3	[3]	2	[2]	5	[5]	2	[2]	1	[1]	3	[3]	1	[1]	1	[1]	2	[2]
Bone fracture	3	[3]	2	[2]	5	[5]	2	[2]	1	[1]	3	[3]	1	[1]	1	[1]	2	[2]
**Nervous System**	6	[6]	6	[6]	12	[12]	4	[4]	5	[5]	9	[9]	2	[2]	1	[1]	3	[3]
Dizziness	0	[0]	1	[1]	1	[1]	0	[0]	1	[1]	1	[1]	0	[0]	0	[0]	0	[0]
Epilepsy, fits, convulsions	4	[4]	4	[4]	8	[8]	2	[2]	3	[3]	5	[5]	2	[2]	1	[1]	3	[3]
Headache	1	[1]	0	[0]	1	[1]	1	[1]	0	[0]	1	[1]	0	[0]	0	[0]	0	[0]
dystonia	1	[1]	0	[0]	1	[1]	1	[1]	0	[0]	1	[1]	0	[0]	0	[0]	0	[0]
head injury	0	[0]	1	[1]	1	[1]	0	[0]	1	[1]	1	[1]	0	[0]	0	[0]	0	[0]
**Non HIV related deaths**	1	[1]	1	[1]	2	[2]	1	[1]	1	[1]	2	[2]	0	[0]	0	[0]	0	[0]
Traumatic	0	[0]	1	[1]	1	[1]	0	[0]	1	[1]	1	[1]	0	[0]	0	[0]	0	[0]
Traumatic *	1	[1]	0	[0]	1	[1]	1	[1]	0	[0]	1	[1]	0	[0]	0	[0]	0	[0]
**Oral**	0	[0]	1	[1]	1	[1]	0	[0]	1	[1]	1	[1]	0	[0]	0	[0]	0	[0]
Mouth ulcers	0	[0]	1	[1]	1	[1]	0	[0]	1	[1]	1	[1]	0	[0]	0	[0]	0	[0]
**Other**	0	[0]	2	[2]	2	[2]	0	[0]	1	[1]	1	[1]	0	[0]	1	[1]	1	[1]
Death, cause unknown	0	[0]	1	[1]	1	[1]	0	[0]	0	[0]	0	[0]	0	[0]	1	[1]	1	[1]
Non-fatal trauma	0	[0]	1	[1]	1	[1]	0	[0]	1	[1]	1	[1]	0	[0]	0	[0]	0	[0]
**Pregnancy associated**	0	[0]	1	[1]	1	[1]	0	[0]	1	[1]	1	[1]	0	[0]	0	[0]	0	[0]
Spontaneous abortion (complete or incomplete)	0	[0]	1	[1]	1	[1]	0	[0]	1	[1]	1	[1]	0	[0]	0	[0]	0	[0]
**Psychiatric**	6	[4]	4	[4]	10	[8]	5	[3]	2	[2]	7	[5]	1	[1]	2	[2]	3	[3]
Depression	1	[1]	0	[0]	1	[1]	1	[1]	0	[0]	1	[1]	0	[0]	0	[0]	0	[0]
Insomnia	1	[1]	0	[0]	1	[1]	1	[1]	0	[0]	1	[1]	0	[0]	0	[0]	0	[0]
Parasuicide (suicide attempt)	2	[2]	4	[4]	6	[6]	2	[2]	2	[2]	4	[4]	0	[0]	2	[2]	2	[2]
Psychosis, mania	1	[1]	0	[0]	1	[1]	1	[1]	0	[0]	1	[1]	0	[0]	0	[0]	0	[0]
Suicidal Ideation	1	[1]	0	[0]	1	[1]	0	[0]	0	[0]	0	[0]	1	[1]	0	[0]	1	[1]
**Renal**	4	[4]	0	[0]	4	[4]	4	[4]	0	[0]	4	[4]	0	[0]	0	[0]	0	[0]
Renal failure - acute	3	[3]	0	[0]	3	[3]	3	[3]	0	[0]	3	[3]	0	[0]	0	[0]	0	[0]
Renal failure - chronic *	1	[1]	0	[0]	1	[1]	1	[1]	0	[0]	1	[1]	0	[0]	0	[0]	0	[0]
**Skin**	1	[1]	1	[1]	2	[2]	0	[0]	1	[1]	1	[1]	1	[1]	0	[0]	1	[1]

Burns	1	[1]	0	[0]	1	[1]	0	[0]	0	[0]	0	[0]	1	[1]	0	[0]	1	[1]
Rash, erythematous	0	[0]	1	[1]	1	[1]	0	[0]	1	[1]	1	[1]	0	[0]	0	[0]	0	[0]
**Systemic**	5	[5]	3	[3]	8	[8]	4	[4]	3	[3]	7	[7]	1	[1]	0	[0]	1	[1]
Dehydration	1	[1]	0	[0]	1	[1]	1	[1]	0	[0]	1	[1]	0	[0]	0	[0]	0	[0]
Kwashiorkor	2	[2]	0	[0]	2	[2]	2	[2]	0	[0]	2	[2]	0	[0]	0	[0]	0	[0]
Kwashiorkor *	1	[1]	0	[0]	1	[1]	1	[1]	0	[0]	1	[1]	0	[0]	0	[0]	0	[0]
Severe malnutrition	1	[1]	0	[0]	1	[1]	0	[0]	0	[0]	0	[0]	1	[1]	0	[0]	1	[1]
Severe malnutrition *	0	[0]	2	[2]	2	[2]	0	[0]	2	[2]	2	[2]	0	[0]	0	[0]	0	[0]
Wasting syndrome uninvestigated	0	[0]	1	[1]	1	[1]	0	[0]	1	[1]	1	[1]	0	[0]	0	[0]	0	[0]
**Tumours**	0	[0]	2	[2]	2	[2]	0	[0]	1	[1]	1	[1]	0	[0]	1	[1]	1	[1]
Hodgkin lymphoma	0	[0]	1	[1]	1	[1]	0	[0]	0	[0]	0	[0]	0	[0]	1	[1]	1	[1]
Non Hodgkin lymphoma *	0	[0]	1	[1]	1	[1]	0	[0]	1	[1]	1	[1]	0	[0]	0	[0]	0	[0]

Ω 4 participants in the DTG arm had 6 grade 3 or above AEs after switching off randomised allocation: Insomnia (n=1); Pneumonia - other bacterial (n=1); Raised bilirubin (n=1); Septic abortion (n=1); Thrombocytopenia (n=1); dystonia (n=1). 2 participants in the SOC arm had 2 grade 3 or above AEs after switching off randomised allocation: Epilepsy, fits, convulsions (n=1); Thrombocytopenia (n=1).

*Resulted in death

Table S13. Intention-to-treat: Adverse events leading to ART modification (any grade) to 192 weeks

	Total			A			B		
	**DTG**	**SOC**	**Total**	**DTG**	**SOC**	**Total**	**DTG**	**SOC**	**Total**
**Participants randomised and included**	392	400	792	189	194	383	203	206	409
**Person years**	1371	1349	2720	645	630	1275	726	719	1446
**Total number of events [Number of participants] Ω**	9 [8]	24 [21]	33 [29]	7 [6]	11 [10]	18 [16]	2 [2]	13 [11]	15 [13]
**Biochemical**	0 [0]	6 [6]	6 [6]	0 [0]	1 [1]	1 [1]	0 [0]	5 [5]	5 [5]
Raised bilirubin	0 [0]	3 [3]	3 [3]	0 [0]	0 [0]	0 [0]	0 [0]	3 [3]	3 [3]
Raised cholesterol	0 [0]	1 [1]	1 [1]	0 [0]	0 [0]	0 [0]	0 [0]	1 [1]	1 [1]
Raised liver enzymes	0 [0]	1 [1]	1 [1]	0 [0]	1 [1]	1 [1]	0 [0]	0 [0]	0 [0]
Raised tryglycerides	0 [0]	1 [1]	1 [1]	0 [0]	0 [0]	0 [0]	0 [0]	1 [1]	1 [1]
Gastrointestinal	0 [0]	1 [1]	1 [1]	0 [0]	1 [1]	1 [1]	0 [0]	0 [0]	0 [0]
Vomiting	0 [0]	1 [1]	1 [1]	0 [0]	1 [1]	1 [1]	0 [0]	0 [0]	0 [0]
**Haematological**	1 [1]	5 [4]	6 [5]	0 [0]	0 [0]	0 [0]	1 [1]	5 [4]	6 [5]
Anaemia with clinical symptoms	1 [1]	2 [2]	3 [3]	0 [0]	0 [0]	0 [0]	1 [1]	2 [2]	3 [3]
Neutropenia	0 [0]	3 [2]	3 [2]	0 [0]	0 [0]	0 [0]	0 [0]	3 [2]	3 [2]
**Immune System Disorder**	1 [1]	2 [2]	3 [3]	1 [1]	2 [2]	3 [3]	0 [0]	0 [0]	0 [0]
Hypersensitivity reaction	1 [1]	2 [2]	3 [3]	1 [1]	2 [2]	3 [3]	0 [0]	0 [0]	0 [0]
**Infectious Disease**	2 [2]	1 [1]	3 [3]	1 [1]	0 [0]	1 [1]	1 [1]	1 [1]	2 [2]

Chronic diarrhoea not investigated	0	[0]	1	[1]	1	[1]	0	[0]	0	[0]	0	[0]	0	[0]	1	[1]	1	[1]
Hepatitis A	2	[2]	0	[0]	2	[2]	1	[1]	0	[0]	1	[1]	1	[1]	0	[0]	1	[1]
**Nervous System**	0	[0]	1	[1]	1	[1]	0	[0]	1	[1]	1	[1]	0	[0]	0	[0]	0	[0]
Dizziness	0	[0]	1	[1]	1	[1]	0	[0]	1	[1]	1	[1]	0	[0]	0	[0]	0	[0]
**Psychiatric**	4	[4]	1	[1]	5	[5]	4	[4]	1	[1]	5	[5]	0	[0]	0	[0]	0	[0]
Depression	2	[2]	0	[0]	2	[2]	2	[2]	0	[0]	2	[2]	0	[0]	0	[0]	0	[0]
Psychosis, mania	1	[1]	0	[0]	1	[1]	1	[1]	0	[0]	1	[1]	0	[0]	0	[0]	0	[0]
Suicidal Ideation	0	[0]	1	[1]	1	[1]	0	[0]	1	[1]	1	[1]	0	[0]	0	[0]	0	[0]
aggressive behaviour	1	[1]	0	[0]	1	[1]	1	[1]	0	[0]	1	[1]	0	[0]	0	[0]	0	[0]
Renal	1	[1]	0	[0]	1	[1]	1	[1]	0	[0]	1	[1]	0	[0]	0	[0]	0	[0]
Renal failure - acute	1	[1]	0	[0]	1	[1]	1	[1]	0	[0]	1	[1]	0	[0]	0	[0]	0	[0]
**Skin**	0	[0]	4	[4]	4	[4]	0	[0]	4	[4]	4	[4]	0	[0]	0	[0]	0	[0]
Gynaecomastia	0	[0]	2	[2]	2	[2]	0	[0]	2	[2]	2	[2]	0	[0]	0	[0]	0	[0]
Rash, erythematous	0	[0]	2	[2]	2	[2]	0	[0]	2	[2]	2	[2]	0	[0]	0	[0]	0	[0]
Systemic	0	[0]	3	[3]	3	[3]	0	[0]	1	[1]	1	[1]	0	[0]	2	[2]	2	[2]
Lipodystrophy	0	[0]	3	[3]	3	[3]	0	[0]	1	[1]	1	[1]	0	[0]	2	[2]	2	[2]

Ω 1 participant in the DTG arm had 1 ART modifying adverse event after switching off randomised allocation: Depression (n=1). 0 participants in the SOC arm had an ART modifying adverse event after switching off randomised allocation.

Table S14. Intention-to-treat: Neuropsychiatric adverse events to 192 weeks

	Total						A						B					
	DTG		SOC		Total		DTG		SOC		Total		DTG		SOC		Total	
**Participants randomised and included**	392		400		792		189		194		383		203		206		409	
**Person years**	1371		1349		2720		645		630		1275		726		719		1446	
**Total number of events [Number of participants]**	20	[17]	16	[10]	36	[27]	15	[12]	11	[6]	26	[18]	5	[5]	5	[4]	10	[9]
Neurological	6	[6]	8	[6]	14	[12]	4	[4]	7	[5]	11	[9]	2	[2]	1	[1]	3	[3]
Psychiatric	14	[12]	8	[5]	22	[17]	11	[9]	4	[2]	15	[11]	3	[3]	4	[3]	7	[6]
**Nervous System**	6	[6]	8	[6]	14	[12]	4	[4]	7	[5]	11	[9]	2	[2]	1	[1]	3	[3]
Dizziness	0	[0]	2	[1]	2	[1]	0	[0]	2	[1]	2	[1]	0	[0]	0	[0]	0	[0]
Epilepsy, fits, convulsions	4	[4]	5	[4]	9	[8]	2	[2]	4	[3]	6	[5]	2	[2]	1	[1]	3	[3]
Headache	1	[1]	0	[0]	1	[1]	1	[1]	0	[0]	1	[1]	0	[0]	0	[0]	0	[0]
dystonia	1	[1]	0	[0]	1	[1]	1	[1]	0	[0]	1	[1]	0	[0]	0	[0]	0	[0]
head injury	0	[0]	1	[1]	1	[1]	0	[0]	1	[1]	1	[1]	0	[0]	0	[0]	0	[0]
**Psychiatric**	14	[12]	8	[5]	22	[17]	11	[9]	4	[2]	15	[11]	3	[3]	4	[3]	7	[6]
Depression	2	[2]	0	[0]	2	[2]	2	[2]	0	[0]	2	[2]	0	[0]	0	[0]	0	[0]
Insomnia	1	[1]	0	[0]	1	[1]	1	[1]	0	[0]	1	[1]	0	[0]	0	[0]	0	[0]
Parasuicide (suicide attempt)	2	[2]	4	[4]	6	[6]	2	[2]	2	[2]	4	[4]	0	[0]	2	[2]	2	[2]
Psychosis, mania	1	[1]	0	[0]	1	[1]	1	[1]	0	[0]	1	[1]	0	[0]	0	[0]	0	[0]
Suicidal Ideation	6	[6]	4	[2]	10	[8]	4	[4]	2	[1]	6	[5]	2	[2]	2	[1]	4	[3]
Violent behaviour/aggression	1	[1]	0	[0]	1	[1]	0	[0]	0	[0]	0	[0]	1	[1]	0	[0]	1	[1]
aggressive behaviour	1	[1]	0	[0]	1	[1]	1	[1]	0	[0]	1	[1]	0	[0]	0	[0]	0	[0]

Ω 3 participants in the DTG arm had 4 neuropsychiatric adverse events after switching off randomised allocation: Depression (n=1); Insomnia (n=1); Suicidal Ideation (n=1); dystonia (n=1). 1 participant in the SOC arm had 1 neuropsychiatric adverse event after switching off randomised allocation: Epilepsy, fits, convulsions (n=1).

Table S15. Intention-to-treat: Details neuropsychiatric adverse events to end of trial

Participant ID	ODYSSEY A/B	Treatment arm	Sex	Age at event	Week in trial	ART regimen at event onset	Event name
**Neurological events**							
12	B	DTG	male	7	68	ABC 3TC DTG	Epilepsy, fits, convulsions
13	A	DTG	male	4	19	ABC 3TC DTG	Epilepsy, fits, convulsions
15**	B	DTG	male	10	95	ABC 3TC DTG	Epilepsy, fits, convulsions
20	A	DTG	male	14	2	ABC 3TC DTG	Epilepsy, fits, convulsions
1	A	DTG	male	10	47	ABC 3TC DTG	Headache
5**	A	DTG	male	14	130	ABC 3TC NVP	Dystonia
3	A	DTG	male	21	228	TDF FTC EFV	Dizziness#
23**	B	DTG	female	20	208	TDF 3TC DTG	Epilepsy, fits, convulsions#
15**	B	DTG	male	12	228	TDF 3TC DTG	Epilepsy, fits, convulsions *#
24**	A	SOC	female	15	12	ABC 3TC EFV	Dizziness
24**	A	SOC	female	17	114	ABC 3TC EFV	Dizziness
29**	A	SOC	male	10	132	ABC 3TC EFV	Epilepsy, fits, convulsions
32	B	SOC	male	16	124	ABC 3TC LOP	Epilepsy, fits, convulsions
38	A	SOC	female	8	126	ABC 3TC DTG	Epilepsy, fits, convulsions
40**	A	SOC	male	7	50	ABC 3TC EFV	Epilepsy, fits, convulsions
40**	A	SOC	male	9	160	ABC 3TC EFV	Epilepsy, fits, convulsions#
30	A	SOC	male	14	156	ZDV 3TC ATV	Head injury#
26	A	SOC	female	22	225	ABC 3TC EFV	Epilepsy, fits, convulsions#
28	A	SOC	female	16	220	TDF 3TC DTG	Epilepsy, fits, convulsions#
29**	A	SOC	male	13	287	ABC 3TC DTG	Epilepsy, fits, convulsions#
27	B	SOC	male	18	237	ABC 3TC LOP	Other CNS disease *#
37	B	SOC	female	18	303	TDF 3TC DTG	Seizure#
**Psychiatric events**							
2	A	DTG	male	19	72	TDF FTC EFV	Depression
14**	A	DTG	female	17	98	TDF 3TC DTG	Depression
5**	A	DTG	male	14	130	ABC 3TC NVP	Insomnia
14**	A	DTG	female	17	96	TDF 3TC DTG	Parasuicide (suicide attempt)

22	A	DTG	female	17	93	TDF FTC DTG	Parasuicide (suicide attempt)
5**	A	DTG	male	11	12	ABC 3TC DTG	Psychosis, mania
6	A	DTG	female	16	156	ABC 3TC DTG	Suicidal Ideation
8	A	DTG	female	20	132	ABC 3TC DTG	Suicidal Ideation
9	A	DTG	male	11	52	ABC 3TC DTG	Suicidal Ideation
11	B	DTG	female	17	107	ABC 3TC DTG	Suicidal Ideation
17	B	DTG	male	15	48	TDF 3TC DTG	Suicidal Ideation
21	A	DTG	male	18	145	ABC 3TC DTG	Suicidal Ideation
23**	B	DTG	female	19	180	TDF 3TC DTG	Violent behaviour/aggression#
7	A	DTG	male	18	190	ABC 3TC DTG	Aggressive behaviour#
10	A	DTG	female	16	230	TDF 3TC DTG	Depression#
4	A	DTG	male	22	313	ABC 3TC DTG	Suicidal Ideation#
19	A	DTG	female	16	215	ABC 3TC DTG	Suicidal Ideation#
18	A	DTG	female	14	276	ABC 3TC DTG	Suicidal behaviour#
5**	A	DTG	male	15	228	TDF 3TC ATV	Suicide *#
16	B	DTG	male	17	294	TDF 3TC DRV DTG	Substance use disorder#
25**	A	SOC	male	18	142	ABC 3TC ATV	Parasuicide (suicide attempt)
34	B	SOC	male	18	80	ZDV 3TC LOP	Parasuicide (suicide attempt)
35	B	SOC	female	20	147	ABC 3TC LOP	Parasuicide (suicide attempt)#
40**	A	SOC	male	8	88	ABC 3TC EFV	Parasuicide (suicide attempt)
25**	A	SOC	male	16	50	ABC 3TC EFV	Suicidal Ideation
25**	A	SOC	male	16	73	ABC 3TC EFV	Suicidal Ideation
36**	B	SOC	male	18	31	TDF FTC LOP	Suicidal Ideation
36**	B	SOC	male	19	70	TDF FTC ATV	Suicidal Ideation
39	A	SOC	male	17	245	ABC 3TC DTG	Psychosis, mania#
33	B	SOC	female	18	205	ZDV 3TC LOP	Suicidal Ideation#
31	B	SOC	female	21	293	TDF 3TC DTG	Suicidal behaviour#

*Resulted in death

** Participants with multiple neuropsychiatric events reported

Events occurring during extended follow-up, and therefore not reported in *Turkova, 2023* [4]

Events highlighted in grey are those occurring post 192 weeks and are therefore not included in intention-to-treat analyses of neuropsychiatric events.

References

1. Royston, P. and Parmar, M.K., *Flexible parametric proportional-hazards and proportional-odds models for censored survival data, with application to prognostic modelling and estimation of treatment effects.* Stat Med, 2002. **21**(15): p. 2175-97.
2. Lambert, P.C. and Royston, P., *Further Development of Flexible Parametric Models for Survival Analysis.* The Stata Journal, 2009. **9**(2): p. 265-290.
3. Farmer, R.E., Daniel, R., Ford, D., et al., *Marginal structural models for repeated measures where intercept and slope are correlated: An application exploring the benefit of nutritional supplements on weight gain in HIV-infected children initiating antiretroviral therapy.* PLOS ONE, 2020. **15**(7): p. e0233877.
4. Turkova, A., White, E., Kekitiinwa, A.R., et al., *Neuropsychiatric manifestations and sleep disturbances with dolutegravir-based antiretroviral therapy versus standard of care in children and adolescents: a secondary analysis of the ODYSSEY trial.* Lancet Child Adolesc Health, 2023. **7**(10): p. 718-727.
